# Endothelial OX40 activation facilitates tumor cell escape from T cell surveillance through S1P/YAP-mediated angiogenesis

**DOI:** 10.1172/JCI186291

**Published:** 2025-03-03

**Authors:** Baoyu He, Rou Zhao, Baogui Zhang, Hongli Pan, Jilan Liu, Lunhua Huang, Yingying Wei, Dong Yang, Jing Liang, Mingyi Wang, Mingsheng Zhao, Sen Wang, Fengyun Dong, Junfeng Zhang, Yanhua Zhang, Xu Zhang, Xiao Zhang, Guanjun Dong, Huabao Xiong, Qingli Bie, Bin Zhang

**Affiliations:** 1 Department of Laboratory Medicine,; 2 Department of Gastrointestinal Surgery, and; 3 Department of Oncology, Affiliated Hospital of Jining Medical University, Jining Medical University, Jining, Shandong, China.; 4 Department of Central Lab, Weihai Municipal Hospital, Shandong University, Weihai, Shandong, China.; 5 Institute of Immunology and Molecular Medicine, Jining Medical University, Jining, Shandong, China.; 6 Shanghai Cancer Center, Fudan University, Shanghai, China.; 7 Key Laboratory of Laboratory Medicine of Jiangsu Province, School of Medicine, Jiangsu University, Zhenjiang, Jiangsu, China.; 8 Department of Oncology, Renji Hospital, School of Medicine, Shanghai Jiao Tong University, Shanghai, China.

**Keywords:** Immunology, Oncology, Cancer immunotherapy, Endothelial cells

## Abstract

Understanding the complexity of the tumor microenvironment is vital for improving immunotherapy outcomes. Here, we report that the T cell costimulatory molecule OX40 was highly expressed in tumor endothelial cells (ECs) and was negatively associated with the prognosis of patients, which is irrelevant to T cell activation. Analysis of conditional OX40 loss- and gain-of-function transgenic mice showed that OX40 signal in ECs counteracted the antitumor effects produced in T cells by promoting angiogenesis. Mechanistically, leucine-rich repeat–containing GPCR5 (Lgr5^+^ ) cancer stem cells induced OX40 expression in tumor ECs via EGF/STAT3 signaling. Activated OX40 interacted with Spns lysolipid transporter 2 (Spns2), obstructing the export of sphingosine 1-phosphate (S1P) and resulting in S1P intracellular accumulation. Increased S1P directly bound to Yes 1–associated protein (YAP), disrupting its interaction with large tumor suppressor kinase 1 (LATS1) and promoting YAP nuclear translocation. Finally, the YAP inhibitor verteporfin enhanced the antitumor effects of the OX40 agonist. Together, these findings reveal an unexpected protumor role of OX40 in ECs, highlighting the effect of nonimmune cell compartments on immunotherapy.

## Abstract 2

He et al report that endothelial OX40 signal activation exerts pro-tumor effects by inducing S1P-YAP mediated angiogenesis, which may counteract T cell-mediated anti-tumor effects.

## Graphical abstract

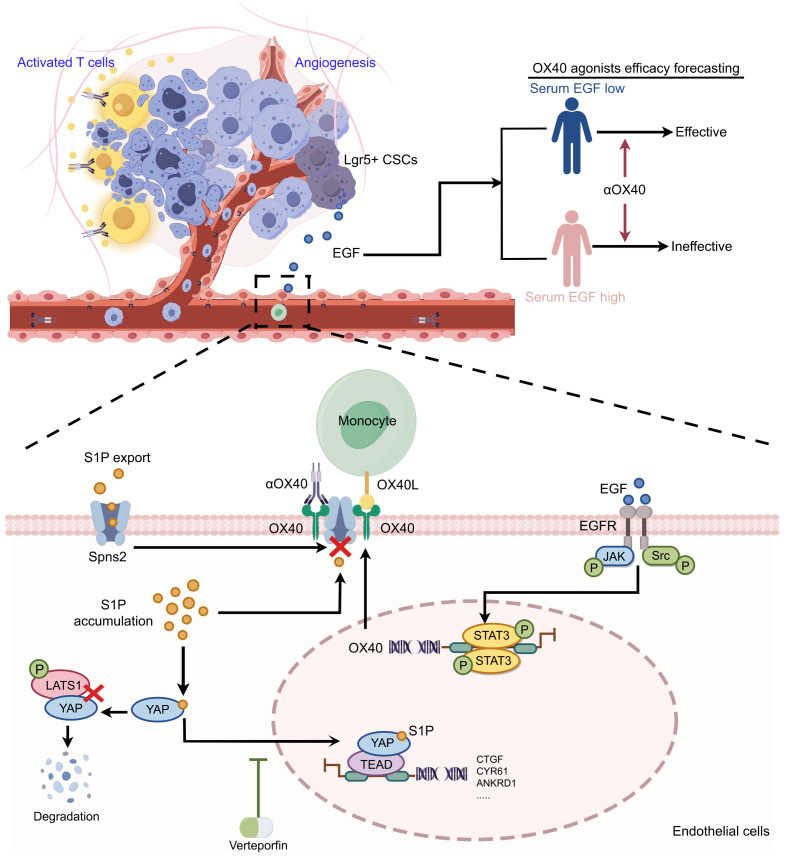

## Introduction

Immune cells are the front line of host defense against tumor development, and T cells play a major role in tumor immunosurveillance (1, 2). Tumor cells evade immune responses from T cells, partly because of the immunosuppressive features of the tumor microenvironment (TME) (3, 4). The TME is a highly heterogeneous milieu composed of various cell types and the extracellular matrix (5), which affects tumor development, progression, and response to treatments (6). Rather than working alone, tumor cells interact closely with the extracellular matrix and stromal cells, forming a favorable TME for the tumor. Within the TME, various immune and non-immune cells together with the cytokines secreted by these cells drive a chronic inflammatory, immunosuppressive, and proangiogenic intratumoral environment (7, 8). Tumor cells can adapt and grow in environments with a markedly lower possibility of being monitored and eliminated by host immune system (9). Recently, strategies targeting the TME have emerged as promising approaches for cancer treatment because of their critical role in regulating tumor progression.

Currently, the foundation of cancer immunotherapy focused on T cells lies in intervention into their functions (10). The primary T cell intervention strategy available clinically is immune checkpoint blockade (ICB) (11, 12). Despite the efficacy of ICB immunotherapy, one subset of cancer patients with low PD-L1 expression develop progressive disease, necessitating additional treatment options (13). To address this question, efforts to improve T cells’ antitumor responses have shifted to triggering costimulatory signals by targeting specific molecules (14). The costimulatory molecule OX40 is the primary focus in this context and plays a vital role in maintaining CD4^+^ and CD8^+^ T cell functions and enhancing T cell–specific cytokine production (15, 16). Pharmaceutical companies have initiated clinical trials of OX40 agonists for tumor immunotherapy. Unexpectedly, almost all OX40 agonistic monotherapies have an objective response rate lower than 10% (17); thus, no OX40 agonists have been investigated further in phase III clinical trials. Moreover, the combined application of OX40 agonists and ICBs can doubly activate T cells in a short period (18), while preliminary clinical data showed that the objective response rate of the combination treatment still does not exceed 13% (17, 19). Thus, it is urgent to unlock the reasons for the failure of OX40 agonists in clinical trials, and additional therapeutic strategies should be developed to address the clinical application dilemma.

Clinical trial data have shown that OX40 agonists enhance antitumor immunity, including activation of CD4^+^ and CD8^+^ T cells and natural killer cells and inhibition of regulatory T cell functions (19, 20). This suggests that OX40 agonists are able to activate the antitumor immunity in vivo. Considering the poor therapeutic effect of OX40 agonists in clinical trials, we speculated that OX40 agonists may reshape the non-immune microenvironment (NIM), which counteracts T cell–mediated antitumor immune responses triggered by OX40 agonists. To clarify the impact of OX40 agonists on NIM, we applied T cell–immunocompromised mice and revealed the unexpected tumor-promoting effects of OX40 activation. We then performed single-cell RNA sequencing (scRNA-Seq), metabolic mass spectrometry analyses, and preclinical studies using patient-derived xenograft models to address the following questions: Which cell subset of NIM bears OX40 function in addition to T cells? Are there any differences in the biological roles of OX40 between NIM and T cells? What are the mechanisms that underlie these differences? How can we address the tumor-promoting effect of OX40 signaling in NIM under T cell activation conditions and design potential strategy for T cell activation therapy? This study provides answers to these questions with a deeper understanding of the oncogenic biology of OX40, laying a theoretical foundation for the combined therapy strategy of T cell activation.

## Results

### OX40 signal exerts an unexpected protumor function in the NIM.

**Figure 1:**
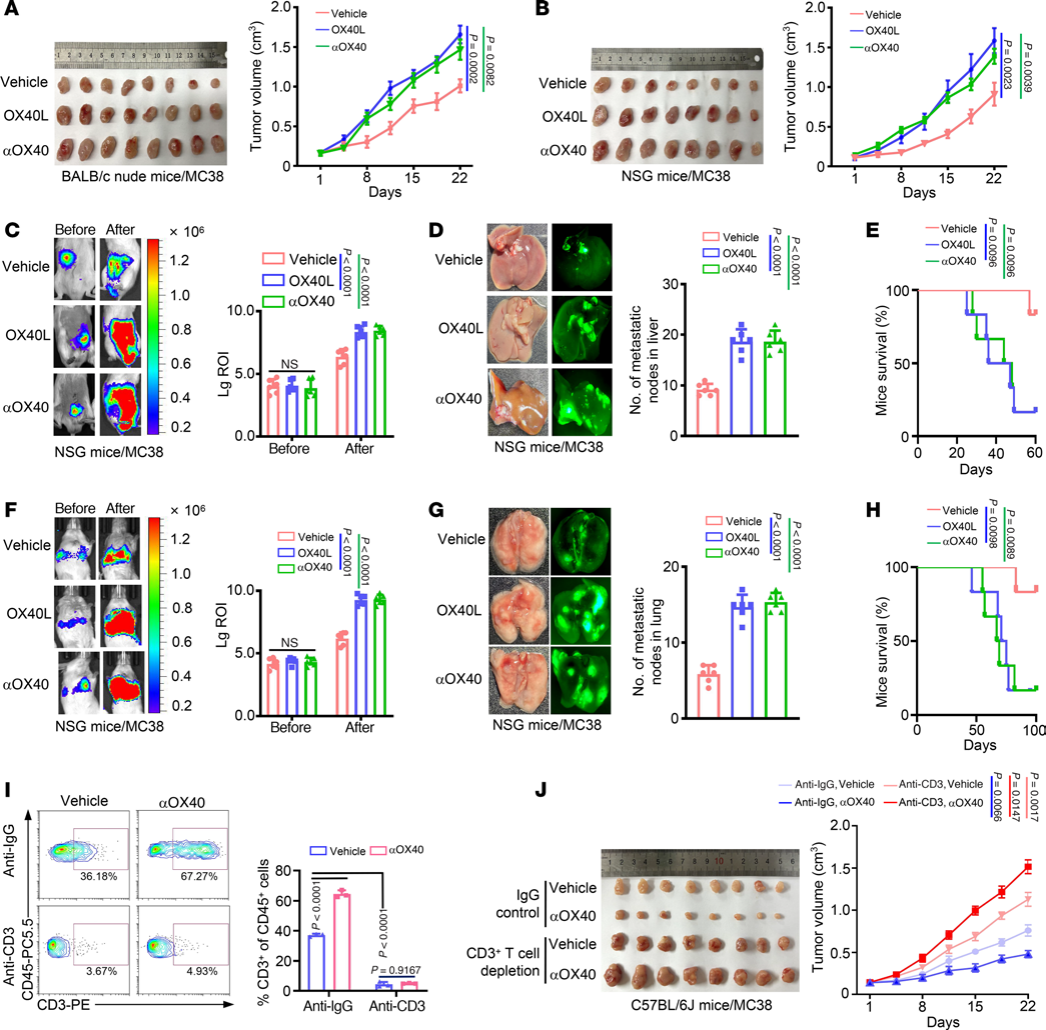
OX40 activation promotes tumor growth and metastasis in T cell–immunocompromised mice. (**A** and **B**) Images (left) and volume (right) of subcutaneous tumors established using MC38 cells treated with mouse OX40L protein (OX40L; 200 mg/mouse, i.p.) or anti–mouse OX40 agonistic antibody (αOX40; 100 μg/mouse, i.p.) in BALB/c nude mice (**A**) and NSG mice (**B**) (*n* = 8). (**C**–**E**) Bioluminescent intensity (**C**), metastatic nodules in the liver (**D**), and survival of mice (**E**) from the colon orthotopic metastasis model established using MC38 cells treated with mouse OX40L protein (OX40L; 200 mg/mouse, i.p.) or anti–mouse OX40 agonistic antibody (αOX40; 100 μg/mouse, i.p.) in NSG mice (*n* = 6). (**F**–**H**) Bioluminescent intensity (**F**), metastatic nodules in the lung (**G**), and survival of mice (**H**) from the pulmonary metastasis model established using MC38 cells treated with mouse OX40L protein (OX40L; 200 mg/mouse, i.p.) or anti–mouse OX40 agonistic antibody (αOX40; 100 μg/mouse, i.p.) in NSG mice (*n* = 6). (**I**) Anti–mouse CD3 antibody (200 μg/mouse, i.p.) was applied to deplete T cells in C57BL/6J mice. Then, CD3^+^ T cells were measured by flow cytometric analysis in CD45^+^ single-cell suspensions sorted from subcutaneous tumors in CD3^+^ T cell–depleted mice and IgG control mice (*n* = 3). (**J**) Images (left) and volume (right) of subcutaneous tumors established using MC38 cells treated with vehicle or anti–mouse OX40 agonistic antibody (αOX40; 100 μg/mouse, i.p.) in IgG mice and CD3^+^ T cell–depleted mice (*n* = 8). Two-way ANOVA (**A**, **B**, and **J**), 1-way ANOVA (**C**, **D**, **F**, **G**, and **I**), or log-rank test (**E** and **H**) was used for statistical analysis. Lg ROI, log_10_ region of interest.

Clinical trial data on OX40 agonists did not show satisfactory antitumor effects, although they activated the immune response (19, 20), hinting at the complex regulatory function in the TME. To clarify the functionality of OX40 activation in non-immune cell compartments, we chose T cell–immunodeficient (BALB/c nude) and severely immunodeficient mice (NSG) and administered OX40 activation, including natural OX40 ligand (OX40L) and anti-OX40 agonistic antibody (αOX40) treatments. Unexpectedly, we observed that OX40 activation promoted tumor growth (Figure 1, A and B). These results indicate that OX40 activation may exert protumor functions in non-immune cell components.

We further validated the tumor-promoting effects of OX40 activation in the metastatic models. We established an orthotopic colon tumor liver metastasis model and a tail-vein injection pulmonary metastasis model in NSG mice, using mouse colon cancer MC38 cells doubly labeled with luciferase (LUC) and green fluorescent protein (GFP) (MC38-LUC-GFP). Luciferase signal intensity showed that OX40 activation resulted in significant liver or lung metastases compared with the corresponding controls (Figure 1, C and F). GFP signal intensity also showed similar results (Figure 1, D and G). Moreover, OX40 activation markedly shortened the survival time of mice in both tumor metastatic models (Figure 1, E and H).

To further substantiate the protumor role of OX40 signaling in NIM, we performed T cell depletion in immunocompetent C57BL/6J mice using the anti-CD3 antibody–mediated in vivo cell depletion method. Subcutaneous tumors were established in this mouse model. Flow cytometry analysis confirmed that anti-CD3 antibody treatment successfully immunodepleted intratumoral CD3^+^ T cells (Figure 1I). First, we observed that T cell depletion potentiated subcutaneous tumor growth, in comparison with the CD3-intact group (Figure 1J). Importantly, in the T cell–intact group, αOX40 treatment markedly inhibited tumor growth, whereas in T cell–depleted mice, αOX40 treatment enhanced tumor growth compared with that in the vehicle group (Figure 1J). Altogether, these data suggest that OX40 signaling in NIM exerts protumor effects.

### OX40 is highly expressed in tumor endothelial cells and promotes tumor progression.

**Figure 2:**
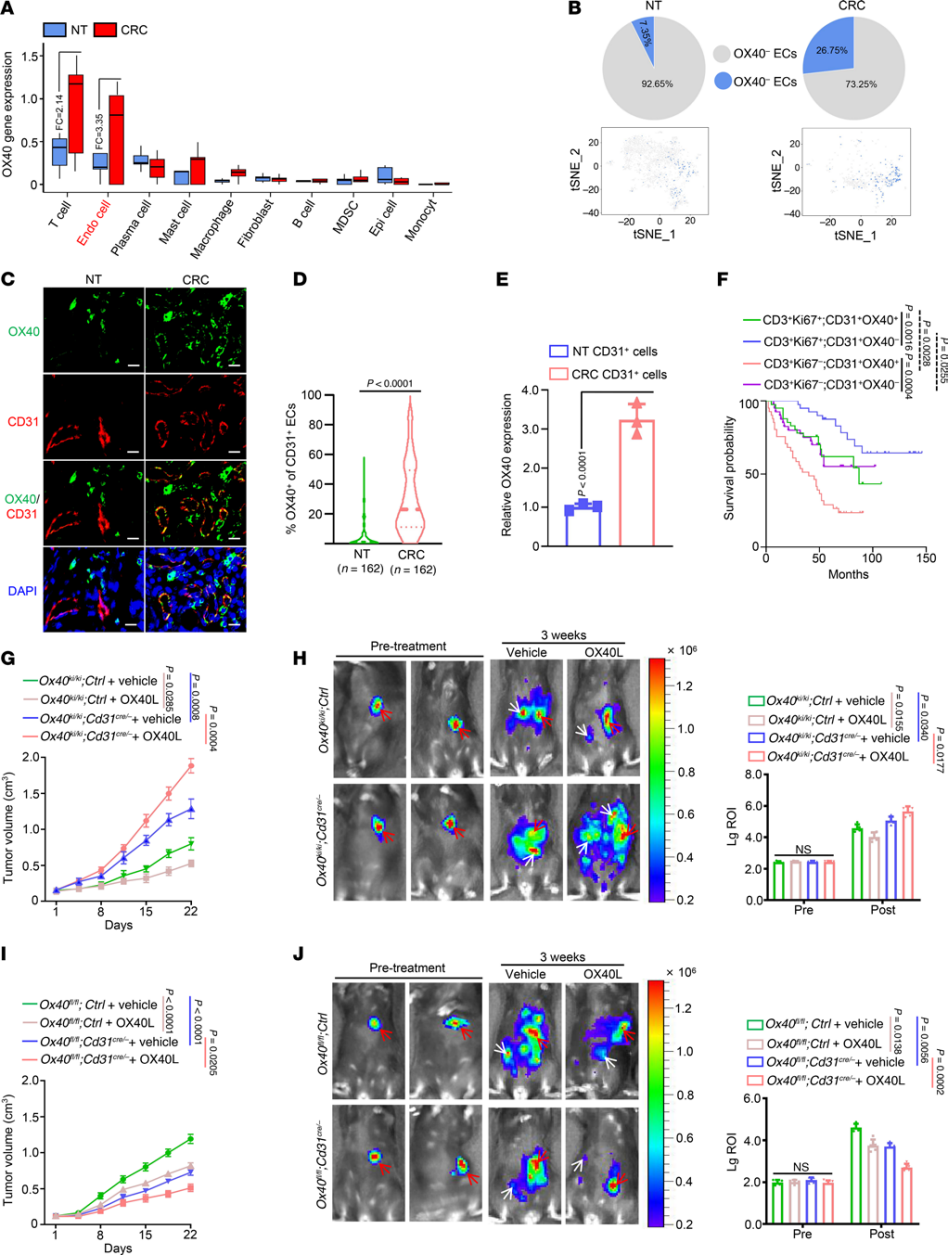
Confirmation of high expression of OX40 specifically in tumor ECs and the protumor effects. (**A**) OX40 gene expression in various cell subpopulations of CRC and NT tissues using scRNA-Seq data (*n* = 5). FC, fold change. (**B**) Proportion of OX40^–^ and OX40^+^ ECs in CRC and NT tissues (*n* = 5). (**C**) Representative images of multicolor immunofluorescence using anti-OX40 (green) and anti-CD31 (red) antibodies in CRC and NT tissues (*n* = 162). Scale bars: 30 μm. (**D**) Analysis of the OX40^+^ EC percentages in CRC and NT tissues. The data were obtained from a tissue microarray, which included 162 pairs of CRC and NT tissues. (**E**) OX40 expression in sorted CD31^+^ cells from CRC and NT tissues (*n* = 3). (**F**) First, 162 CRCs were divided into 2 groups based on the median values of Ki67^+^ percentages in CD3^+^ T cells: CD3^+^Ki67^–^ and CD3^+^Ki67^+^. Further, these 2 groups were divided into 4 parts based on the median values of OX40^+^ percentages in ECs: CD3^+^Ki67^–^;CD31^+^OX40^–^ (*n* = 41), CD3^+^Ki67^–^;CD31^+^OX40^+^ (*n* = 40), CD3^+^Ki67^+^;CD31^+^OX40^–^ (*n* = 41), and CD3^+^Ki67^+^;CD31^+^OX40^+^ (*n* = 40). Kaplan-Meier analysis of the overall survival probability of patients in the 4 groups was performed. (**G**–**J**) C57BL/6J mice with conditional knockin (*Ox40^ki/ki^;Cd31^cre/–^*) or knockout (*Ox40^fl/fl^;Cd31^cre/–^*) of OX40 in ECs were established using the CRISPR/Cas9 method. Subcutaneous tumors (**G** and **I**) and splenic injection for the liver metastasis model (**H** and **J**) were constructed in these mice using MC38 cells and treated with recombinant mouse OX40L (*n* = 5 or 10). In **H** and **J**, the red arrows indicate the implanted primary tumor in the spleen, and the white arrows indicate metastatic tumor lesions. Two-tailed Student’s *t* test (**D** and **E**), log-rank test (**F**), 2-way ANOVA (**G** and **I**), or 1-way ANOVA (**H** and **J**) was used for statistical analysis.

To investigate the molecular mechanisms of OX40’s tumor-promoting functions, we analyzed and compared OX40 expression levels in various cell subpopulations of colorectal cancer (CRC) and corresponding non-tumor (NT) tissues using single-cell transcriptome sequencing (scRNA-Seq). In addition to T cells, OX40 was highly expressed in tumor ECs, and its expression was markedly lower in the other 8 cell subpopulations (Figure 2A). Moreover, OX40 abundance in endothelial cells (ECs) from CRC tissues was markedly higher than that in ECs from NT tissues (Figure 2A). The OX40^+^ percentage of ECs from tumor tissues was 26.75%, whereas that of ECs from NT tissues was 7.35% (Figure 2B). OX40 was more obviously colocalized with the vascular EC marker CD31 in CRC tissues, whereas in NT tissues, OX40 and CD31 colonization was minimal (Figure 2C). Statistically, the percentage of OX40^+^ vascular endothelium was markedly higher in CRC tissues than in NT tissues (Figure 2D). Moreover, OX40 expression was markedly upregulated in CD31^+^ cells from CRC tissues compared with that in the NT group (Figure 2E). We further applied laser microdissection to separate ECs from CRC tissues and evaluated the expression of OX40. Consistent with CD31, OX40 was highly expressed in the vascular ECs (Supplemental Figure 1A; supplemental material available online with this article; https://doi.org/10.1172/JCI186291DS1). Additionally, OX40 was positively correlated with the expression of the canonical EC marker genes CD31, VWF, and CD34 in The Cancer Genome Atlas Colon Adenocarcinoma (TCGA-COAD) dataset (Supplemental Figure 1B). These findings suggest that OX40 is highly expressed in neoplastic ECs and may serve as a potential biomarker for tumor ECs.

Next, we investigated the biological functions of OX40 in ECs. scRNA-Seq data showed that tumor-promoting genes associated with cell proliferation, stemness, metastasis, drug resistance, and key transcription factors (TFs) were upregulated in OX40^+^ ECs from CRC tissues, whereas important tumor suppressors, including RB, BAD, and FAS, were markedly reduced (Supplemental Figure 2A). This implies that OX40 signaling may produce protumor effects in tumor ECs. Then, we investigated the relationship between OX40 expression and patient prognosis. First, tumor patients with active T cell proliferation had a markedly longer survival time (Figure 2F). Interestingly, patients with high OX40 expression in tumor ECs possessed an unfavorable prognosis compared with those with low OX40 expression (Figure 2F). This implies that OX40 signaling may exhibit a protumor function in tumor ECs, which is opposite to the well-known antitumor effects in T cells. To confirm this hypothesis, we analyzed the biological functions of OX40 in ECs using human umbilical vein ECs (HUVECs). OX40 activation after OX40L treatment substantially increased cell proliferative ability (Supplemental Figure 2B), migratory ability (Supplemental Figure 2C), tube-forming capacity (Supplemental Figure 2D), and tumor cell transendothelial migratory ability (Supplemental Figure 2, E and F). Next, we evaluated the impact of OX40 activation on vascular functionality in vivo. Oxygen supply in the TME is an important functional manifestation of the blood vessels (21). The results displayed that the abundance of hypoxia-inducible factor-1α (HIF-1α) in the TME was obviously reduced after OX40 activation (Supplemental Figure 2G).

We further validated the biological functions of OX40 in transgenic mice. First, OX40L treatment enhanced T cell proliferation independent of OX40 expression in ECs (Supplemental Figure 3, A–D), and correspondingly inhibited tumor growth (Figure 2, G and I) and metastasis (Figure 2, H and J) in control mice (*Ox40^ki/ki^;Ctrl* and *Ox40^fl/fl^;Ctrl*). However, conditional OX40 overexpression in ECs promoted angiogenesis (Supplemental Figure 3, A and B), and enhanced tumor growth (Figure 2G) and metastasis (Figure 2H); OX40L treatment further aggravated angiogenesis (Supplemental Figure 3, A and B) and tumor-promoting effects (Figure 2, G and H). Conversely, conditional OX40 knockout in ECs markedly inhibited angiogenesis (Supplemental Figure 3, C and D), tumor growth, and metastasis (Figure 2, I and J). More importantly, OX40L treatment further inhibited tumor growth and metastasis after deletion of OX40 in ECs (Figure 2, I and J). Taken together, these results indicate that OX40 signal in ECs can counteract T cell–mediated antitumor effects by inducing angiogenesis.

To clarify the protumor role of OX40 in pan-cancer ECs, we analyzed open-access data from the Tumor Immune Single-cell Hub single-cell transcriptome database and identified several tumor types with high OX40 expression in the EC subgroup, especially in colon cancer, ovarian cancer, and glioma (Supplemental Figure 4A). We have shown the protumor role of NIM in colon cancer; thus, we further validated this effect in 2 other cancer models. As in colon cancer, OX40 activation promoted tumor growth (Supplemental Figure 4, B and C) and metastasis (Supplemental Figure 4, D, E, G, and H) in ovarian cancer and glioma. Moreover, OX40 activation reduced the survival time of mice in metastatic models (Supplemental Figure 4, F and I). Next, we evaluated angiogenesis after OX40 activation in these tumors. We observed higher vascular density in both OX40L- and αOX40-treated subcutaneous tumors (Supplemental Figure 4, J and K). Together, these data suggest that OX40 signal exerts protumor and pro-angiogenesis effects in tumor ECs.

### Lgr5^+^ tumor stem cells trigger OX40 gene transcriptional activity specifically in tumor ECs via paracrine epidermal growth factor.

**Figure 3:**
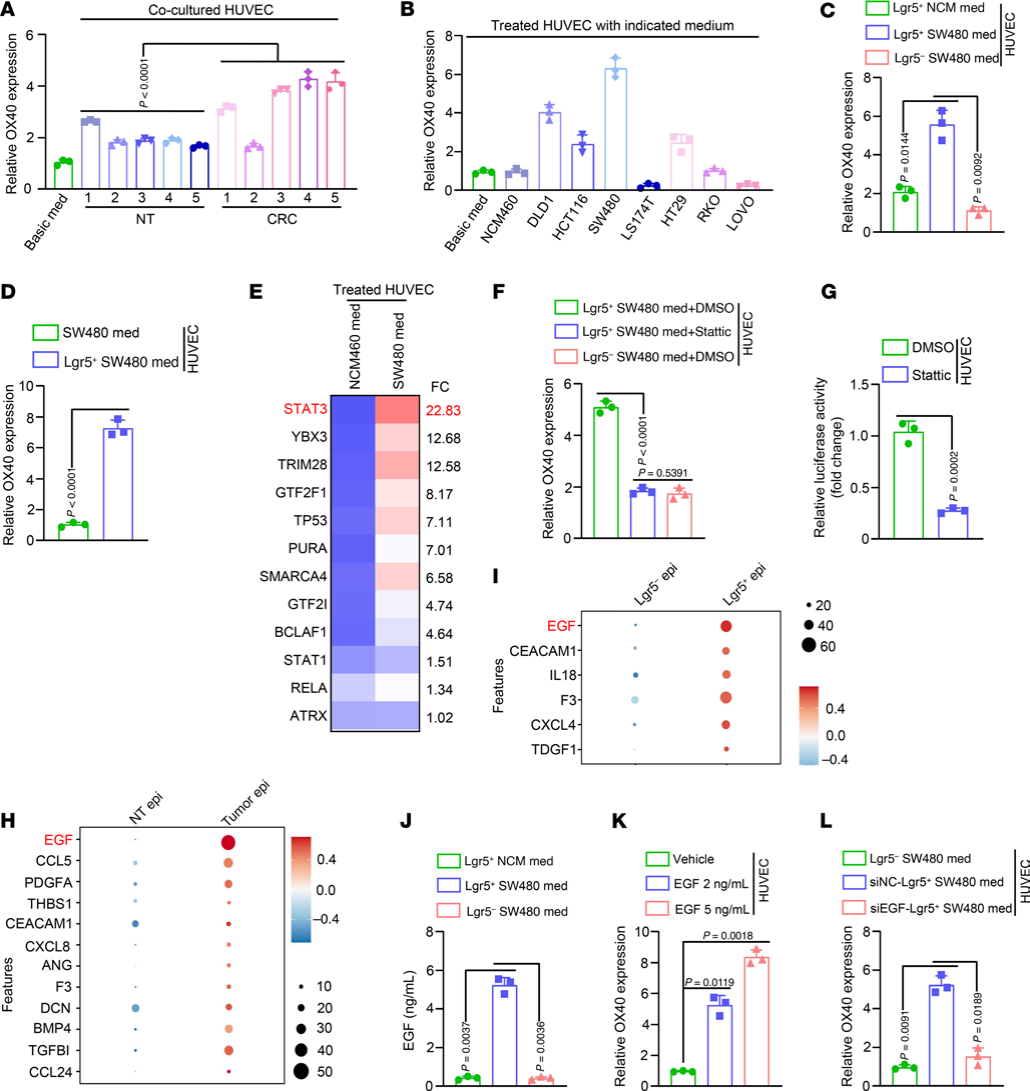
Cancer stem cell–derived EGF triggers OX40 expression in ECs. (**A**) Five pairs of shredded CRC and corresponding NT tissues suspended in basic medium were placed in the upper chamber of a 24-well Transwell system with polycarbonate filters. Thereafter, HUVECs were seeded in the bottom layer grown for 48 hours. Then, OX40 expression in HUVECs was measured by quantitative real-time PCR (qRT-PCR) (*n* = 3). (**B**–**D**) OX40 expression was measured in HUVECs treated with media derived from the indicated cells using qRT-PCR (*n* = 3). (**E**) HUVECs were treated with indicated media for 48 hours and then subjected to reverse chromatin immunoprecipitation analyses. The heatmap displays potential transcription factors that interact with OX40 promoter. (**F**) HUVECs were first treated with media derived from Lgr5^–^ and Lgr5^+^ SW480 cells and then exposed to DMSO or the STAT3 inhibitor Stattic (10 μM). OX40 expression was evaluated in HUVECs by qRT-PCR (*n* = 3). (**G**) Luciferase activity of the OX40 promoter in HUVECs treated with DMSO or the STAT3 inhibitor Stattic (*n* = 3). (**H** and **I**) Expression of 70 angiogenesis-associated cytokines was evaluated in tumor epithelial cells (Epi) (**H**) and Lgr5^+^ tumor epithelial cells (**I**) compared with their corresponding controls using scRNA-Seq data. (**J**) EGF levels were measured in media derived from the indicated cells using enzyme-linked immunosorbent assay (*n* = 3). (**K**) OX40 expression was evaluated in HUVECs treated with vehicle or exogenous EGF using qRT-PCR (*n* = 3). (**L**) First, scrambled NC (siNC) or siRNAs against EGF were transfected into Lgr5^+^ SW480 cells for 72 hours. Then, HUVECs were exposed to media derived from these cells. OX40 expression was evaluated in HUVECs using qRT-PCR (*n* = 3). One-way ANOVA (**A**, **C**, **F**, and **J**–**L**) or 2-tailed Student’s *t* test (**D** and **G**) was used for statistical analysis.

We next addressed why OX40 is highly expressed in tumor ECs. First, OX40 was highly expressed in HUVECs cocultured with CRC tissues compared with that in HUVECs cocultured with normal colon tissues (Figure 3A). Considering that CRC tissues are mainly composed of tumor cells, we measured OX40 expression in HUVECs treated with media from human CRC cell lines, including DLD1, HCT116, SW480, LS174T, HT29, RKO, LOVO, and colon epithelial NCM460 cells. We observed that HUVECs treated with media from most tumor cells harbored higher OX40 mRNA and protein levels (Figure 3B and Supplemental Figure 5A). These data suggest that tumor cells can upregulate OX40 expression in ECs.

Evidence has indicated that cancer stem cells (CSCs) undergo self-renewal and differentiation that contributes to tumor initiation, recurrence, and metastasis in tumors (22–24). CSCs are characterized by low differentiation and high malignancy and can promote rapid tumor progression via reprogramming of the microenvironment (25). Thus, we speculated that CSCs may affect OX40 expression in ECs of tumors. Supporting this hypothesis, OX40 gene expression was upregulated in HUVECs treated with media from leucine-rich repeat–containing GPCR5–positive (Lgr5^+^) SW480 cells compared with that in HUVECs treated with media from Lgr5^–^ SW480 cells (Figure 3C) or Lgr5^+^ NCM460 cells (Figure 3D). Considering that tumor stem cells promote OX40 expression in ECs at the transcriptional level, we analyzed TFs that may bind to the OX40 promoter using reverse ChIP. We identified STAT3 as a potential TF that regulates OX40 transcription because of its strong binding to the OX40 promoter in HUVECs treated with medium from SW480 cells (Figure 3E and Supplemental Table 1). Next, we obtained 3 lines of evidence to validate the transcriptional regulation of OX40 by STAT3: (a) phosphorylated and nuclear STAT3 were both upregulated in HUVECs treated with media from Lgr5^+^ SW480 cells compared with those treated with Lgr5^–^ counterparts (Supplemental Figure 5B); (b) treatment with the STAT3 inhibitor Stattic markedly impaired the upregulation of OX40 expression in HUVECs induced by Lgr5^+^ SW480 cell media (Figure 3F); (c) Stattic treatment inhibited OX40 promoter transcriptional activity in HUVECs (Figure 3G). Moreover, chromatin immunoprecipitation–PCR results consistently validated the transcriptional regulation of OX40 by STAT3 (Supplemental Figure 5, C and D). Overall, these data suggest that tumor stem cells activate OX40 transcription via STAT3 in ECs.

**Figure 4:**
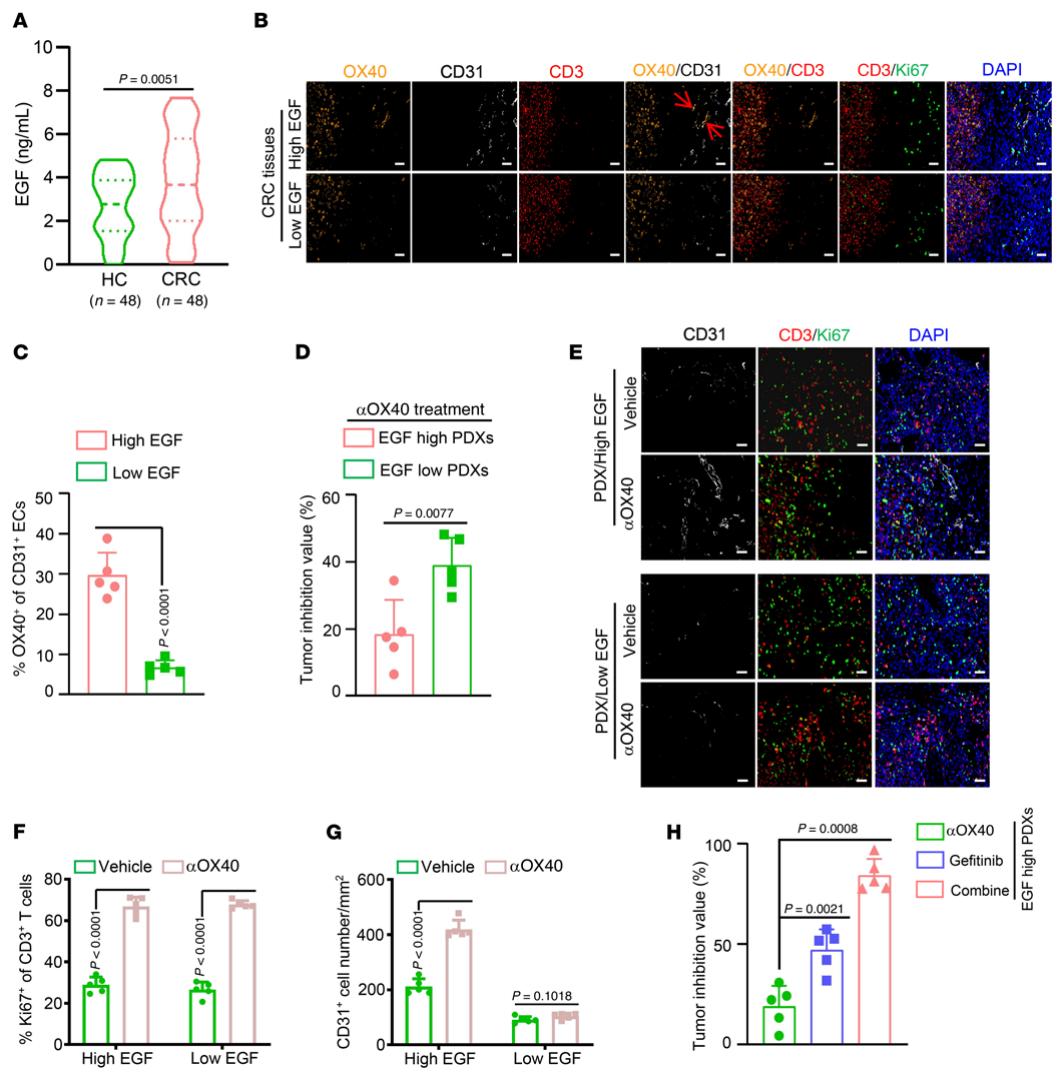
Serum EGF serves as a biomarker for predicting the efficacy of OX40 agonists. (**A**) EGF levels were measured in sera from patients with CRC and healthy control participants (HC) (*n* = 48). (**B** and **C**) The CRC patients were divided into 2 groups based on the median value of serum EGF levels: the EGF-low group and the EGF-high group. (**B**) Representative images of multicolor immunofluorescence using anti-OX40 (orange), anti-CD31 (white), anti-CD3 (red), and anti-Ki67 (green) antibodies in tumor tissues of CRC patients with high or low serum EGF levels (*n* = 5). Scale bars: 50 μm. (**C**) Statistical analysis of OX40^+^ EC percentages in tumor tissues of indicated CRC patients (*n* = 5). (**D**) Tumor inhibition values of anti-human αOX40 treatment (20 μg/mouse, i.p.) for PDX tumors derived from CRC patients with high or low serum EGF levels in BALB/c nude mice (*n* = 5). (**E**) Representative images of multicolor immunofluorescence using anti-CD3, anti-CD31, and anti-OX40 antibodies in PDX tumors from CRC patients with high or low serum EGF levels (*n* = 5). Scale bars: 50 μm. (**F** and **G**) Statistical analysis of Ki67^+^ T cell percentages (**F**) and vascular density (**G**; *n* = 5). (**H**) Tumor inhibition values of anti-human αOX40 (20 μg/mouse, i.p.), gefitinib (80 mg/kg, oral gavage), or their combination for PDX tumors from CRC patients with high serum EGF levels (*n* = 5). One-way ANOVA (**A**, **C**, **D**, **F**, and **G**) or 2-tailed Student’s *t* test (**H**) was used for statistical analysis.

Tumor stem cells reshape the TME via paracrine cytokines (26). We then screened for the expression of 70 angiogenesis-associated cytokines using the scRNA-Seq data. Epidermal growth factor (EGF) was the most highly upregulated cytokine in tumor epithelial cells and Lgr5^+^ tumor epithelial cells compared with their corresponding controls (Figure 3, H and I), implying that tumor stem cell–derived EGF may be a key factor in STAT3 activation and subsequent transcriptional activation of OX40 in ECs. In line with this hypothesis, EGF levels were markedly higher in the media from Lgr5^+^ SW480 cells than in the media from Lgr5^+^ NCM460 or Lgr5^–^ SW480 cells (Figure 3J). In addition, EGF/EGFR signaling was activated in HUVECs treated with media from Lgr5^+^ SW480 cells, as evidenced by the increased levels of phosphorylated JAK1 and Src (Supplemental Figure 5B). The epithelial cell subgroup from the tumor tissues displayed the highest level of EGF expression compared with the other cell subgroups (Supplemental Figure 5E). Furthermore, EGF treatment markedly upregulated OX40 expression in HUVECs (Figure 3K). siRNAs targeting the EGF gene remarkably disrupted the ability of Lgr5^+^ SW480 cells to upregulate OX40 gene expression in HUVECs (Figure 3L and Supplemental Figure 5F). Clinically, EGF levels were markedly higher in the sera and tumor tissues of patients with CRC than in their corresponding controls (Figure 4A and Supplemental Figure 5G). Importantly, patients with high serum EGF levels harbored more OX40^+^ ECs than those with low serum EGF levels (Figure 4, B and C). Moreover, the number of proliferative T cells in tumor tissues was not influenced by serum EGF (Figure 4B and Supplemental Figure 5H). In addition, we observed that EGF treatment barely impacted OX40 expression in T cells from tumor tissues (Supplemental Figure 5I), which might due to the negligible levels of EGF receptor (EGFR) gene expression in T cells compared with tumor ECs (Supplemental Figure 5J).

The above results have shown that tumor stem cells with high EGF levels would promote OX40 expression in ECs, inducing protumor effects. Thus, we examined the therapeutic efficacy of OX40 agonists in patient-derived xenografts (PDXs) with high or low serum levels. We observed more potent tumor-suppressing effects of αOX40 in EGF-low PDXs than in EGF-high PDXs, with average tumor inhibition values of 39.08% versus 18.45% (Figure 4D and Supplemental Figure 6A). Considering that there were no significant differences in the proliferative T cells of tumor tissues from patients with high or low serum EGF levels (Figure 4B and Supplemental Figure 5H), the reason for the poor efficacy of OX40 agonist in patients with high EGF levels might be attributed to the angiogenesis caused by higher OX40 expression in tumor ECs (Figure 4, B and C). Immunostaining analyses showed that OX40 agonist can equally activate T cell proliferation in PDX tumors from patients with either high or low serum EGF levels (Figure 4, E and F); however, the OX40 agonist further induced more angiogenesis in PDX tumors from patients with high serum EGF levels than in their low-EGF counterparts (Figure 4, E and G). The above data indicate that the EGF level is an important indicator of OX40 agonist immunotherapy application, and patients with low EGF levels would be more responsive to OX40 agonist therapy.

Considering the crucial role of EGF signaling in OX40 protumor effects in tumor ECs, we hypothesized that EGF signaling inhibitors would disrupt the adverse protumor effects of OX40 in ECs. To this end, we tested the effects of a combination of gefitinib, an EGF signaling inhibitor, and αOX40 treatment in EGF-high PDXs. We found that αOX40 alone had no significant antitumor effects (Supplemental Figure 6B); however, the drug combination exhibited a powerful antitumor effect, with an average tumor inhibition value of 84.34% for 5 PDXs (Figure 4H), which was far higher than that of αOX40 alone (18.45%). Cumulatively, these data suggest that EGF signaling inhibitors attenuate angiogenesis, thus improving the antitumor therapeutic effects of OX40 agonists.

### OX40 signal promotes endothelial-mesenchymal transition by promoting YAP nuclear translocation.

After elucidating the reasons for the high expression of OX40 in tumor ECs, we attempted to explore the mechanism by which OX40 signal affects ECs functions. We first examined whether OX40 activation affects the PI3K/AKT or NF-κB signaling pathways in ECs, which are canonical downstream pathways of OX40 signaling in T cells (27). Unlike in T cells (Supplemental Figure 7A), OX40 activation did not impact the activities of PI3K/AKT or NF-κB signaling pathways in ECs (Supplemental Figure 7B). This suggests that the downstream pathways of OX40 signaling in ECs are distinct from T cells.

**Figure 5:**
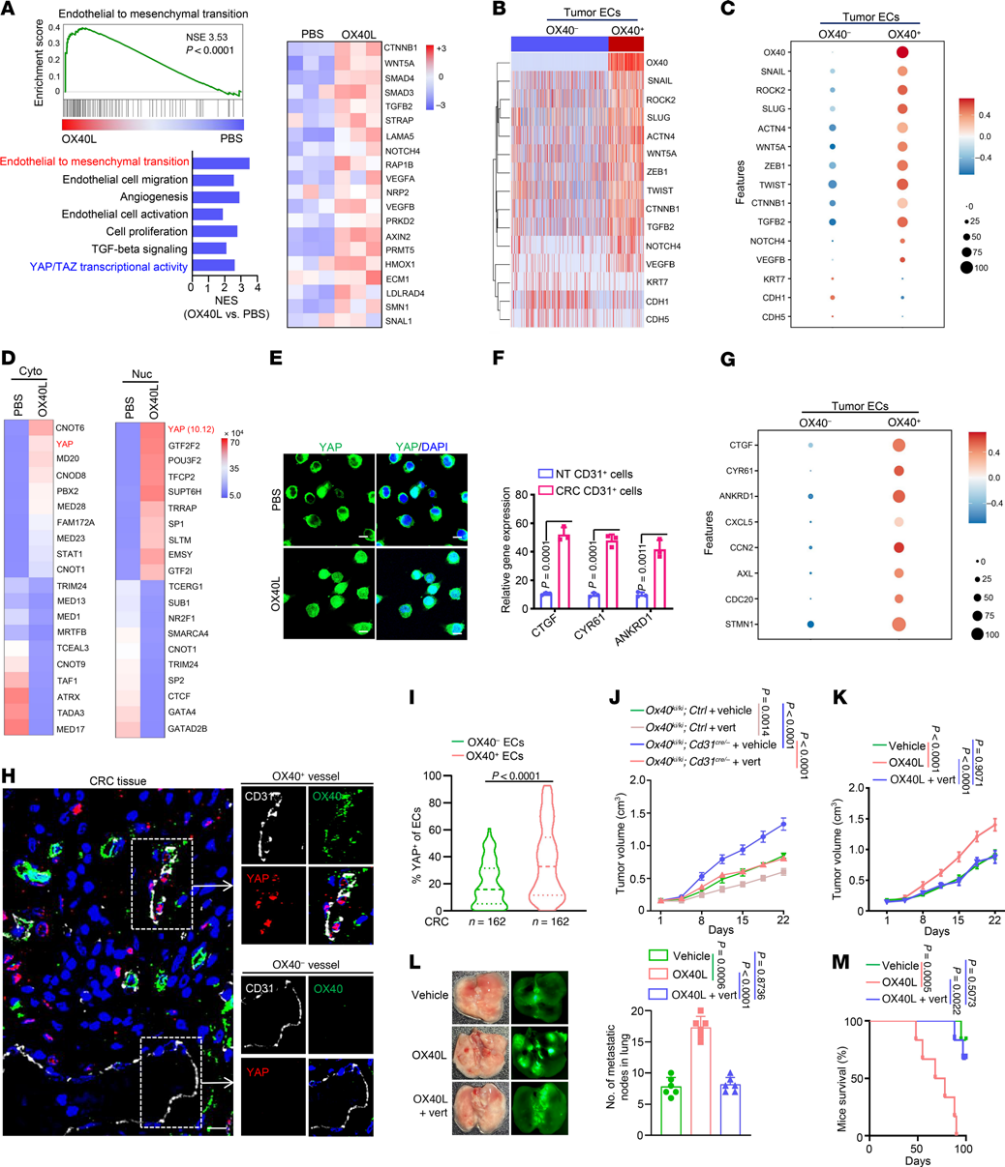
OX40 signal exerts protumor effects by promoting YAP nuclear translocation. (**A**) Tumor cell medium–stimulated HUVECs were treated with PBS or OX40L protein (100 ng/mL) for 48 hours and then subjected to transcriptome sequencing. Enriched pathways were pooled using differentially expressed genes after OX40L treatment (left). The heatmap displays the expression of genes associated with endothelial-mesenchymal transition (right). (**B** and **C**) Genes encoding mesenchymal markers and adhesive molecules were evaluated in OX40^–^ or OX40^+^ tumor ECs using scRNA-Seq data. (**D**) PBS- or OX40L-treated HUVECs were fractionated into cytoplasmic (Cyto) and nuclear (Nuc) fractions and subjected to protein mass spectrometry. (**E**) PBS- or OX40L-treated HUVECs were immunostained with YAP antibody. Scale bars: 5 μm. (**F** and **G**) Expression of YAP downstream genes was measured in sorted CD31^+^ cells from CRC and NT tissues by qRT-PCR (**F**, *n* = 3) and in OX40^–^ and OX40^+^ ECs from CRC tissues using scRNA-Seq data (**G**, *n* = 5). (**H**) Representative images of CRC tissues immunostained using the indicated antibodies. The white boxes indicate OX40^+^ or OX40^–^ ECs (*n* = 162). Scale bar: 5 μm. (**I**) YAP^+^ percentages in OX40^–^ ECs and OX40^+^ ECs from tumor tissues (*n* = 162). (**J**) Subcutaneous tumors were established in mice with conditional knockin of OX40 in ECs or in control mice. The mice were treated with verteporfin for 3 weeks (*n* = 10). (**K**–**M**) Subcutaneous tumor and pulmonary metastasis models were established using MC38 cells in BALB/c nude mice. The mice were treated with OX40L or a combination of mouse OX40L protein and verteporfin. (**K**) Tumor volume (*n* = 8). (**L** and **M**) Metastatic nodules in the lung (**L**) and mouse survival (**M**) (*n* = 6). Two-tailed Student’s *t* test (**F** and **I**), 2-way ANOVA (**J** and **K**), 1-way ANOVA (**L**), or log-rank test (**M**) was used for statistical analysis.

Next, we identified the signaling pathways responsible for the tumor-promoting effects of OX40 in ECs using RNA sequencing. The Kyoto Encyclopedia of Genes and Genomes (KEGG) enrichment analysis revealed that the differentially expressed genes in OX40L-treated ECs were primarily associated with endothelial-mesenchymal transition (EndMT), EC migration, angiogenesis, and Yes 1–associated protein/TAZ (YAP/TAZ) transcriptional activity (Figure 5A). The EndMT-associated genes CTNNB1, WNT5A, SMAD3/4, TGFB2, VEGFA, and SNAIL1 were upregulated in OX40L-treated ECs (Figure 5A). The mesenchymalization of ECs is a critical factor in promoting hematogenous tumor metastasis and is closely related to tumor angiogenesis (28). We analyzed the expression of EndMT-associated genes in OX40^–^ and OX40^+^ tumor EC subpopulations using scRNA-Seq data. The mesenchymal markers SNAIL, SLUG, TWIST, and ZEB1 were upregulated in OX40^+^ tumor ECs, whereas the adhesion molecules CDH1 (encoding E-cadherin) and CDH5 (encoding VE-cadherin) were downregulated (Figure 5, B and C). Phenologically, ECs lost their pebble-like morphological features and transformed into spindle-like structures, extending pseudopodia, and exhibiting a mesenchymal phenotype after stimulation with OX40L (Supplemental Figure 8A). The endothelial-mesenchymal markers α-SMA and vimentin were markedly upregulated and the endothelial cell–specific cell-cell adhesion molecule VE-cadherin was reduced in OX40L-treated ECs (Supplemental Figure 8B). Thus, the results suggest that OX40 activation triggers EndMT, inducing alterations in the phenotype of ECs.

EndMT initiation always results from the induction of TFs that alter gene expression to promote the loss of cell-cell adhesion, leading to a shift in cytoskeletal dynamics and a change from epithelial morphology and physiology to the mesenchymal phenotype (29, 30). Given the crucial role of nuclear translocation in the functioning of TFs, we identified TFs that undergo nuclear translocation after OX40 activation in ECs. Among the potential transcriptional regulators, YAP was selected for further validation because of its significant nuclear translocation and enriched YAP/TAZ transcriptional activity according to KEGG analysis (Figure 5A) after OX40L treatment and its crucial role in tumor progression (Figure 5D and Supplemental Table 2). We confirmed the enhanced YAP nuclear translocation (Figure 5E and Supplemental Figure 9A) and transcriptional activity (Supplemental Figure 9B) following OX40 activation. Moreover, the YAP downstream genes CTGF, CYR61, and ANKRD1 were highly expressed in tumor ECs (CD31^+^ cells) compared with their NT counterparts (Figure 5F). Similarly, these genes were highly expressed in the OX40^+^ tumor EC subpopulations (Figure 5G). Moreover, we observed more YAP nuclear localization in OX40^+^ vessels than in OX40^–^ vessels in tumor tissues (Figure 5H). Statistically, YAP was markedly more highly expressed in OX40^+^ ECs than in OX40^–^ ECs (Figure 5I). The results revealed that OX40 signaling in tumor ECs sustains YAP protein stability and promotes its nuclear translocation.

We then clarified the effects of YAP nuclear translocation on EC functions regulated by OX40 signaling. Verteporfin, an inhibitor of YAP–transcriptional enhanced associated domain (TEAD) interactions (31), effectively weakened cell migratory ability (Supplemental Figure 9C), tube-forming capacity (Supplemental Figure 9D), and tumor cell transendothelial migratory ability (Supplemental Figure 9E) promoted by OX40 activation. Moreover, verteporfin reversed the protumor effects of conditional OX40 overexpression in ECs (Figure 5J) or OX40L treatment (Figure 5, K–M). Collectively, these data suggest that OX40 signal exerts protumor effects in ECs by promoting nuclear translocation of YAP and subsequent transcriptional regulatory activity. Interestingly, OX40 activation did not impact the YAP abundance and phosphorylation in T cells (Supplemental Figure 7A), implying distinct downstream pathways activated by OX40 signaling in T cells and ECs.

### OX40 signal decreases YAP phosphorylation by regulating S1P-YAP interaction.

Next, we analyzed the mechanisms by which OX40 activation triggers YAP nuclear translocation. Notably, the YAP mRNA levels were barely affected (Supplemental Figure 10A), whereas a significant upregulation in protein abundance was observed with OX40 activation (Supplemental Figure 10B), which was consistent with the protein mass spectrometry results shown in Figure 5D. These results revealed that OX40 signal may regulate YAP protein stability. Indeed, we observed a significant increase in YAP protein stability and attenuated YAP ubiquitination after OX40 activation (Supplemental Figure 10, C and D). YAP undergoes degradation in a phosphorylation-dependent manner (32–34). The E3 ubiquitin ligase β-TrCP is involved in the phosphorylation-dependent degradation of YAP (32). Indeed, we observed a weakened interaction between β-TrCP and YAP following OX40 activation (Supplemental Figure 10E). These results suggest that OX40 activation inhibits YAP phosphorylation and subsequent β-TrCP–mediated YAP protein ubiquitination.

**Figure 6:**
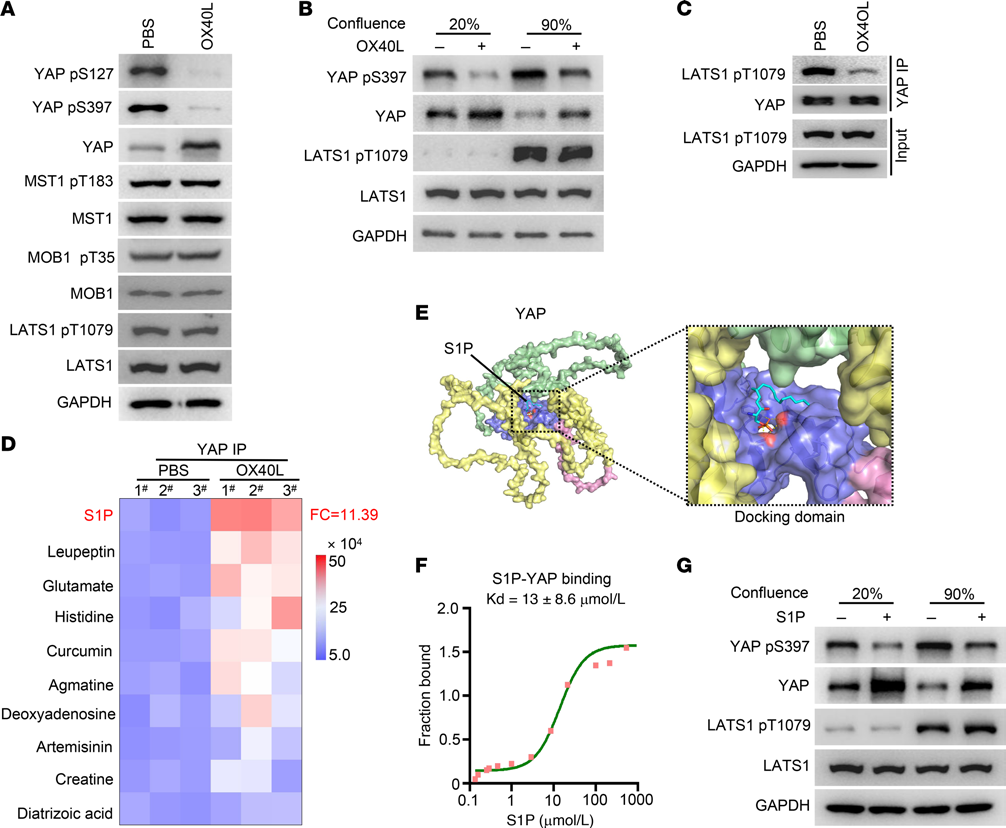
OX40 signal impacts YAP protein stability through modulating S1P-YAP interaction. (**A**) HUVECs were treated with PBS or human OX40L protein (100 ng/mL) for 48 hours. The cell lysates were immunoblotted using the indicated antibodies. (**B**) HUVECs were cultured at 20% or 90% confluence. The cells were treated with PBS or human OX40L protein (100 ng/mL) for 48 hours and subjected to immunoblotting using the indicated antibodies. (**C**) HUVECs were treated with PBS or human OX40L (100 ng/mL) for 48 hours. Cell lysates were immunoprecipitated using an anti-YAP antibody, followed by blotting with the indicated antibodies. (**D**) HUVECs were treated with PBS or human OX40L protein (100 ng/mL) for 48 hours. Cell lysates were immunoprecipitated using an anti-YAP antibody. Metabolites in the immunocomplexes were extracted. The quantitative abundance of metabolites was measured using a trace-level metabolite detection method based on liquid chromatography–mass spectrometry (LC-MS) (*n* = 3). (**E**) Docking model of S1P and YAP. The full-length YAP structure was predicted using AlphaFold. Surface presentation of the YAP complex with S1P (cyan) bound to its central cavity. (**F**) Analysis of S1P binding to the purified YAP protein using the in vitro microscale thermophoresis binding assay. (**G**) HUVECs were cultured at 20% or 90% confluence. The cells were then treated with PBS or S1P (10 μM) for 48 hours and subjected to immunoblotting using the indicated antibodies.

YAP is an important downstream effector molecule of the Hippo pathway and is phosphorylated by the MST1/2 and large tumor suppressor kinases 1 and 2 (LATS1/2) cascades (34, 35). Therefore, we evaluated the effect of OX40 activation on the Hippo pathway. First, OX40 activation inhibited YAP phosphorylation and increased YAP protein abundance (Figure 6A), whereas the protein abundance and phosphorylation of several crucial kinases of the Hippo pathway, including MST1, MOB1, and LATS1, were barely affected (Figure 6A). Moreover, OX40 activation regulated the abundance and phosphorylation of YAP protein, regardless of Hippo pathway activation (Figure 6B). Interestingly, we observed that OX40 activation markedly inhibited the interaction between YAP and phosphorylated LATS1 (p-LATS1) (Figure 6C). These results suggest that OX40 activation inhibits YAP protein phosphorylation mainly by impacting the interaction of YAP and p-LATS1, rather than by affecting the phosphorylation of several crucial kinases of the Hippo pathway.

Next, we aimed to answer how OX40 activation impacts the interaction of YAP and p-LATS1. Small-molecule metabolites can directly bind to target proteins and act as functional ligands to regulate protein-protein interactions and influence protein activity (36–38). This led us to hypothesize that metabolites may participate in the regulation of the interaction between YAP and p-LATS kinases by binding to YAP. Thus, we identified small-molecule metabolites that directly bind to YAP using the trace-level metabolite quantitation. The results demonstrated that OX40 activation promoted YAP protein binding with abundant sphingosine 1-phosphate (S1P), which was the most markedly upregulated metabolite (Figure 6D and Supplemental Table 3). Molecular docking analysis further showed that S1P preferentially docked in the central cavity at an interface formed by the TEAD-binding domain, WW structural domain, SH3-binding domain, and PDZ-binding domain (Figure 6E). Biochemically, the in vitro microscale thermophoresis binding assay presented a direct interaction between purified YAP protein and S1P with a dissociation constant of 13 μmol/L (Figure 6F). Previous studies have shown that S1P activates downstream signaling pathways via interacting with S1P receptors (S1PR1–5) (39). However, S1PR1–5 silencing had a negligible impact on YAP phosphorylation and total protein abundance (Supplemental Figure 11, A and B). Thus, S1P exerts biological functions by directly binding to YAP in ECs, rather than activating signals through interacting with S1PRs.

**Figure 7:**
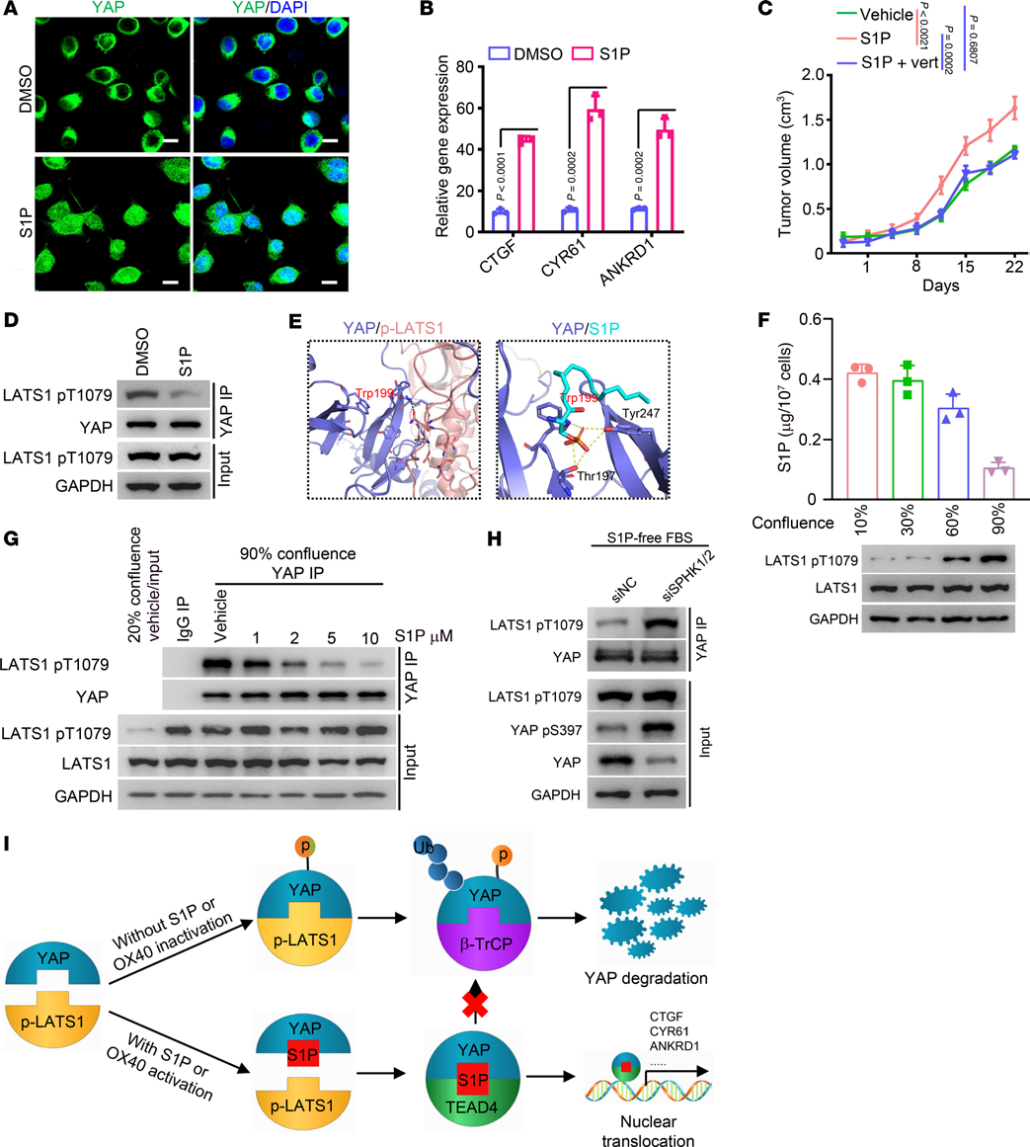
S1P disrupts the interaction between YAP and the p-LATS1 kinase. (**A**) HUVECs treated with DMSO or S1P (10 μM) were immunostained with an anti-YAP antibody. Scale bars: 5 μm. (**B**) Expression of YAP downstream genes was evaluated in HUVECs treated with DMSO or S1P using qRT-PCR (*n* = 3). (**C**) Subcutaneous tumor models were established using MC38 cells in BALB/c nude mice. The mice were treated with S1P (5 mg/kg, i.p.) or a combination of S1P and verteporfin (*n* = 8). (**D**) DMSO- or S1P-treated HUVECs were immunoprecipitated using an anti-YAP antibody followed by blotting with the indicated antibodies. (**E**) Docking models of YAP–p-LATS1 and YAP-S1P. (**F**) HUVECs were grown to 10%, 30%, 60%, or 90% confluence. Cell lysates were immunoprecipitated using an anti-YAP antibody. p-LATS1 expression was measured using Western blot analysis in total cell lysates. Metabolites in the immunocomplex were extracted. The quantitative abundance of S1P was measured using an LC-MS–based trace-level metabolite detection method (*n* = 3). (**G**) HUVECs were grown to 90% confluence. Then, the cells were treated with DMSO or S1P at the indicated concentrations and subjected to immunoprecipitation using IgG or anti-YAP antibodies. Cells at 20% confluence were used as a control. (**H**) HUVECs were cultured with S1P-free fetal bovine serum. The cells were transfected with scrambled siRNA (siNC) or mixed siRNAs against SPHK1 and SPHK2. Cell lysates were immunoprecipitated with an anti-YAP antibody and blotted with the indicated antibodies. (**I**) A diagram summarizing the proposed model in which OX40 activation or accumulated S1P disrupts the YAP and p-LATS1 interaction, leading to increased YAP stability, augmented YAP-TEAD4 interaction, and, ultimately, transactive capacity. Two-tailed Student’s *t* test (**B**) or 2-way ANOVA (**C**) was used for statistical analysis.

Similarly to OX40L treatment, exogenous supplementation with S1P regulated the abundance and phosphorylation of YAP regardless of Hippo pathway activation (Figure 6G). Moreover, S1P treatment increased YAP stability (Supplemental Figure 12A), attenuated YAP ubiquitination (Supplemental Figure 12B), and augmented YAP nuclear translocation (Figure 7A) and subsequent transcriptional activity (Figure 7B). Importantly, verteporfin markedly disrupted the tumor-promoting role in vivo (Figure 7C), and the migratory ability (Supplemental Figure 12C), tube-forming capacity (Supplemental Figure 12D), and tumor cell transendothelial migratory ability of ECs (Supplemental Figure 12E) induced by exogenous S1P supplementation. More importantly, S1P inhibited the YAP–p-LATS1 interaction (Figure 7D). Trp199, one of the predominant amino acids of the YAP protein interacting with S1P, occupies the partial binding sites of YAP and p-LATS1, predicting that S1P may competitively bind to YAP with p-LATS1 (Figure 7E). Moreover, the increased levels of p-LATS1 inhibited the binding of S1P to YAP (Figure 7F). By contrast, S1P supplementation disrupted the interaction between p-LATS1 and YAP in a dose-dependent manner (Figure 7G). Additionally, knocking down the S1P synthetases SPHK1 and SPHK2 together markedly strengthened the interaction between p-LATS1 and YAP, resulting in an increase in YAP phosphorylation and reduced YAP protein abundance (Figure 7H). Finally, we performed computational structural prediction of the YAP–p-LATS1 interaction under the premise of S1P participation. S1P participation displayed a higher docking score, predicting a weakened affinity intensity between YAP and p-LATS1 (Supplemental Figure 13A). Therefore, these data suggest that S1P computationally, biochemically, and mechanistically binds to YAP, and competitively disrupts the interaction between YAP and the p-LATS1 kinase, thus promoting the stability of YAP protein and nuclear translocation.

We set out to investigate the effects of S1P on nuclear YAP. OX40 activation increased the binding of S1P to nuclear YAP (Supplemental Figure 13B). Moreover, either OX40 activation or exogenous S1P supplementation enhanced the interaction between YAP and TEAD4 (Supplemental Figure 13, C and D), indicating that S1P not only maintains the stability of YAP but also contributes to the YAP-TEAD4 interaction to strengthen YAP transcriptional activity. The schematic diagram in Figure 7I summarizes that S1P directly binds to YAP, disrupts the interaction between YAP and p-LATS1, and enhances the binding of YAP to TEAD4, thereby promoting YAP nuclear translocation and transcriptional activity.

### Activated OX40 causes S1P accumulation by interacting with the Spns2 transporter in ECs.

**Figure 8:**
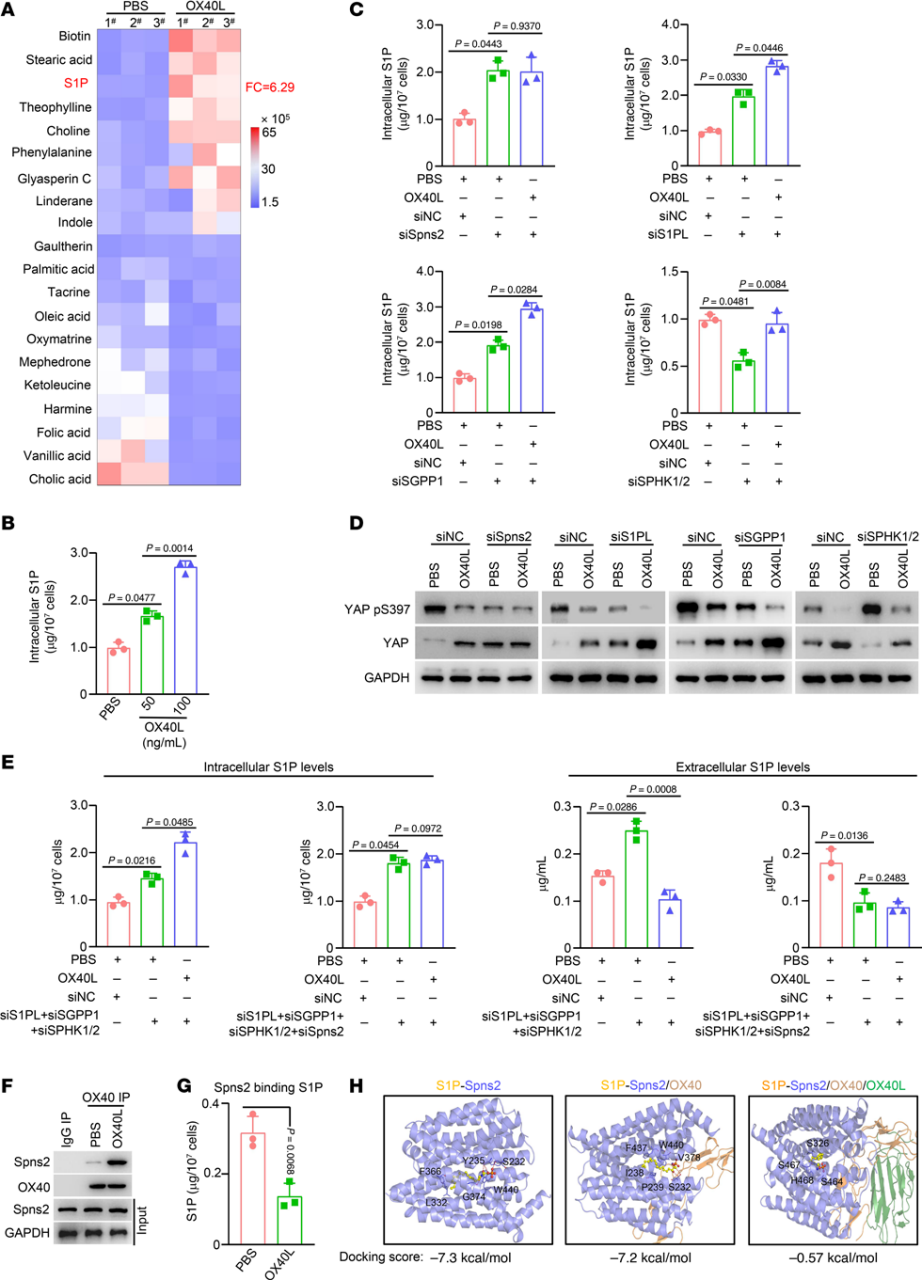
OX40 signal induces S1P accumulation via interaction with Spns2. (**A**) Tumor cell medium–stimulated HUVECs were treated with PBS or OX40L protein (100 ng/mL) for 48 hours. Cells were harvested and metabolites were extracted. Metabolite abundance was determined using an untargeted metabolomic test (*n* = 3). Heatmaps displayed the top 20 differential metabolites (10 upregulated and 10 downregulated). (**B**) Quantitative abundance of S1P was measured in HUVECs treated with PBS or OX40L protein at the indicated concentrations for 48 hours (*n* = 3). (**C**) HUVECs were treated with vehicle, OX40L protein, or OX40L plus siRNAs against Spns2, S1PL, SGPP1, or SPHK1/2. The quantitative abundance of intracellular S1P was measured (*n* = 3). (**D**) HUVECs were treated with vehicle, human OX40L protein (100 ng/mL), or OX40L plus siRNAs against Spns2, S1PL, SGPP1, or SPHK1/2. The cells were then subjected to immunoblotting using the indicated antibodies. (**E**) HUVECs were treated with vehicle, human OX40L protein (100 ng/mL), OX40L plus multiple siRNAs against S1PL, SGPP1, and SPHK1/2 together, or OX40L plus multiple siRNAs against Spns2, S1PL, SGPP1, and SPHK1/2 together. The quantitative abundances of intracellular (left) and extracellular (right) S1P were measured (*n* = 3). (**F**) HUVECs were treated with vehicle or human OX40L protein (100 ng/mL) for 48 hours. Cell lysates were immunoprecipitated using an anti-OX40 antibody, followed by blotting with the indicated antibodies. (**G**) HUVECs were treated with vehicle or OX40L protein for 48 hours. Cell lysates were immunoprecipitated using an anti-Spns2 antibody. Metabolites in the immunocomplex were extracted. The quantitative abundance of S1P was measured using an LC-MS–based trace-level metabolite detection method (*n* = 3). (**H**) Different docking models of S1P and Spns2. One-way ANOVA (**B**, **C**, and **E**) or 2-tailed Student’s *t* test (**G**) was used for statistical analysis.

Next, we investigated whether OX40 signaling influences S1P levels in ECs. As expected, OX40 activation induced S1P accumulation in ECs (Figure 8, A and B, and Supplemental Table 4). In addition, EGF exposure resulted in elevated S1P abundance in HUVECs (Supplemental Figure 14A); siRNAs targeting either EGFR or STAT3 genes remarkably abrogated the ability of exogenous EGF to upregulate S1P levels (Supplemental Figure 14, A and B), indicating that EGF/STAT3/OX40 signaling could lead to S1P accumulation.

We then unveiled the molecular mechanisms by which OX40 activation led to S1P accumulation. S1P is a biologically active lipid, and its synthesis, degradation, and secretion are dynamically regulated by various enzymes and transporters, including sphingosine kinase (SPHK1/2), S1P phosphorylase (SGPP1), S1P lyase (S1PL), and the transporter Spns lysolipid transporter 2 (Spns2) (40).

First, Spns2 knockdown abolished S1P accumulation in ECs induced by OX40 activation, whereas S1PL, SGPP1, or SPHK1/2 knockdown did not affect the increase in S1P abundance promoted by OX40 signal (Figure 8C). We further evaluated the impact of these genes on YAP protein. Results demonstrated that Spns2 knockdown, rather than S1PL, SGPP1, or SPHK1/2 knockdown, abolished the alterations in YAP phosphorylation and total protein abundance induced by OX40 activation (Figure 8D and Supplemental Figure 14C). The above data suggest that the Spns2 transporter may be involved in the regulation of OX40 signaling in S1P abundance and subsequent YAP stability in ECs. Spns2 is a membrane protein that primarily is responsible for the active transport of S1P from the inside to the outside of cells. To consolidate the roles of Spns2 in OX40 functioning in ECs, we first assessed the alterations in intracellular and extracellular S1P levels of HUVECs by simultaneously knocking down genes associated with S1P synthesis and degradation, including SPHK1/2, SGPP1, and S1PL. As expected, upon knockdown of these genes together, OX40 activation still caused changes in intracellular or extracellular S1P levels (Figure 8E). However, further silencing of Spns2 transporter together with SPHK1/2, SGPP1, and S1PL knockdown did not influence the alteration of intracellular or extracellular S1P levels induced by OX40 activation (Figure 8E). These data strongly imply that Spns2 transporter plays a decisive role in S1P accumulation regulated by OX40 activation in ECs. Coimmunoprecipitation results demonstrated that OX40 physically interacted with Spns2 at a marginal level. However, OX40 activated by OX40L treatment displayed a strong interaction with Spns2 (Figure 8F). The binding of S1P to the Spns2 transporter is a key process in S1P efflux (41). We observed that OX40 activation inhibited S1P binding to Spns2 (Figure 8G). We further conducted a computational structural prediction to assess the influence of OX40 activation on the Spns2-S1P interaction. Indeed, only OX40 had a marginal impact on Spns2-S1P affinity intensity; however, further involvement of OX40L to activate OX40 markedly inhibited Spns2-S1P affinity intensity (Figure 8H). These results demonstrate that activated OX40 disrupted the interaction of Spns2 and S1P, thereby inhibiting S1P export. We further compared S1P abundance and the expression of S1P metabolism–associated genes in T cells and ECs sorted from CRC tissues. The expression levels of S1PL, SGPP1, SPHK1/2, and Spns2 in T cells were markedly lower than those in ECs (Supplemental Figure 15A). Similarly, S1P levels were dramatically lower in T cells (Supplemental Figure 15B). This may explain why OX40 activation had no impact on YAP protein abundance and phosphorylation in T cells.

### Monocytic OX40L may be an upstream signal for OX40 activation in tumor ECs.

As a costimulatory factor, activation of OX40 requires the binding to OX40L (42). Our next goal was to identify the upstream OX40L source that activates OX40 in colon cancer. First, we observed that free OX40L levels in the serum of patients with CRC were almost equivalent to those in healthy controls (Supplemental Figure 16A). Then we analyzed OX40L expression in peripheral blood cells. OX40L is primarily expressed in antigen-presenting cells including B cells, dendritic cells (DCs), and monocytes (42). Thus, we analyzed the OX40L^+^ percentages in these cells. In contrast to B cells and DCs, the percentage of OX40L^+^ monocytes was markedly higher in patients with CRC than in healthy controls (Supplemental Figure 16B). Moreover, the absolute numbers of OX40L^+^ monocytes were dramatically higher than those of OX40L^+^ B cells or OX40L^+^ DCs in the peripheral blood of patients with CRC (Supplemental Figure 16C).

Next, we determined whether monocytic OX40L induces OX40 activation in ECs, thereby regulating EC function. We cocultured HUVECs with monocytes isolated from the peripheral blood of healthy controls and patients with CRC and evaluated YAP abundance and phosphorylation. As expected, HUVECs cocultured with most CRC patient–derived monocytes displayed higher YAP abundance and lower phosphorylation than HUVECs cocultured with healthy control–derived monocytes (Supplemental Figure 17A). Moreover, HUVECs cocultured with CRC patient–derived monocytes exhibited enhanced migratory ability, tube-forming capacity, and tumor cell transendothelial migratory ability (Supplemental Figure 17, B and C) and increased expression levels of YAP downstream genes CTGF, CYR61, and ANKRD1 (Supplemental Figure 17D). These data suggest that monocytic OX40L can activate OX40 signaling in the ECs, thereby regulating their functions.

### Combination of YAP-TEAD inhibitor and OX40 agonist induces tumor regression.

**Figure 9:**
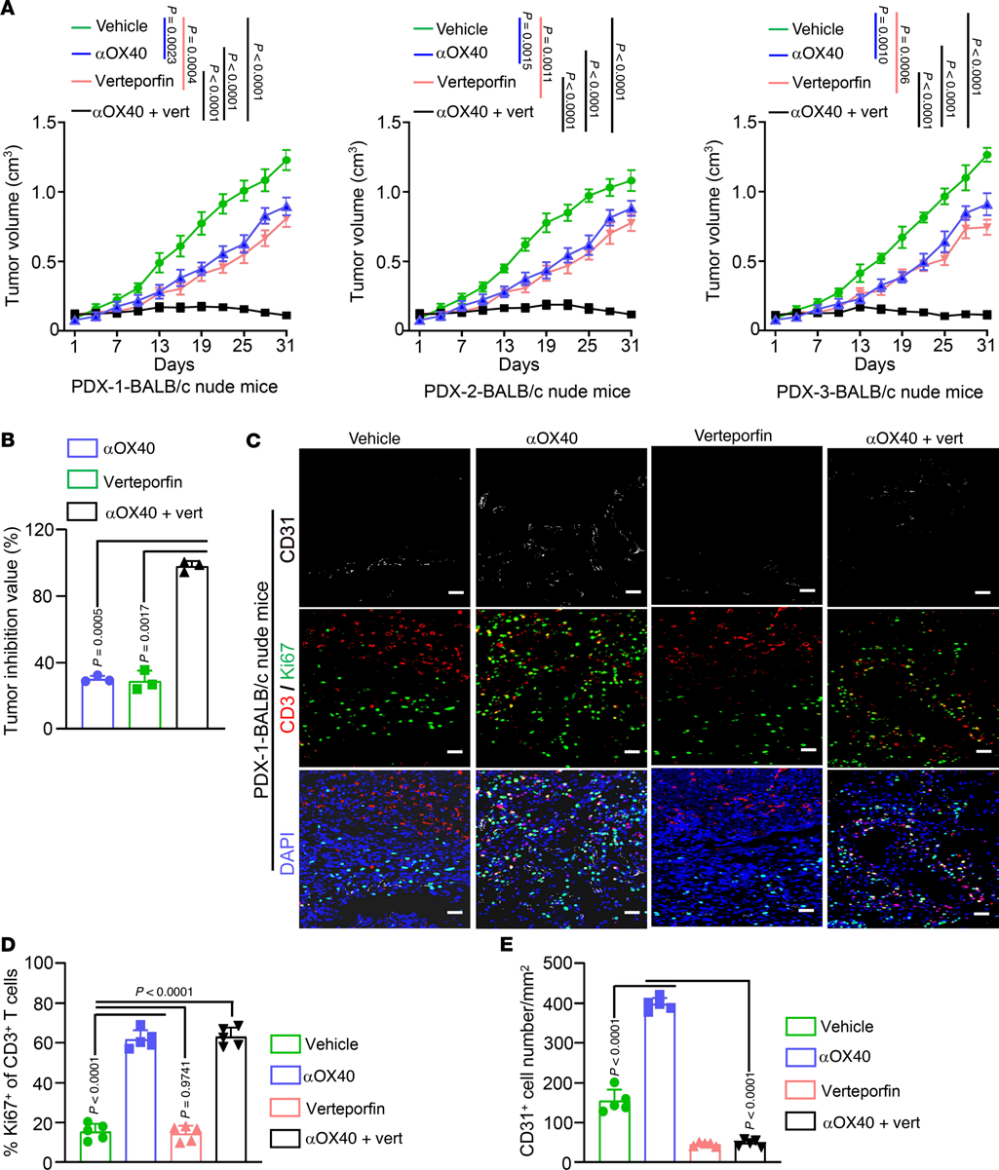
The combination of verteporfin and OX40 agonist synergistically leads to tumor repression in PDX tumors. (**A** and **B**) Subcutaneous xenograft tumors established using 3 CRC patient–derived xenografts (PDXs) in BALB/c nude mice were treated with anti-human αOX40 (20 μg/mouse, i.p.), verteporfin (60 mg/kg, i.p.), or a combination of both (**A**; *n* = 6 or 7 mice per cohort). Tumor inhibition values for αOX40, verteporfin, and drug combinations were calculated and compared (**B**). (**C**–**E**) Subcutaneous tumors generated from CRC PDX-1 were immunostained using anti-CD31 (white), anti-CD3 (red), and anti-Ki67 (green) antibodies. (**C**) Representative images of multicolor immunofluorescence (*n* = 5). Scale bars: 50 μm. (**D** and **E**) Quantification of Ki67^+^ T cell percentages (**D**) and vascular density (**E**) (*n* = 5). Two-way ANOVA (**A**) or 1-way ANOVA (**B**, **D**, and **E**) was used for statistical analysis.

Based on our results, 2 independent regulatory pathways for OX40 signaling were identified. In T cells, activation of OX40 signaling boosts antitumor effects by enhancing immune responses, whereas in tumor ECs, the activation of OX40 signaling induces EndMT by regulating YAP nuclear translocation and promoting angiogenesis, thereby exerting protumor effects. Thus, enhancing the T cell antitumor response together with disrupting the protumor effects of OX40 signaling in ECs may contribute to improving the therapeutic effect of OX40 agonists. To achieve this, we used verteporfin, a potent inhibitor of the YAP-TEAD interaction, which has shown remarkable anti-angiogenic effects in vitro and in vivo (43, 44). We implemented a therapeutic strategy using CRC PDX models to evaluate the effects of OX40 agonists alone or in combination with verteporfin. Either drug alone decelerated tumor growth (Figure 9A). However, the combination of αOX40 and verteporfin synergistically restricted tumor growth and induced tumor regression, with an average tumor inhibition value of 97.88% (Figure 9, A and B). In contrast to the data in Figure 1, treatment with αOX40 inhibited PDX tumor growth, although the same T cell–immunodeficient BALB/c nude mice were used. We inferred that human donor tissue–derived T cells surviving in PDX tissues (45–48) may be activated by OX40 agonists. As anticipated, increased proliferation of T cells (Figure 9, C and D) and augmented production of intracellular cytokines including granzyme B, perforin, and IFN-γ in T cells (Supplemental Figure 18) from PDX tumors were observed after αOX40 treatment. In addition, verteporfin treatment inhibited angiogenesis (Figure 9, C and E), and the drug combination concurrently reinforced the T cell antitumor response and weakened vascular density (Figure 9, C–E, and Supplemental Figure 18).

**Figure 10:**
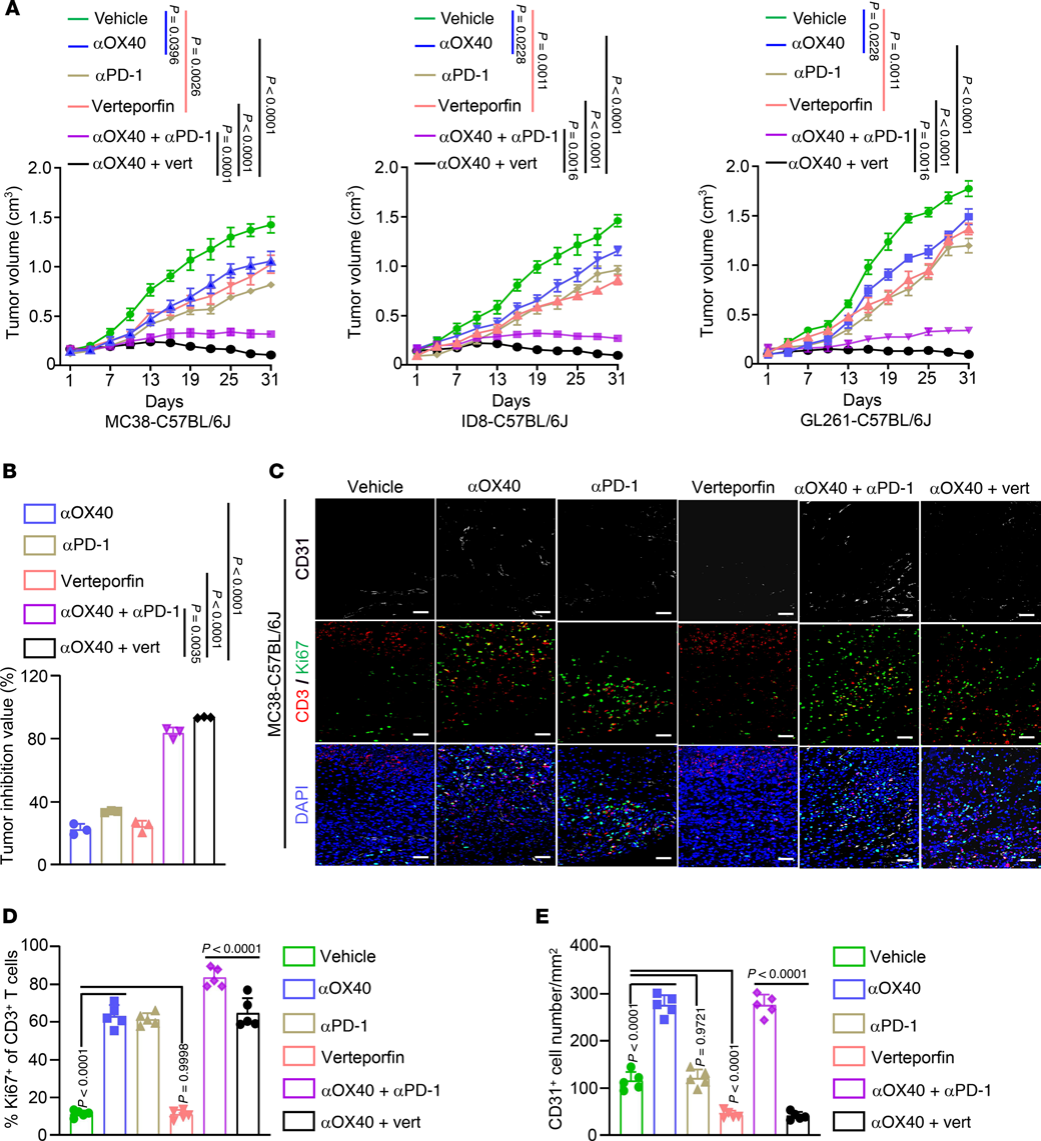
The synergistic effects of the OX40 agonist and verteporfin were superior to the OX40 agonist and PD-1 antibody combination. (**A** and **B**) Subcutaneous tumors established using MC38, ID8, or GL261 cells in C57BL/6J mice were treated with anti-mouse αOX40 (100 μg/mouse, i.p.), anti–mouse PD-1 antibody (αPD-1; 200 μg/mouse, i.p.), verteporfin (60 mg/kg, i.p.), αOX40 plus αPD-1, or αOX40 plus verteporfin (**A**; *n* = 8 mice per cohort). Tumor inhibition values for αOX40, αPD-1, verteporfin, and drug combinations were calculated and compared (**B**). (**C**–**E**) Subcutaneous tumors generated from MC38 cells were immunostained using anti-CD31 (white), anti-CD3 (red), and anti-Ki67 (green) antibodies. (**C**) Representative images of multicolor immunofluorescence staining (*n* = 5). Scale bars: 50 μm. (**D** and **E**) Quantification of Ki67^+^ T cell percentages (**D**) and vascular density (**E**) (*n* = 5). Two-way ANOVA (**A**) or 1-way ANOVA (**B**, **D**, and **E**) was used for statistical analysis.

We further compared the antitumor effects of 2 therapeutic regimens: a combination of αOX40 and anti–programmed cell death protein 1 antibody (αPD-1), and the combination of αOX40 and verteporfin. We administered 2 combination therapy regimens to C57BL/6J mice with subcutaneous tumors. We observed that either αOX40, αPD-1, or verteporfin alone slowed tumor growth (Figure 10A), and both combination therapy regimens exhibited better tumor inhibition effects (Figure 10A). Importantly, the synergistic effects of αOX40 and verteporfin were superior to those of αOX40 and αPD-1, with average tumor inhibition values of 93.49% versus 83.64% (Figure 10B). Immunostaining results demonstrated that, unlike the combination of αOX40 and αPD-1, which only enhanced T cell proliferation (Figure 10, C and D), αOX40 combination with verteporfin simultaneously boosted T cell proliferation and decreased vascular density (Figure 10, C and E). Overall, these data suggest that verteporfin effectively supplements OX40 activation by disrupting the adverse protumor effects of OX40 signaling in ECs.

## Discussion

To date, no successful monotherapies using OX40 agonists have been reported. Owing to the lack of clinical effectiveness, many pharmaceutical giants have terminated research and development of OX40 agonists. Moreover, the therapeutic efficacy of combining OX40 agonists and PD-1 inhibitors is not superior to that of continuous use of OX40 agonists (19, 49). These findings suggest that single-agent or combined application of OX40 agonists and ICBs failed to achieve satisfactory outcomes, prompting us to address this issue through NIM dimensions. In this study, we profiled the expression of OX40 in various cell types of tumor tissues and identified an unrecognized cell type that expresses OX40 and performs unique biological functions. We demonstrated that OX40 signaling exerted protumor effects by inducing EndMT in tumor ECs, which may explain the poor efficacy of OX40 agonists in clinical trials. This study revisits the impact of immunotherapy on NIM reprogramming and the duality of T cell activation therapeutic strategies.

It is generally believed that OX40 is almost exclusively expressed in T cells and is an important costimulatory molecule for T cell expansion and survival (50). OX40 signaling plays an important role in T cell survival and differentiation through ligation with OX40L (51, 52). Activation of OX40 induces high production of cytokines and generation of memory and effector T cells through various signaling pathways, including NF-κB and PI3K/AKT signaling (27). Until now, research on OX40 signaling has almost entirely focused on immune cells; however, its functions in the NIM have rarely been explored. This study revealed the biological functions of OX40 signaling in tumor ECs and elucidated its important role in tumor development and progression. Considering that NF-κB and PIK3 signals were not activated in ECs as in T cells, we believe that OX40 signal carries out its own biological functions in ECs through molecular mechanisms distinct from those in T cells. This notion was supported by the unaffected YAP protein abundance and phosphorylation after OX40 activation in T cells, as well as marginal S1P abundance in T cells compared with that in tumor ECs. The distinct signaling pathways affected by OX40 signals in T cells and tumor ECs provide a theoretical basis for the proposal of a combined therapeutic scheme. We confirmed that OX40 activation promotes angiogenesis and contributes to tumor cell infiltration into the blood vessels by triggering EndMT, which counteracts the monitoring and elimination of tumor cells by T cells. Thus, this study highlights the impact of immune activation therapy on NIM and proposes that crosstalk between the two affects the therapeutic outcome of immune activation therapy.

Furthermore, we identified YAP as a potential S1P-interacting intracellular target. S1P, a bioactive lipid secondary messenger (53), regulates complex cellular functions in the TME, acting as a signaling molecule in cell-cell communication (54). S1P exerts its physiological functions predominantly by binding to S1PRs (S1PR1–5) (39, 55), members of the G protein–coupled receptor superfamily. In this study, YAP phosphorylation and protein abundance regulated by OX40 activation were associated with an increase in intracellular S1P levels, which was not abolished by S1PR knockdown. These findings imply that S1P may function intracellularly as a secondary messenger, rather than activating signals through S1PRs present on the endothelial surface. S1P can propagate signals by interacting with intercellular targets. It regulates gene expression by binding to the histone deacetylases HDAC1 and HDAC2 (56). In mitochondria, the interaction of S1P with prohibitin 2 (PHB2) is important for cytochrome *c* oxidase assembly and mitochondrial respiration (57). In this study, based on biochemical characterization, we demonstrated that S1P could directly bind to YAP, hampering its phosphorylation. S1P acts as an upstream repressor of the Hippo pathway, inhibiting YAP phosphorylation and activating YAP/TAZ via the S1P-S1PR2 interaction in hepatocellular carcinoma cells (58, 59). In the present study, we observed that accumulated S1P disrupted the interaction of YAP and LATS1 kinase without affecting the phosphorylation of several crucial kinases of the Hippo pathway, suggesting that regulation of YAP phosphorylation and transcriptional activity by accumulated S1P in ECs is a unique mechanism.

In addition, our findings suggest that EGF may serve as a serological biomarker for predicting the efficacy of OX40 agonists. Standardizing clinical biomarkers to assess the response to immunotherapy remains challenging owing to variations in genetic signatures, TME complexities, and epigenetic onco-mechanisms (60, 61). Recently, interest has been focused on establishing neutrophil-to-lymphocyte and platelet-to-lymphocyte ratios as prognostic biomarkers to predict ICB immunotherapy efficacy (62, 63). Therapeutic effects of OX40 agonists have been observed; however, the overall response percentages are still unsatisfactory, and individual effects vary markedly (64). Thus, there is an urgent need to identify an effective predictive indicator for treatment and survival benefits that could markedly promote the clinical application of OX40 agonists. In this study, Lgr5^+^ cancer stem cells induced high OX40 expression and subsequent protumor signaling in ECs through paracrine EGF. By comprehensively evaluating the serological EGF levels in patients with CRC, we discovered that PDX tumors with low EGF levels exhibited a tumor inhibition value of approximately 40%, whereas those with high EGF levels exhibited a tumor inhibition value of less than 20% with OX40 agonists. Therefore, we preliminarily concluded that serum EGF levels could be used to indicate the therapeutic effects of OX40 agonists. Further large-scale and multicenter clinical trials are needed to identify EGF as a practical serological biomarker to assess the therapeutic efficacy of OX40 agonists in treating tumors.

Finally, we demonstrate that the combination of OX40 agonists and anti-angiogenic drugs is more effective. Agonistic antibodies against OX40 combined with ICBs have been tested against various types of cancer in early-phase clinical trials (17). Available reports on OX40 agonistic antibodies, including GSK3174998, MOXR0916, BMS-986178, MEDI0562, and PF-04518600, did not demonstrate the anticipated antitumor efficacy after combination with PD-1 blockade (19). Currently, the combination of anti-angiogenic drugs and immunotherapy has emerged as a promising strategy. The impressive response rates observed in preliminary studies using PD-L1 and VEGF antibodies support the development of such combinations (65). However, resistance to anti-angiogenic drugs may be mediated by increased hypoxia, which can also aggravate immunosuppression (66). This study provides a mechanistic explanation for the poor efficacy of OX40 agonists. We demonstrated that combining an OX40 agonistic antibody and the YAP inhibitor verteporfin induces tumor regression. The synergistic effects of the OX40 agonist and verteporfin were superior to those of the OX40 agonist and PD-1 antibody combination. This indicates that combining OX40 agonists with anti-angiogenesis therapy may be more advantageous, as they synergistically activate the antitumor activity of T cells and obstruct the protumor signals of ECs, as evidenced by increased T cell proliferation and antitumor cytokine production, and concurrently reduce vascular density in tumors. Given that we have only tested one drug combination thus far, the effects of various combinations of OX40 agonists and different anti-angiogenic drugs still need to be tested in patients with cancer.

## Methods

### Sex as a biological variable.

Our study examined male and female animals, and similar findings are reported for both sexes.

### Participants.

Human primary CRC specimens for the PDX model and tissue microarray data were obtained from the Affiliated Hospital of Jining Medical University (Supplemental Table 5). None of the patients had undergone preoperative radiation or chemotherapy. Before inclusion in the study, the participants provided written informed consent.

### Mouse experiments.

Detailed mouse experiments and additional experimental procedures are described in Supplemental Methods.

### Statistics.

Statistical analyses were conducted using SPSS (version 19.0, IBM), GraphPad Prism 5, and ImageJ (NIH) software. Two-tailed and unpaired Student’s *t* tests were used to compare the 2 groups. The data obtained from at least 3 independent experiments performed in triplicate are presented as the mean ± SEM or SD. Spearman’s correlation test was used to analyze the correlation between 2 genes. Two-way analysis of variance (ANOVA) was used for comparisons between 3 or more groups with comparable variations. If the results showed a significant difference, Student-Newman-Keuls analysis was used to determine the difference between the 2 groups. Survival curves were estimated using the Kaplan-Meier method and compared using the log-rank test. All statistical tests and *P* values were 2-sided. Statistical significance was set at *P* less than 0.05.

### Study approval.

This study was approved by the Research Ethics Committee of the Affiliated Hospital of Jining Medical University. All animal experimental protocols were approved by the Animal Ethics Committee of the Affiliated Hospital of Jining Medical University (no. 2022B021).

### Data availability.

Original/source data of RNA sequencing and scRNA-Seq were deposited in the Genome Sequence Archive and can be accessed via accession numbers HRA006890 and HRA004557, respectively.

## Author contributions

Bin Zhang, QB, and HX designed the study, directed the project, and supervised data analysis. BH, RZ, BZ, and HP performed and analyzed most experiments. Jilan Liu and YW performed and assessed the immunofluorescence staining. Baogui Zhang, LH, and DY provided primary CRC specimens and corresponding serum samples and followed up the survival status and prognosis. GD, Xu Zhang, and Xiao Zhang provided technical support in primary cell isolation and culture. Jing Liang, MW, and MZ constructed the PDX models. SW, FD, JZ, YZ, Xu Zhang, and Xiao Zhang assisted with construction of PDX models, xenograft tumor models, tumor metastasis models, and drug treatment. BH, QB, HX, Bin Zhang, and RZ wrote the manuscript. All authors edited the manuscript.

## Supplementary Material

Supplemental data

Unedited blot and gel images

Supplemental table 1

Supplemental table 2

Supplemental table 3

Supplemental table 4

Supplemental table 5

Supplemental table 6

Supporting data values

## Version 1 03/03/2025

Electronic publication
