# Supplementary material for: Endothelial OX40 activation facilitates tumor cell escape from T cell surveillance through S1P/YAP-mediated angiogenesis
Source: J Clin Invest. 2025 Mar 3;135(5):e186291. doi: 10.1172/JCI186291 (PMC11870743; doi:10.1172/JCI186291)
Supplement: Supplemental data [file jci-135-186291-s061.pdf]

Supplementary Data

Supplementary Figures and Figure legends

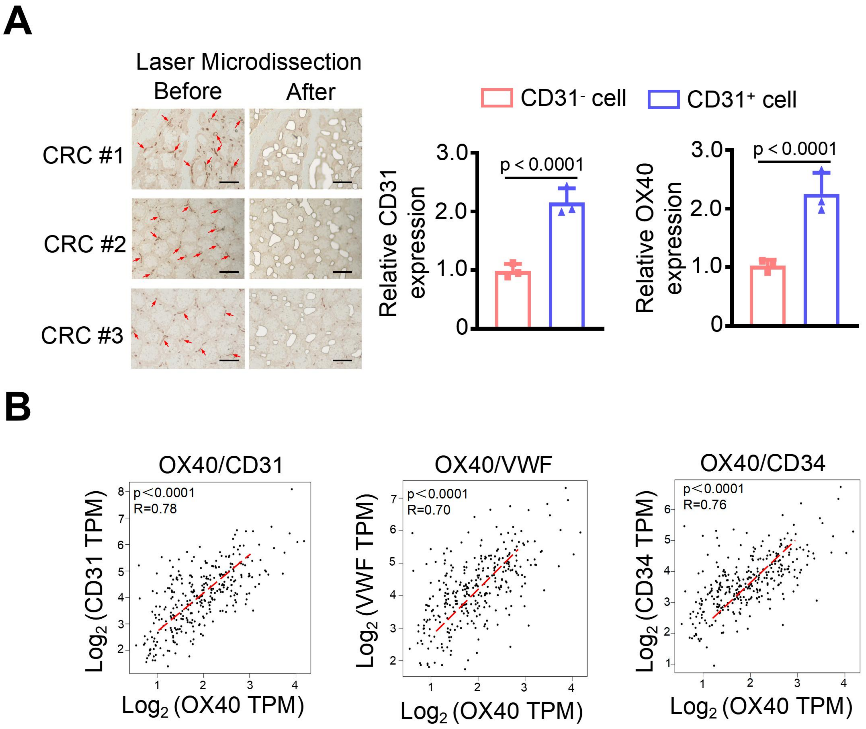

Supplemental Figure 1. Correlation analysis between OX40 and endothelial cell marker genes. (**A**) $CD31^+$ endothelial cells in CRC tiuuses were obtained using the laser capture micro-dissection method. CD31 and OX40 expression was measured using the qRT-PCR method (n = 3). The red arrows indicate cell regions that were excised for further analysis. Scale bar = 100 µm. (**B**) Correlations of OX40 with CD31, VWF, or CD34 were assessed using the Cancer Genome Atlas Colon Adenocarcinoma dataset accessed from GEPIA online programme. Statistical analyses, p-values in (**A**) were calculated using a two-tailed Student's *t*-test. P-values in (**B**) were obtained using the Spearman correlation analysis.

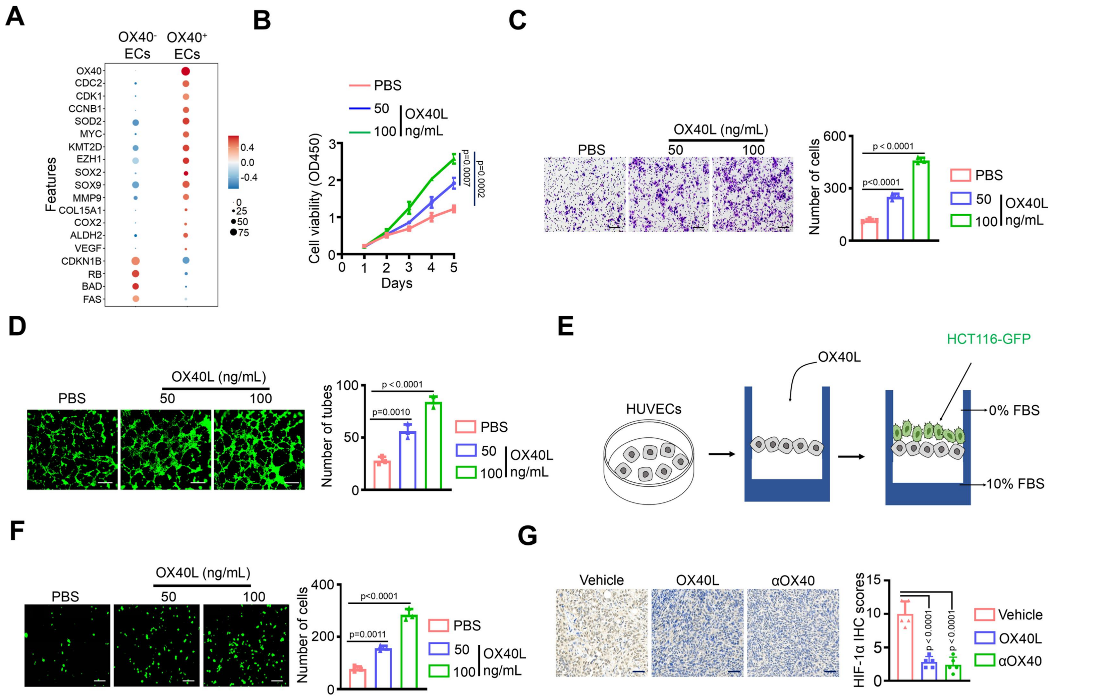

Supplemental Figure 2. Elucidating the biological function of OX40 signal in endothelial cells. (**A**) Genes associated with cell proliferation, cell stemness, metastasis, and drug resistance; key transcription factors involved in tumorigenesis; and tumor suppressor genes were evaluated in OX40$^+$ or OX40$^-$ endothelial cells (ECs) from colorectal cancer (CRC) tissues using single-cell RNA-sequencing data. (**B**-**F**) Human umbilical vein ECs (HUVECs) were pre-treated with media from SW480 cells for 48 h. Assessment of cell viability (**B**), migratory ability (**C**), tube forming capacity (**D**), and tumor cell transendothelial migratory ability (**E** and **F**) in HUVECs treated with PBS or soluble human OX40L protein (50 or 100 ng/mL) at the indicated concentration (n = 3). Scale bar = 30 μm. (**G**) IHC staining for HIF1α protein in subcutaneous tumor tissues treated with OX40L or αOX40. Scale bar = 50 μm. Statistical analyses, p-values in (**B**) were calculated the two-way ANOVA. P-values in (**C**), (**D**), (**F**) and (**G**) were calculated using the one-way ANOVA.

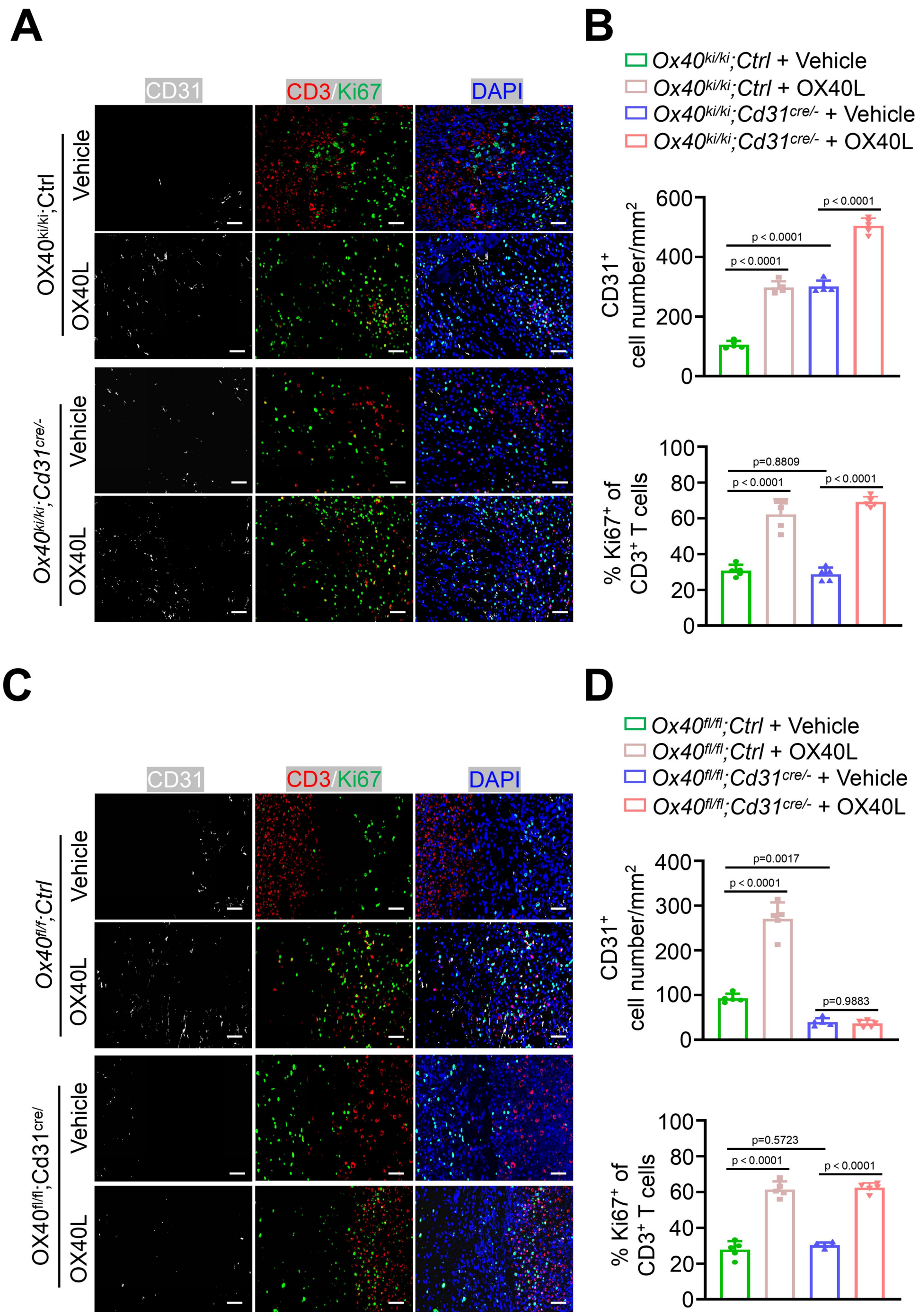

Supplemental Figure 3. Multicolor immunofluorescence staining for subcutaneous tumors from ECs conditional OX40 knock-in or knock-out mice. (**A-D**) Representative images of multicolour immunofluorescence using anti-CD31 (white), anti-CD3 (red), and Ki67 (green) antibodies for subcutaneous tumors from ECs conditional OX40 knock-in (**A**, n = 5) or knock-out (**C**, n = 5) mice. Statistical

analysis of vascular density (**B**) and Ki67^{+} T-cell percents (**D**). Scale bar=50 µm (n = 5). Statistical analyses, p-values in (**B**) and (**D**) were calculated using the one-way ANOVA.

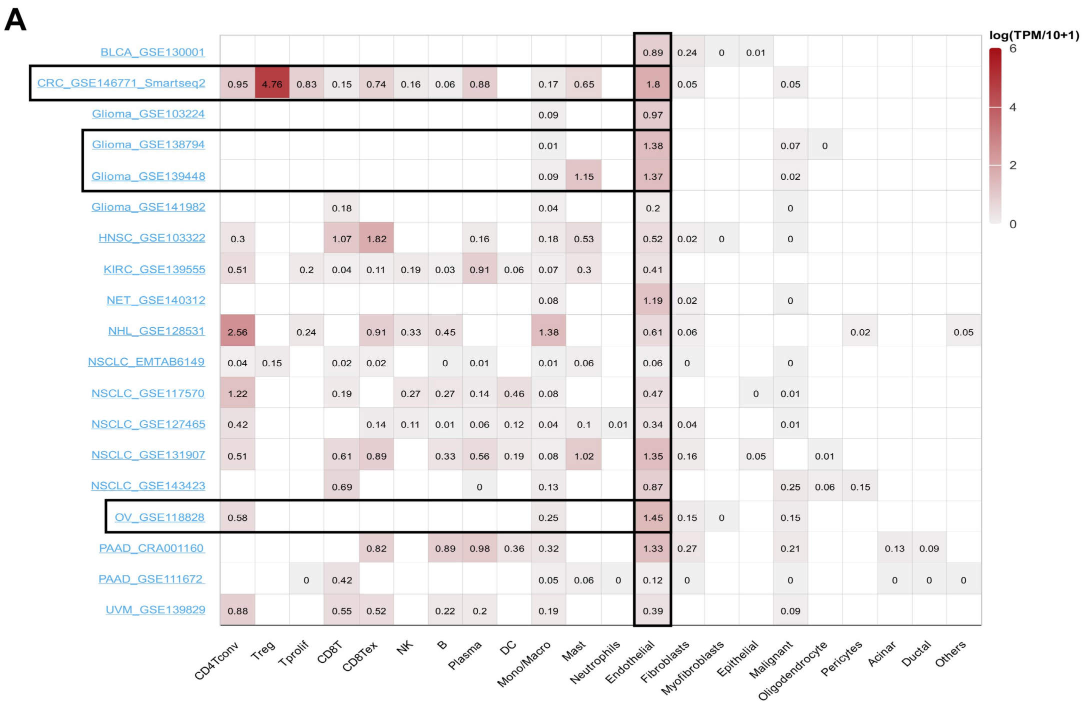

Supplemental Figure 4-1. Selecting cancer types harboring high OX40 expression in endothelial cells. (**A**) OX40/TNFRSF4 gene expression was evaluated using the open-access data from the TISCH single-cell transcriptome database (http://tisch.comp-genomics.org/).

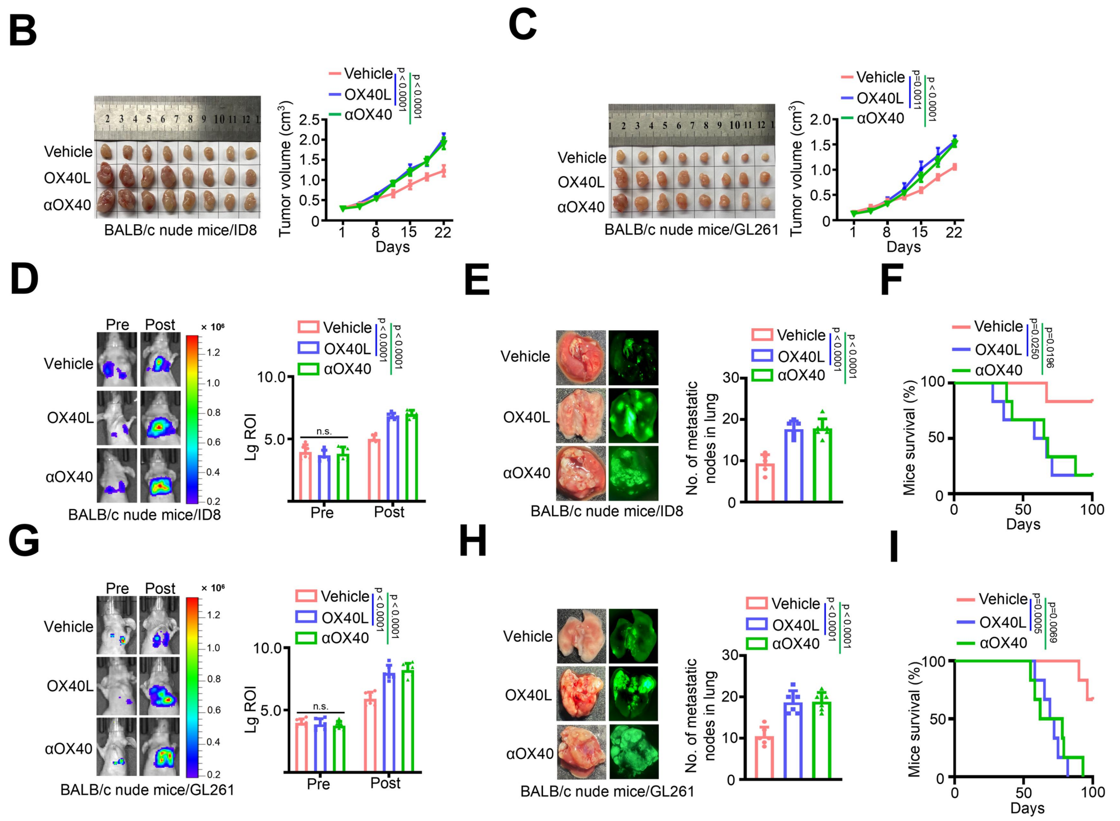

Supplemental Figure 4-2. Confirming the pro-tumor effects of OX40 signalling in ECs among pan-cancer. (**B** and **C**) Images (left) and volume (right) of subcutaneous tumors established using ID8 cell (**B**) or GL261 cell (**C**) treated with mouse OX40L protein (OX40L, 200 mg/mouse, I.P.) or anti-mouse OX40 agonistic antibody (αOX40, 100 μg/mouse, I.P.) in Balb/c nude mice (n = 8). (**D–I)** Bioluminescent intensity (**D** and **G**), metastatic nodules in the lung (**E** and **H**), and survival of mice from the pulmonary metastasis model (**F** and **I**) established using ID8 cell or GL261 cell treated with mouse OX40L protein (OX40L, 200 mg/mouse, I.P.) or anti-mouse OX40 agonistic antibody (αOX40, 100 μg/mouse, I.P.) in Balb/c nude mice (n = 6). Statistical analyses, p-values in (**B**) and (**C**) were calculated the two-way ANOVA. P-values in (**D**), (**E**), (**G**) and (**H**) were calculated using the one-way ANOVA. P values in (**F**) and (**I**) were calculated using the log-rank test.

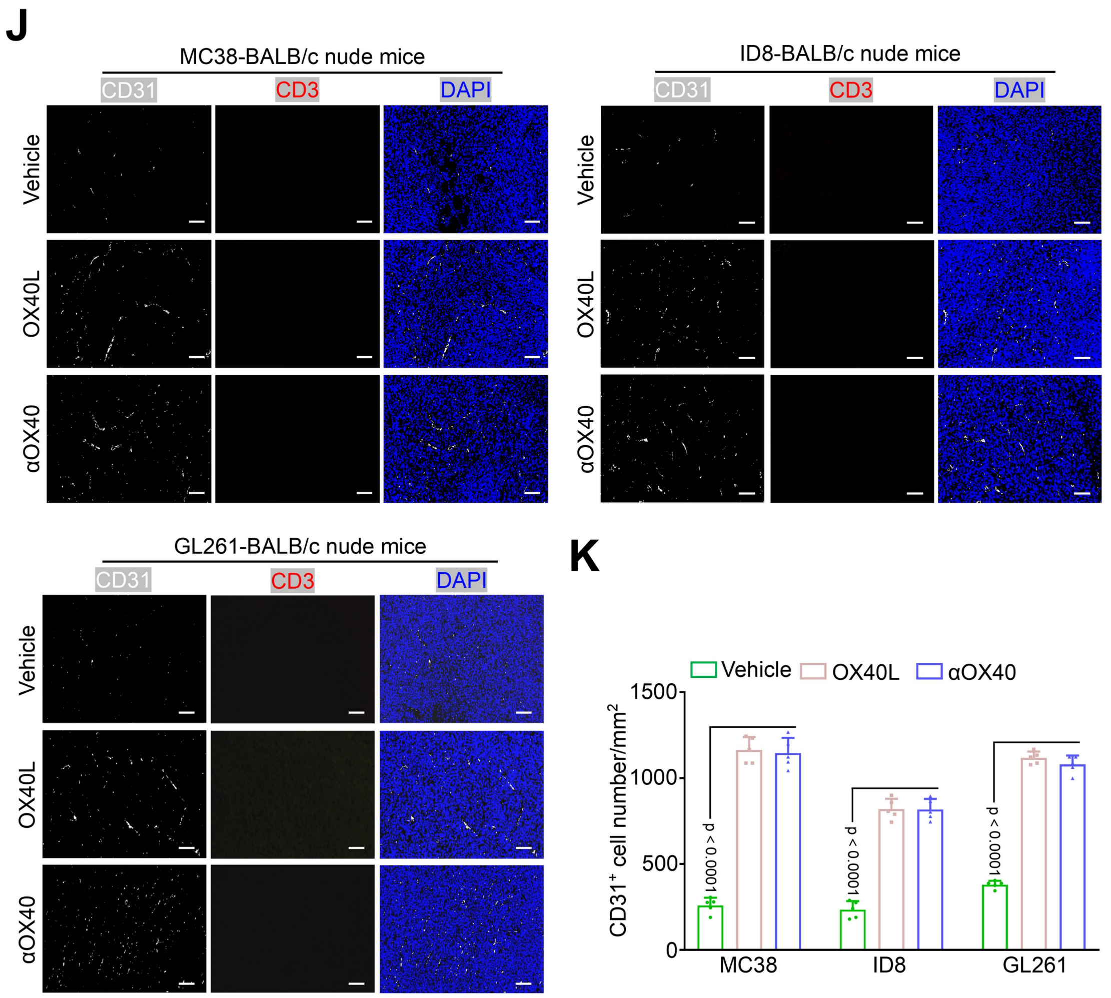

Supplemental Figure 4-3. Confirming the pro-angiogenesis effects of OX40 signalling in ECs among pan-cancer. (**J**) Representative images of multicolour immunofluorescence using anti-mouse CD31 (white) and anti-mouse CD3 (red) antibodies in indicated subcutaneous tumors (n = 5). Scale bar = 50 µm. (**K**) Statistical analysis of vascular density (n = 5). Statistical analyses, p-values in (**K**) were calculated using the one-way ANOVA.

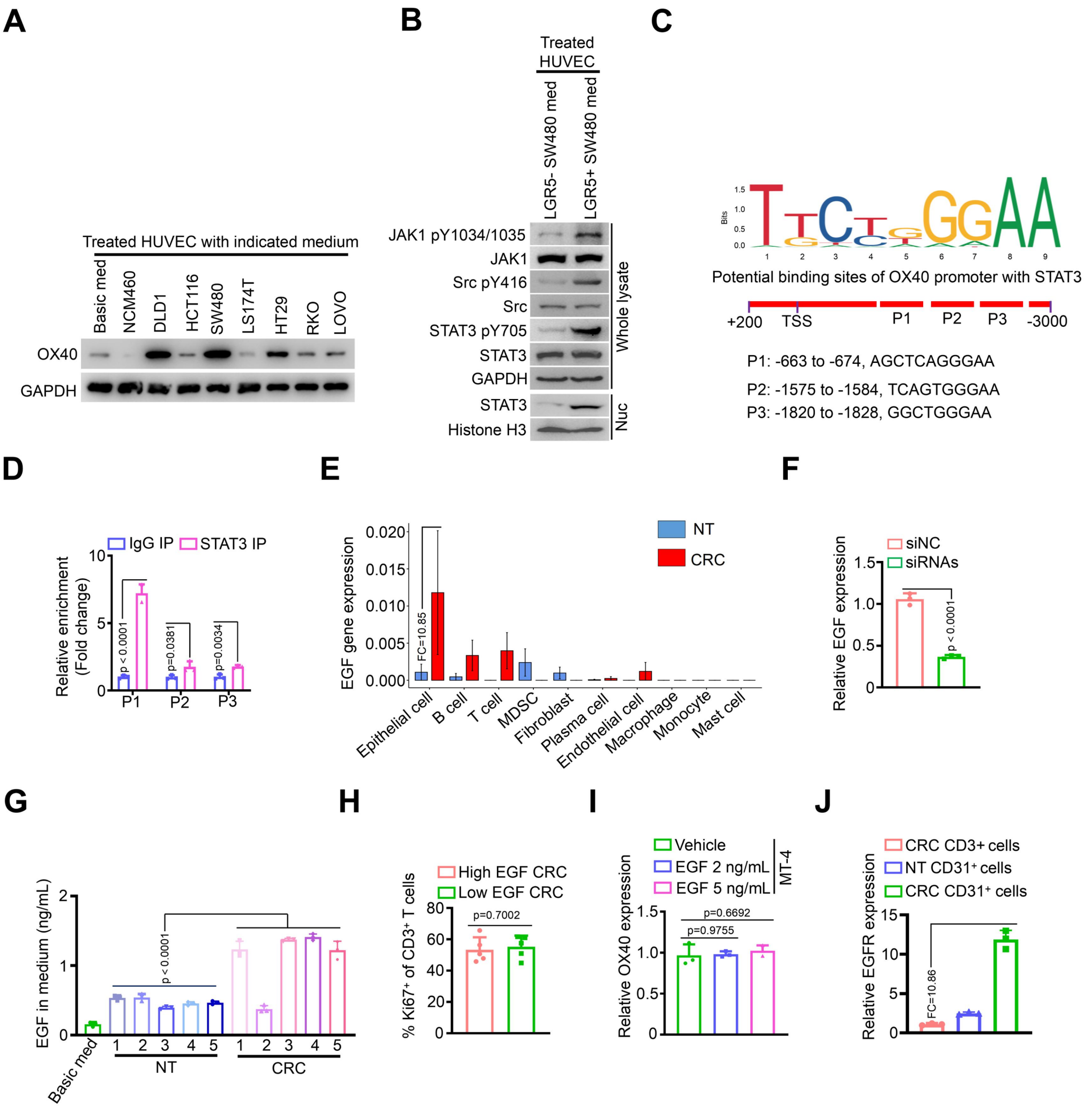

Supplemental Figure 5. Clarifying the specific mechanism of how EGF activates OX40 expression through STAT3 signalling. (**A**) The protein levels of OX40 were measured in HUVECs treated with media derived from indicated cells using the western blotting analyses. (**B**) HUVECs were treated with media derived from indicated cells. Whole-cell lysate and nuclear fraction were obtained and immunoblotted with the indicated antibodies. (**C**) Potential regions of the OX40 gene promoter where STAT3 transcription factor binds with predicted using the JASPAR online programme (https://jaspar.elixir.no/). (**D**) Chromatin

immunoprecipitation-qPCR analysis was applied to confirm the enrichment of different OX40 promoter regions using STAT3 antibody and a negative antibody (IgG) in HUVECs (n = 3). (**E**) Gene expression of EGF in various cell subsets of CRC and NT tissues using single-cell RNA sequencing data (n = 5). (**F**) Silence efficiency of siRNAs targeting EGF gene was evaluated using the qRT-PCR method (n = 3). (**G**) EGF levels were measured in media from shredded CRC tissues and control NT tissues using the enzyme-linked immunosorbent assay. (n = 3). (**H**) Statistical analysis of Ki67^{+} T-cell percents in tumor tissues from CRC patients with high or low serum EGF levels (n = 5). (**I**) OX40 gene expression was evaluated in MT-4 cell treated with vehicle or different amounts of EGF using the qRT-PCR method (n = 3). (**J**) T cells and ECs were sorted from CRC tissues or NT tissues. EGFR gene expression in the those cells was detected using the qRT-PCR method (n =3 technical replicates). FC, Fold change. Statistical analyses, p-values in (**D**), (**F**), and (**H**) were calculated using a two-tailed Student's *t*-test. P-values in (**G**) and (**I**) were calculated using the one-way ANOVA.

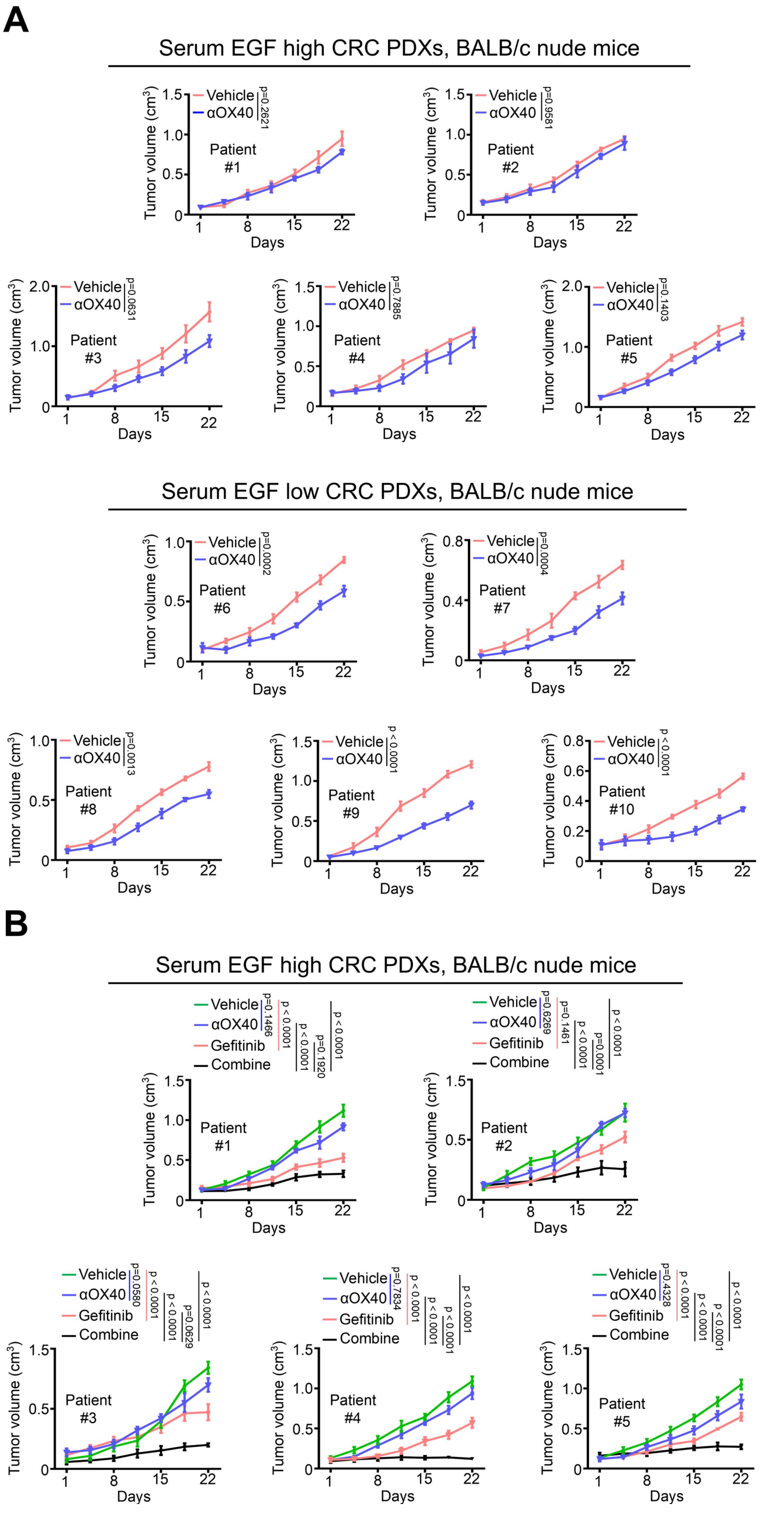

Supplemental Figure 6. Therapeutic effects of the anti-OX40 antibody and EGFR inhibitor on patient-derived xenografts (PDXs) with CRC patients with high or low serum EGF levels. (**A**) Based on Fig. 3K, we used 10 tumor tissues from CRC patients with low (5 cases) and high (5 cases) serum EGF levels to construct

PDX models. Mice bearing those PDXs were treated using anti-human αOX40 (20 μg/mouse, I.P.). The volume of subcutaneous tumors was calculate (n = 7 or 8). (**B**) Five tumor tissues from CRC patients with high serum EGF levels were uesd to established PDX models. Then, mice bearing those PDXs were treated with anti-human αOX40 (20 μg/mouse, I.P.), EGFR inhibitor Gefitinib (100 mg/kg, oral gavage) or the drug combination. The volume of subcutaneous tumors was calculate (n = 7 or 8). Statistical analyses, p-values in (**A**) and (**B**) were calculated using the two-way ANOVA.

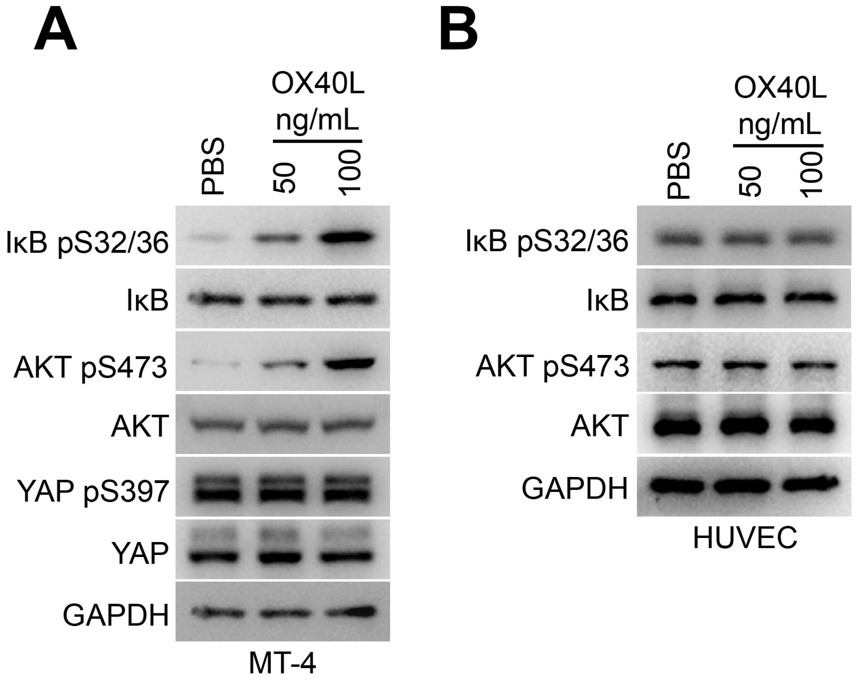

Supplemental Figure 7. Impacts of the OX40 signal on the PI3K-AKT or NF-κB signalling pathway in T cells and endothelial cells. (**A** and **B**) MT-4 cells (**A**) and HUVECs (**B**) were treated with PBS or human OX40L protein at the indicated concentration. Cell lysates were immunoblotted using the indicated antibodies.

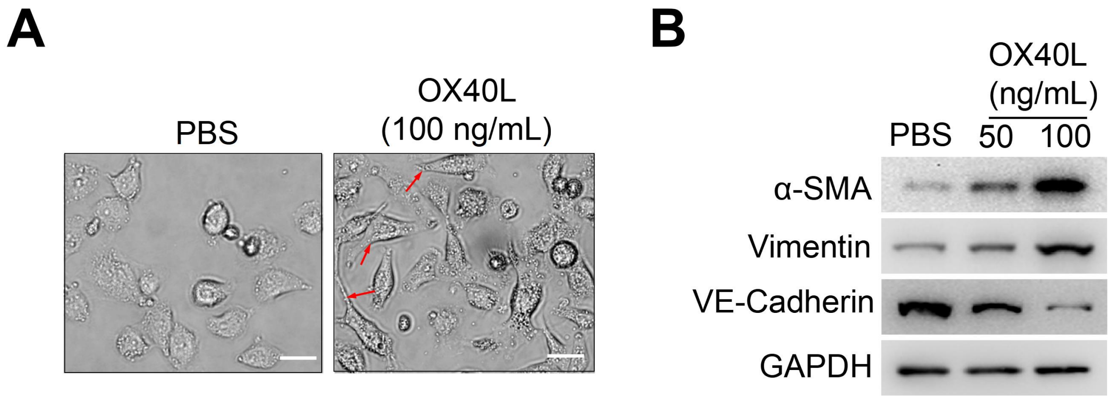

Supplemental Figure 8. Impacts of the OX40 signal on endothelial cell morphology and endothelial mesenchymal transition (EndMT)-associated genes expression. (**A**) Morphological images of HUVECs treated with PBS or OX40L as assessed via phase contrast microscopy. Red arrows indicate cell pseudopodia. Scale bar = 5 µm. (**B**) HUVECs were treated with PBS or OX40L at the indicated concentrations. The cell lysate was immunoblotted using the indicated antibodies.

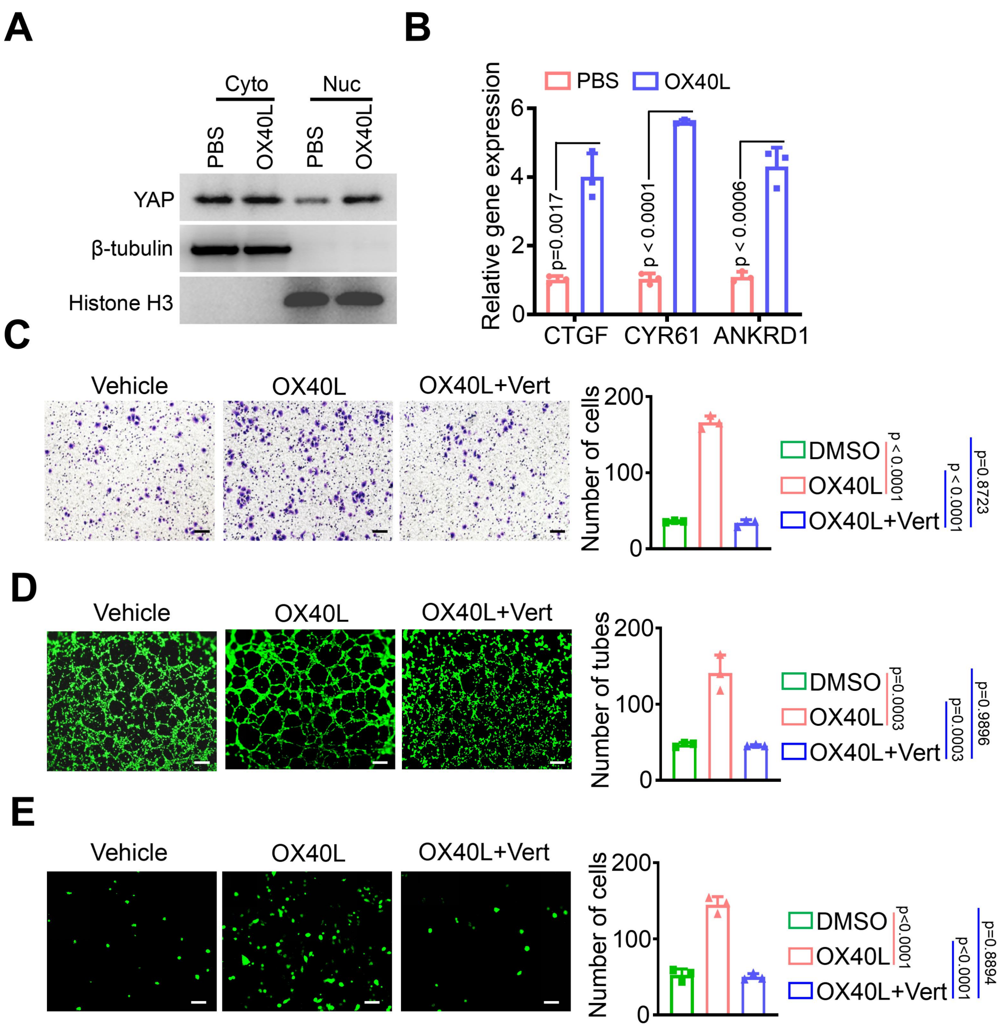

Supplemental Figure 9. Confirming that the OX40 signal regulates endothelial cell function by modulating YAP nuclear translocation. (**A**) HUVECs were treated with PBS or human OX40L protein (100 ng/mL). Cell lysates were fractionated into cytoplasmic (Cyto) and nuclear (Nuc) fractions and blotted with the indicated antibodies. (**B**) YAP downstream genes expression was evaluated in HUVECs treated with PBS or human OX40L protein (100 ng/mL) using the qRT-PCR method (n = 3). (**C**–**E**) Assessment of migratory ability (**C**), tube-forming capacity (**D**), and tumor cell transendothelial migratory ability (**E**) in HUVECs treated with PBS, human OX40L protein (100 ng/mL), or OX40L plus Verteporfin (10 μM) (n = 3). Scale bar = 30 μm. Statistical analyses, p-values in (**B**) were calculated using a two-tailed Student's *t*-test.

P-values in (**C**-**E**) were calculated using the one-way ANOVA.

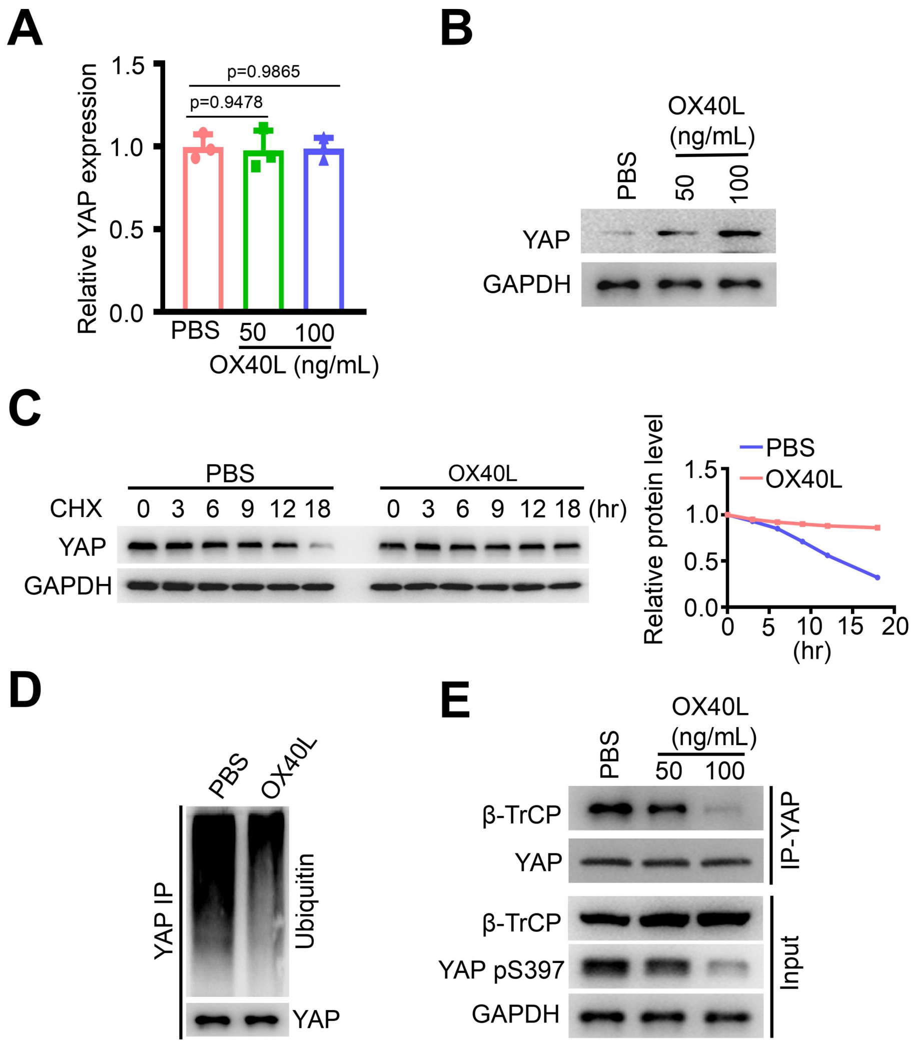

Supplemental Figure 10. Exploring the specific pattern of the OX40 signal regulating YAP expression.

(**A** and **B**) YAP expression was measured in HUVECs treated with PBS or human OX40L protein at the indicated concentration using the qRT-PCR method (**A**, n = 3) and western blotting analyses (**B**). (**C**) HUVECs were treated with PBS or human OX40L protein (100 ng/mL). Cells were treated with 50 μM cycloheximide (CHX) for the indicated times. Cell lysates were blotted with anti-YAP antibody. Western blots are shown in the left panel. The quantification of YAP protein levels using

ImageJ is shown in the right panel. (**D** and **E**) HUVECs were treated with PBS or human OX40L protein at the indicated concentration. Cell lysates were immunoprecipitated using anti-YAP antibody, followed by blotting with anti-ubiquitin (**D**) or anti-β-TrCP (**E**) antibodies. Statistical analyses, p-values in (**A**) were calculated using the one-way ANOVA.

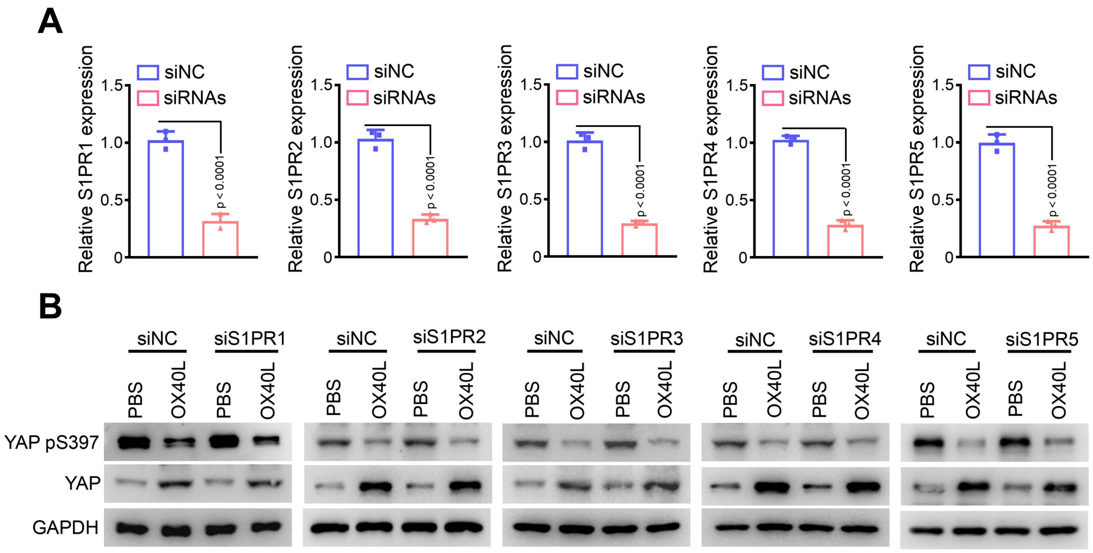

Supplemental Figure 11. Clarifying whether the OX40 signal functions in endothelial cells through S1P receptors (S1PRs). (**A**) Silence efficiency of siRNAs targeting S1PR1, S1PR2, S1PR3, S1PR4, and S1PR5 genes was evaluated using the qRT-PCR method (n = 3). (**B**) HUVECs were treated with PBS, human OX40L protein (100 ng/mL), or OX40L plus siRNAs against S1PR1, S1PR2, S1PR3, S1PR4, or S1PR5, respectively. Cells were subjected to immunoblotting using the indicated antibodies. Statistical analyses, p-values in (**A**) were calculated using a two-tailed Student's *t*-test.

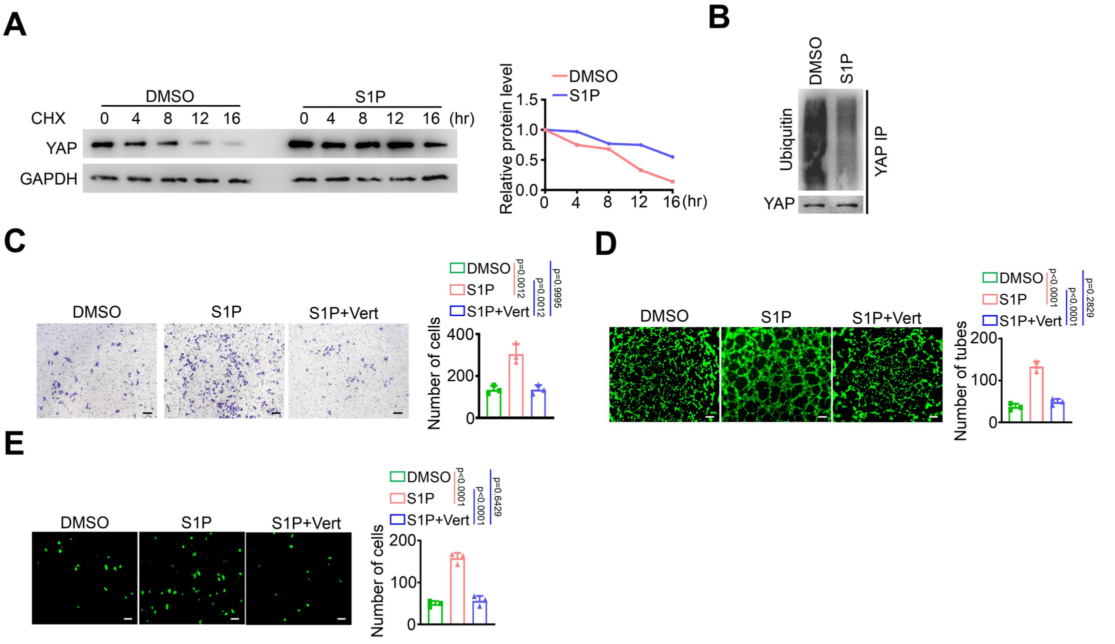

Supplemental Figure 12. Confirming that S1P regulates endothelial cell function by modulating YAP nuclear translocation. (**A**) HUVECs were treated with DMSO or S1P. Cells were treated with 50 µM cycloheximide (CHX) for the indicated times. Cell lysates were blotted with a YAP antibody. Western blots are shown in the left panel. The quantification of YAP protein levels using ImageJ is shown in the right panel. (**B**) HUVECs were treated with DMSO or S1P. Cell lysates were immunoprecipitated using an anti-YAP antibody, followed by blotting with an anti-ubiquitin antibody. **(C–E)** Assessment of migratory ability (**C**), tube-forming capacity (**D**), and tumor cell transendothelial migratory ability (**E**) in HUVECs treated with PBS, S1P, or S1P plus Verteporfin (n = 3). Scale bar = 30 µm. Statistical analyses, p-values in (**C-E**) were calculated using the one-way ANOVA.

A

YAP-p-LATS1 interaction molecular docking

Index	Without S1P	With S1P
Confidence score	1.00	1.00
Docking score	-85.16	-30.51

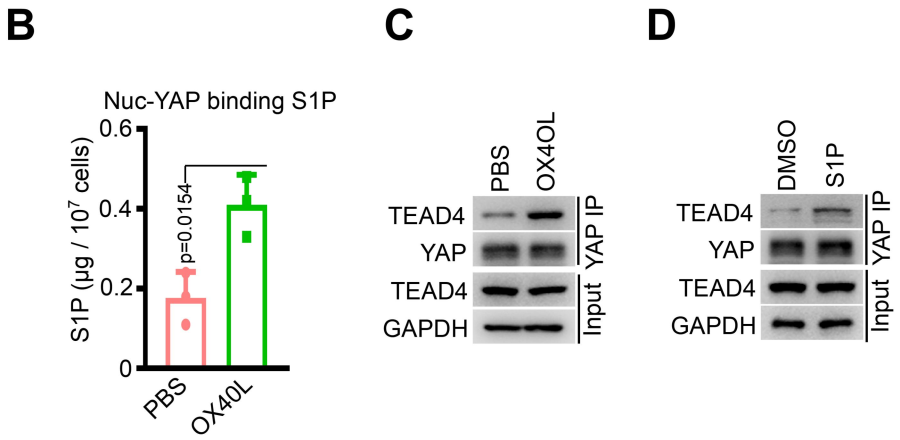

Supplemental Figure 13. Impacts of the OX40 signal or S1P on YAP-TEAD4 interaction. (**A**) Docking scores of the interactions between YAP and p-LATS1 with or without the participation of S1P. (**B**) HUVECs were treated with PBS or OX40L. Lysates of nuclear fraction were obtained and immunoprecipitated using an anti-YAP antibody. Metabolites in the immunocomplex were extracted using methanol. The quantitative abundance of S1P was measured using an LC-MS-based trace-level metabolite detection method (n = 3). (**C** and **D**) HUVECs were treated with OX40L (**C**) or S1P (**D**) and corresponding vehicles. Cell lysates were immunoprecipitated with an anti-YAP antibody and blotted with the indicated antibodies. Statistical analyses, p-values in (**B**) were calculated using a two-tailed Student's *t*-test.

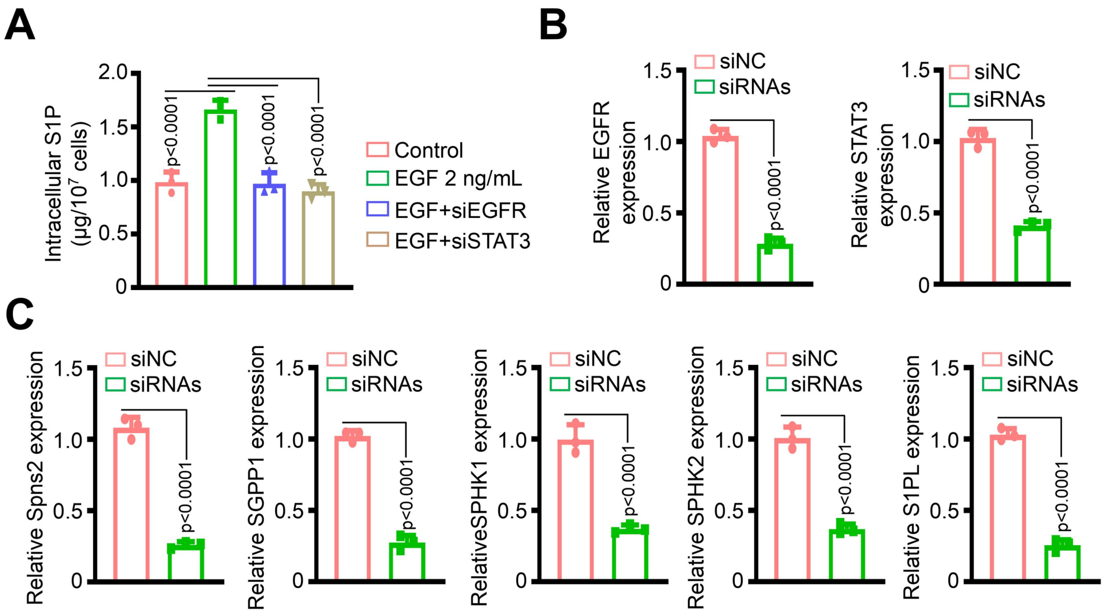

Supplemental Figure 14. The impacts of EGF-STAT3 signalling on intracellular S1P abundance in HUVECs. (**A**) Quantitative abundance of S1P was measured in HUVECs treated with EGF, EGF plus EGFR siRNA, or EGF plus STAT3 siRNA, as analysed using S1P-targeted metabolomics measurement (n = 3). (**B** and **C**) Silence efficiency of siRNAs targeting EGFR, STAT3, Spns2, SGPP1, SPHK1, SPHK2, and S1PL genes was evaluated using the qRT-PCR method (n = 3). Statistical analyses, p-values in (**A**) were calculated using the one-way ANOVA. p-values in (**B** and **C**) were calculated using a two-tailed Student's *t*-test.

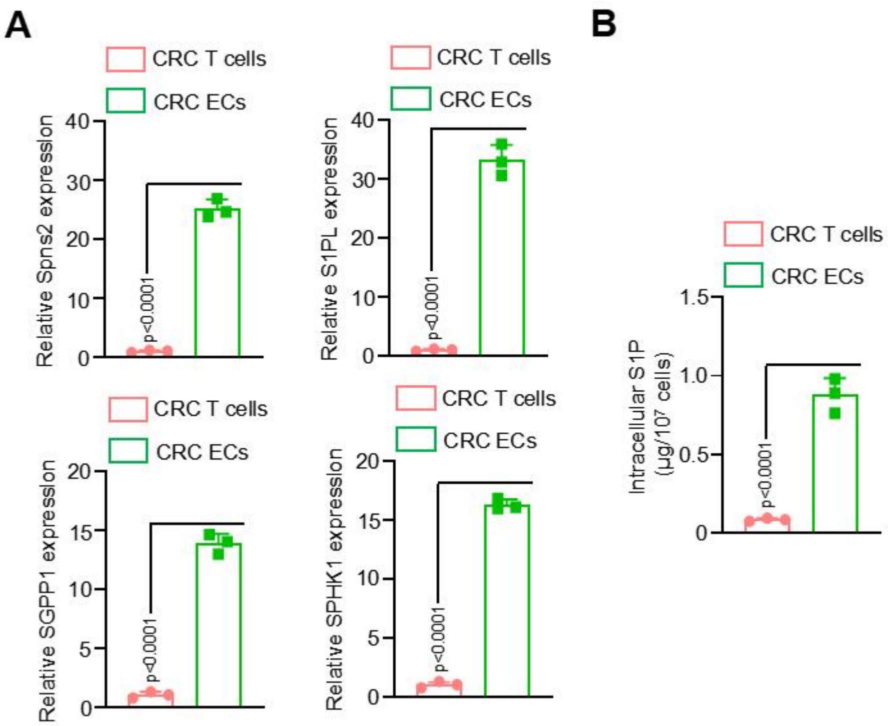

Supplemental Figure 15. Comparing the expression of S1P-metabolism associated genes and S1P S1P abundance in T cells and ECs sorted from CRC tissues. (**A**) T cells and ECs were sorted from tumor tissues using anti-CD3 and anti-CD31 beads, respectively. Spns2, S1PL, SGGP1, and SPHK1 expression was evaluated in T cells and ECs using qRT-PCR (n = 3). (**B**) The quantitative abundances of intracellular S1P were measured via the S1P-targeted metabolomics analysis (n = 3). Statistical analyses, p-values in (**A** and **B**) were calculated using a two-tailed Student's *t*-test.

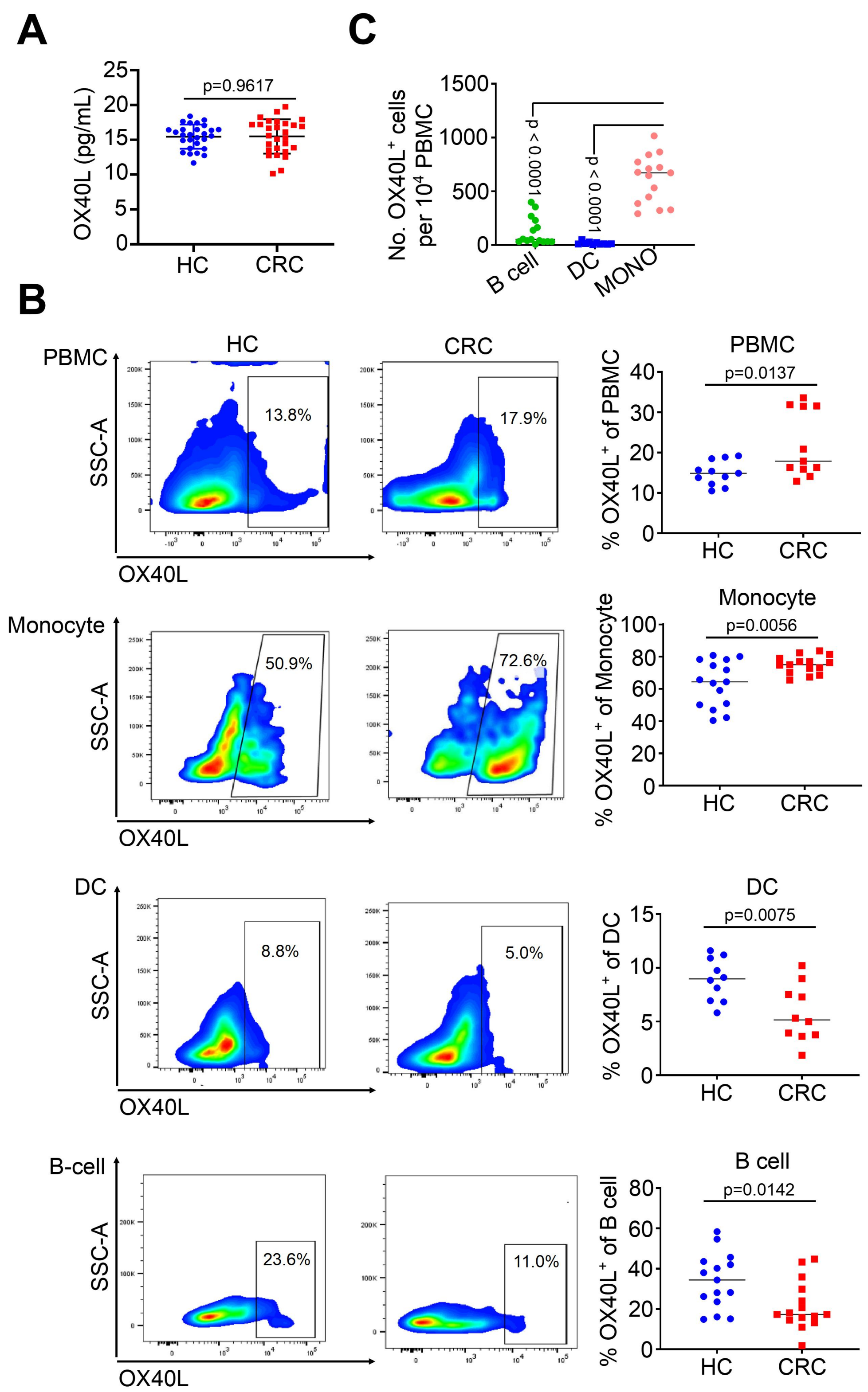

Supplemental Figure 16. Exploring the upstream signal source for OX40 activation in endothelial cells. (**A**) OX40L levels in the sera of patients with colorectal cancer (CRC) and healthy controls (HCs) were measured using the

enzyme-linked immunosorbent assay (n = 28). (**B**) Sorting mononuclear cells from the peripheral blood of patients with CRC and HCs. OX40L positive percents in peripheral blood mononuclear cell (PBMC, n = 11), B cells (n = 15), dendritic cells (DCs, n = 10), and monocytes (n = 15) in patients with CRC and HCs were evaluated and compared. DCs in PBMC were identified by $CD11c^+$ cells. B cells in PBMC were identified by $CD19^+$ cells. Monocytes in PBMC were identified by $CD14^+$ cells. (**C**) First, we counted the numbers of B cells, DCs, and monocytes per 10,000 PBMCs in the peripheral blood of patients with CRC. Based on the positive percentages of OX40L in these cells, the absolute numbers of $OX40L^+$ B cell, $OX40L^+$ DC, and $OX40L^+$ monocyte per 10,000 PBMCs were obtained. Statistical analyses, p-values in (**A**) and (**B**) were calculated using a two-tailed Student's *t*-test. P-values in (**C**) were calculated using the one-way ANOVA.

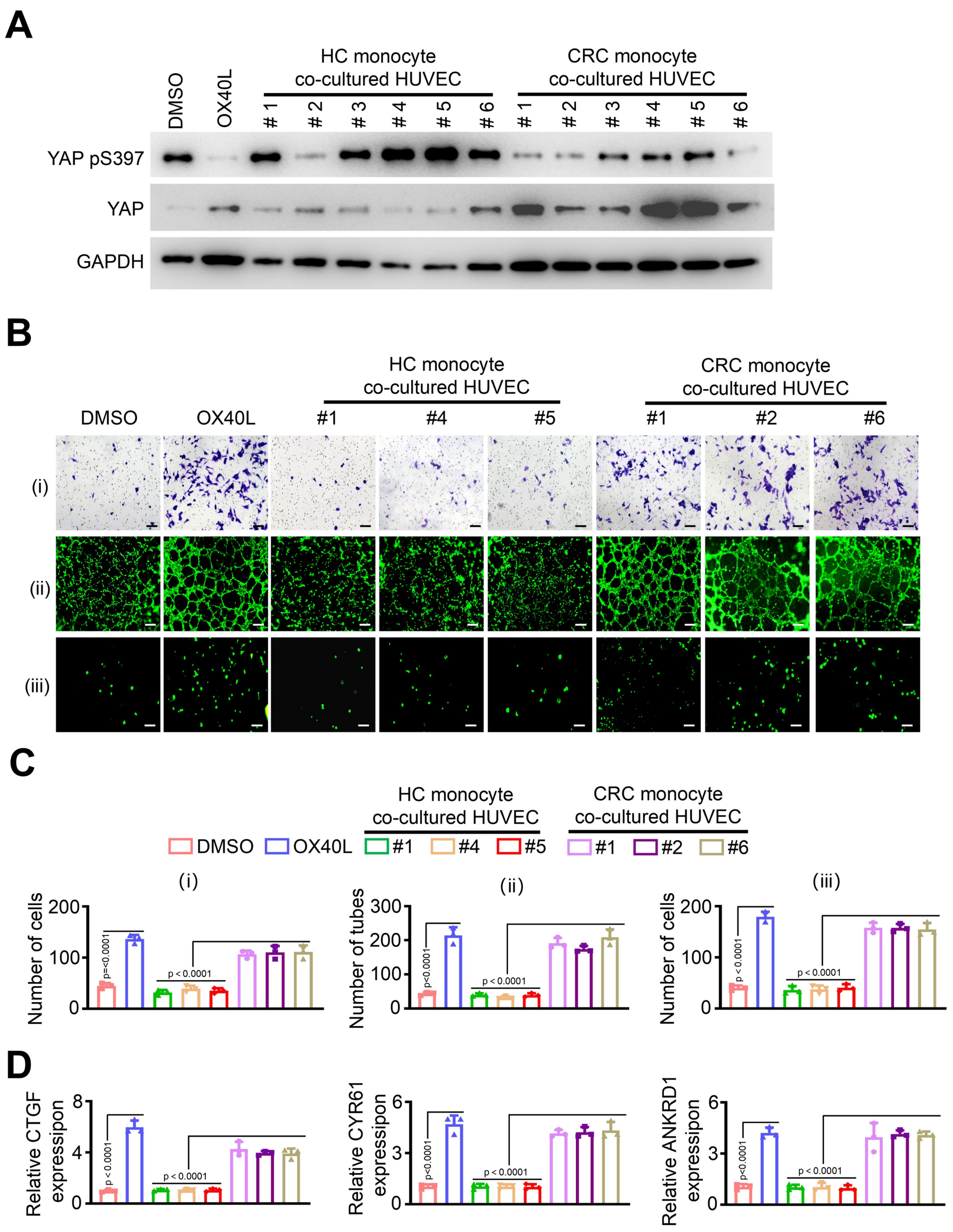

Supplemental Figure 17. Clarify the effects of monocytes in activating OX40 signalling and regulating biological functions in endothelial cells. (**A–D**) Human monocytes were isolated from the peripheral blood of patients with colorectal cancer (CRC) and healthy controls (HCs). HUVECs were co-cultured with sorted indicated monocytes for 48 h. Then, several experiments were conducted using HUVECs. Cell

lysates of HUVECs were immunoblotted using indicated antibodies (**A**). (i) Migratory ability, (ii) tube-forming capacity, and (iii) tumor cell transendothelial migratory ability were assayed (**B** and **C**, n = 3). (**D**) YAP downstream genes expression was evaluated in HUVECs using the qRT-PCR method (n = 3). Scale bar = 30 μm. Statistical analyses, p-values in (**C**) and (**D**) were calculated using the one-way ANOVA.

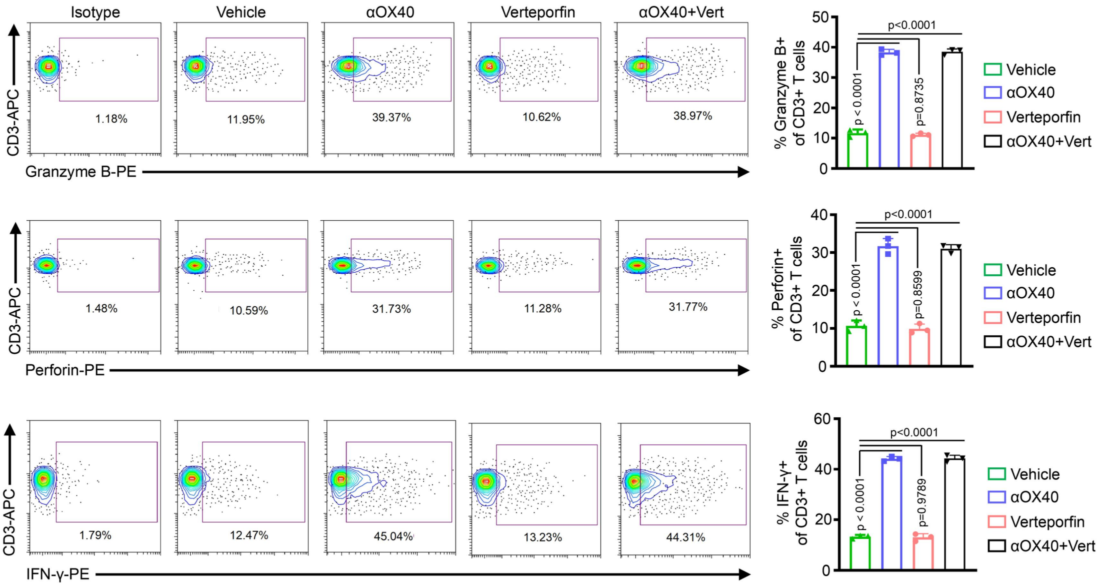

Supplemental Figure 18. Evaluating the production capacity of anti-tumor cytokines in T cells. Percentages of tumor-infiltrating Granzyme B-producing, Perforin-producing, and IFN-γ-producing $CD3^+$ T cells in PDX tumors treated with vehicle, anti-human αOX40 (20 μg/mouse, I.P.), Verteporfin (60 mg/kg, I.P.) or αOX40 and Verteporfin combination. T cells were identified by $CD3^+$ cells.

Statistical analyses, p-values were calculated using the one-way ANOVA.

Supplemental Methods

Mice experiments

Subcutaneous tumor model

For the subcutaneous tumor model, three million indicated cells were injected subcutaneously and bilaterally into BALB/c nude (VSM20001), NSG (VSM20002), or C57BL/6J (VSM10001) mice, which were obtained from Beijing Viewsolid Biotech (Beijing, China). Once tumor sizes reached 0.1-0.15 cm^3, the mice were randomly assigned into different groups and treated with vehicle, recombinant mouse OX40L protein (200 mg/mouse, twice a week, I.P., SinoBiological, #53582-M04H), anti-mouse OX40 antibody (100 μg/mouse, I.P., BioXCell, #BE0031), anti-mouse PD-1 antibody (200 μg/mouse, I.P., BioXCell, #BE0146), and Verteporfin (60 mg/kg, twice a week, I.P., Selleck, #S1786). The drug treatment lasted for 3 weeks. Tumor volume was measured at the indicated time points and calculated as length × $width^2/2$.

Colon orthotopic tumor model

In the orthotopic mouse colon cancer model, mouse colon cancer MC38 cells were stably transfected with the firefly D-luciferin-GFP expression vector. NSG mice were anesthetized with chloral hydrate. A midline incision was made into the abdominal cavity of each mouse. Using a sterilized microsyringe, a 30-μL cell suspension containing 500,000 indicated cells (pre-mixed with matrix) was gently injected into the distal colon. The incisions were closed using 6-0 nylon sutures. After two weeks, tumor imaging was performed using the FluorVivo imaging system (INDEC

Biosystems, Santa Clara, CA, USA). Thereafter, the mice were the randomly assigned to different groups and treated with vehicle, recombinant mouse OX40L protein (200 mg/mouse, twice a week, I.P., SinoBiological, #53582-M04H), or anti-mouse OX40 antibody (100 μg/mouse, I.P., BioXCell, #BE0031). Drug treatment lasted for 3 weeks. Survival analysis was performed based on mouse survival status and time. Luciferase-positive regions were imaged using an IVIS Lumina II (Caliper Life Sciences). Finally, the mice were sacrificed and liver metastases were evaluated based on GFP signals using an OV100 microscope (Olympus).

Tail-vein injection metastasis model

In the tail-vein injection lung metastasis mouse model, mouse colon cancer MC38 cells, glioma GL261 cells, and ovarian cancer ID8 cells were stably transfected with firefly D-luciferin-GFP expression vector. Two million cells were injected into the tail vein of athymic BALB/c nude or NSG mice. Thereafter, the mice were randomly assigned to different groups and treated with vehicle, recombinant mouse OX40L protein (200 mg/mouse, twice a week, I.P., SinoBiological, #53582-M04H), or anti-mouse OX40 antibody (100 μg/mouse, once weekly, I.P., BioXCell, #BE0031). Drug treatment lasted for 3 weeks. Survival analysis was performed based on the mouse survival status and time. Luciferase-positive regions were imaged using IVIS Lumina II (Caliper Life Sciences). Finally, the mice were sacrificed and lung metastases were evaluated based on GFP signals using an OV100 microscope (Olympus).

Splenic injection for liver metastasis models

A splenic injection of LUC-GFP-MC38 cells was performed to establish a preclinical murine model of hepatic metastasis. Briefly, trypsinized LUC-GFP-MC38 cells were resuspended in cold phosphate-buffered saline (PBS) at a final concentration of 10^6/mL. The mice were anesthetized with isoflurane, and laparotomy was performed on the left abdomen. Thereafter, 100-μL of MC38 cells were injected slowly into the spleen of immunocompetent transgenic *Ox40*$^{fl/fl}$*;Cd31*$^{cre/-}$ mice and *Ox40*$^{fl/fl}$*;Ctrl* or *Ox40*$^{ki/ki}$*;Cd31*$^{cre/-}$ and *Ox40*$^{ki/ki}$*;Ctrl* mice. Thereafter, the mice were randomly assigned to different groups and treated with vehicle or recombinant mouse OX40L protein (200 mg/mouse, twice a week, I.P., SinoBiological, #53582-M04H). Drug treatment lasted for 3 weeks. Luciferase-positive regions were imaged using IVIS Lumina II (Caliper Life Sciences). Mice were euthanized at the study endpoint for analysis and sampling. Finally, the mice were sacrificed and liver metastases were evaluated based on luciferase signals.

Antibody-mediated cell depletion

Anti-mouse CD3 antibody (BioXCell, #BE0001-1) or an immunoglobulin G (IgG) control was injected into the mice (200 μg/mouse, I.P.) twice weekly for 2 weeks. After establishing subcutaneous tumors, anti-CD3 antibody or IgG control was administered intraperitoneally once a week until the end of the experiment.

PDX model

Mouse PDX experiments were conducted following protocols and ethical guidelines approved by the Institutional Animal Care and Use Committee. Primary human CRC tumor fragments (2-3 mm in diameter) were subcutaneously injected into 6-week-old

female immunodeficient BALB/c nude mice. Patients had not undergone any treatment or procedures. Anti-human OX40 antibody (20 μg/mouse, I.P., GPLBIO, #GC69303), Verteporfin (60 mg/kg, twice a week, I.P., Selleck, #S1786), and Gefitinib (80 mg/kg, twice a week, oral gavage, Selleck, #S1025) were administered. Drug treatment lasted for 3 weeks. The tumor volume was measured at the indicated time points and calculated as length × width^2/2.

Generation of transgenic OX40$^{fl/fl}$;Cd31$^{cre/-}$ mice

Single-stranded RNAs (sgRNAs), suitable for the CRISPR/Cas9 system, were designed to target exons 3-7 of *Tnfrsf4*. These sgRNAs can accurately guide Cas9 to its target site. To achieve finger site cleavage, we synthesized a DNA template containing the desired mutation. These templates were obtained through the chemical synthesis of DNA fragments or PCR amplification, ensuring the correct loci and containing repair template sequences compatible with the CRISPR/Cas9 system. The designed sgRNA sequence and repair template were inserted into the CRISPR/Cas9 vector to construct a donor for editing. We injected the screened CRISPR/Cas9 vector into fertilized eggs and implanted the fertilized eggs into the uterus of a surrogate mother mouse. After pregnancy, some mice carried the required sequence. Genotyping of newborn mice was conducted by PCR amplification and sequencing to confirm the presence of the target mutation. *Tnfrsf4* flox mice were hybridised with endothelial Cre (Tek-Cre) mice to obtain flox-homozygous and Cre-positive mice, achieving conditional *Tnfrsf4* knockout in ECs. The transgenic mice were established by GemPharmatech (Nanjing, China).

Generation of transgenic OX40$^{ki/ki}$;Cd31$^{cre/-}$ mice

The gRNA to *Tnfrsf4-ROSA26* gene, the donor vector containing the 'CAG promoter-loxP-PGK-Neo-6*SV40 pA-loxP-Kozak-mouse *Tnfrsf4* CDS-rBG pA' cassette, and Cas9 mRNA were co-injected into fertilized mouse eggs to generate targeted conditional knock-in offspring. F0 founder animals were identified using PCR and sequencing and were bred with wild-type mice to test germline transmission and F1 animal generation. We bred F1-targeted mice with mice carrying a tissue-specific Tek-Cre deletion to generate mice that were heterozygous for the targeted allele and hemizygous/heterozygous for the Cre transgene. Mouse genotyping primers and probes are listed in Table S6.

Cell culture and transfection

The mouse CRC cell line MC38 (iCell Bioscience, #iCell-m032), mouse glioma GL261 cells (iCell Bioscience, iCell-m063), mouse ovarian cancer ID8 cells (iCell Bioscience, #iCell-m064), and the human CRC cell lines HCT116 (iCell Bioscience, #iCell-h071), SW480 (ATCC, #CCL-228), LS174T (ATCC, #CL-188), HT29 (ATCC, #HTB-38), and LOVO (ATCC, #CCL-229) were cultured in Dulbecco's Modified Eagle's Medium (DMEM) supplemented with 10% foetal bovine serum (FBS). The human CRC cell lines, DLD1 (ATCC, #CCL-221) and RKO (ATCC, #CRL-2577), and the human colon epithelial cell line, NCM460 (iCell Bioscience, #iCell-h373), were cultured in RPMI 1640 medium supplemented with 10% FBS. HUVECs were cultured in DMEM supplemented with 10% FBS and 1% NEAA. The cells were

incubated at 37 °C in a humidified atmosphere containing 5% CO_2. Regular testing for mycoplasma contamination was performed. The cell lines were authenticated by Genetica DNA Laboratories using STR profiling. Transfection was performed using Lipofectamine 3000 reagent according to the manufacturer's instructions.

Gene silencing

The siRNAs targeting the indicated genes and scrambled siRNA controls were purchased from Biotend (Shanghai, China). siRNAs were transfected into cells using Lipofectamine 3000. Cells were trypsinized at 48-72 h post-transfection for various assays. The sequences of shRNAs and siRNAs are listed in Table S6.

Laser capture microdissection (LCM) of CD31$^+$ ECs

The human CRC tissues were frozen in isopentane. Serial 10-µm sections of the CRC tissues were cut longitudinally on a Jung Frigocut 2800E cryostat at -20 °C and mounted onto Superfrost Plus glass slides at room temperature. Sections were immediately fixed in 70% ethanol for 30 s, washed with distilled water, rinsed in 95% ethanol, immersed in filtered eosin-Y for 10 s, dehydrated in 100% ethanol, and washed consecutively for 5, 10, and 15 min with fresh xylene. Slides were air-dried for 1 h and transferred to a desiccator at room temperature. An Arcturus PixCell II LCM system equipped with an Olympus microscope (Arcturus Engineering, Mountain View, CA, USA) was used to capture principal cells from the sections. One LCM cap (Capture Transfer Film TF100, Arcturus) was used per tissue section, and

optimal conditions for LCM included a laser power of 40 mW, duration of 1.5-2.5 ms, and laser spot size of 7.5 m for epithelia. The captured cells were mixed with TRIzol lysis buffer in an Eppendorf tube, microcentrifuged, and stored at 80 °C. RNA was extracted within 72 hours. The cell capture process, from tissue sectioning to lysis, is completed rapidly to limit RNA degradation.

RNA extraction and quantitative real time-PCR (qRT-PCR)

Total RNA was extracted and purified using an RNeasy Mini Kit (Qiagen,# 74104) following the manufacturer's instructions. Thereafter, 1 μg of total RNA was reverse-transcribed using the PrimeScript RT kit (TaKaRa, #RR047R). QRT-PCR was performed using a QuantiTect SYBR Green PCR kit (Qiagen,# 204141). GAPDH was used as the reference gene for normalization. The mean of three independent analyses was calculated for each gene. Fold-changes were determined by relative quantification ($2^{\Delta\Delta}$Ct). The qRT-PCR primer sequences are listed in Table S6.

Coimmunoprecipitation (Co-IP) and western blotting

Cells were lysed in WB&IP buffer supplemented with protease inhibitors. Cell lysates were immunoprecipitated with the indicated primary antibodies overnight at 4°C, followed by protein A/G precipitation for 2 h or direct incubation with magnetic beads conjugated with tagged antibodies. The beads were washed thrice with lysis buffer and eluted in SDS sample buffer. The eluted immune complexes were separated by sodium dodecyl sulfate-polyacrylamide gel electrophoresis (SDS-PAGE), followed by

western blotting. Equal amounts of total protein were separated on 7.5/10/12.5% SDS-polyacrylamide gels and transferred onto nitrocellulose membranes. Membranes were incubated overnight with primary antibodies against the target proteins. The membranes were washed thrice with 1× TBST buffer and incubated with secondary antibodies for 1 h at room temperature. The signals were visualized using Luminata Crescendo Western horseradish peroxidase substrate. Antibodies in this study include anti-phospho-YAP Ser127 (CST, #13008, 1:1000 dilution), anti-phospho-YAP Ser397 (CST, #13619, 1:1000 dilution), anti-YAP (CST, #14074, 1:2000 dilution), anti-phospho-IκBα Ser32/36 (CST, #9246, 1:5000 dilution), anti-IκBα (Proteintech, #10268-1-AP, 1:2000 dilution), anti-phospho-Akt Ser473 (Proteintech, #66444-1-Ig, 1:500 dilution), anti-Akt (Proteintech, #60203-2-Ig, 1:2000 dilution), anti-α-SMA (CST, #19245, 1:1000 dilution), anti-Vimentin (CST, #5741, 1:2000 dilution), anti-VE-Cadherin (CST, #2158, 1:1000 dilution), anti-β-tubulin (ABclonal, #AC015, 1:4000 dilution), anti-phospho-MOB1 Thr35 (CST, #8699, 1:2000 dilution), anti-MOB1 (CST, #13730, 1:1000 dilution), anti-phospho-MST1 Thr183 (CST, #49332, 1:2000 dilution), anti-MST1 (Proteintech, #22245-1-AP, 1:500 dilution), anti-phospho-LATS1 Thr1079 (CST, #8654, 1:1000 dilution), anti-LATS1 (CST, #3477, 1:1000 dilution), anti-β-TrCP (ABclonal, A21951, 1:1000 dilution), anti-OX40 (CST, #61637, 1:2000 dilution), anti-GAPDH (ABclonal, #AC001, 1:4000 dilution), anti-Histone H3 (CST, #4499, 1:4000 dilution), anti-Spns2 (Abcam, #ab59972, 1:500 dilution), and anti-TEAD4 (ABclonal, #A4151, 1:500 dilution).

Immunofluorescence staining

A total of 1×10^5 cells were seeded in a 24-well culture plate (slides were placed in advance) and the culture medium was discarded after 48 h of culture. The cells were fixed in 4% paraformaldehyde and permeabilized with 0.5% Triton X-100. Cells were blocked with 5% bovine serum albumin. Primary antibodies were added and incubated overnight at 4°C on a shaker. The fluorescent secondary antibody was diluted with the blocking solution and incubated in the dark for 1 h at room temperature. The nuclei were stained with DAPI for 5 min. The slides were mounted using a fluorescent mounting medium. An upright fluorescence microscope was used to observe and assess the staining index of positive cells.

For multiple IHC of tissue slices, experiments were performed following the instructions of the Panovue TSA kit (Panovue, #10079100020). Briefly, the sections were treated with 0.5% Triton X-100 for 30 min, blocked with goat serum for 1 h at room temperature, and washed with PBS three times between each step. The sections were incubated with antibodies including anti-mouse CD3 (Abcam, #ab231775, 1:200 dilution), anti-CD3 (Abcam, #ab237707, 1:200 dilution), anti-CD31 (Abcam, #ab182981, 1:200 dilution), anti-Ki67 (Abcam, #ab15580, 1:500 dilution), anti-YAP (CST, #14074, 1:100 dilution), and anti-OX40 (CST, #61637, 1:200 dilution) overnight at 4 °C in a humidified chamber, followed by incubation with secondary antibodies and DAPI. Anti-fade mounting medium was used to seal the sections. Immunofluorescence images were obtained using a confocal microscope.

Immunohistochemistry (IHC)

IHC was performed using an EnVision HRP kit (Dako, #K400811). Briefly, paraffin-embedded tumor tissues were deparaffinized in xylene and re-hydrated in gradient ethanol. Then, antigen was retrieved by boiling the sample for 60 min. Samples were incubated with an anti-HIF1α antibody (CST, #48085, 1:100 dilution) at 4 °C overnight. The sections were stained with secondary antibody for 30 min at room temperature and then stained with DAB reagent. Staining was independently assessed by two individuals. Wherever the IHC scores were plotted, the staining score was based on the staining strength and the number of positive cells. The positive staining rate was scored as follows: 0 points for less than 5%, 1 point for 5-25%, 2 points for 26-50%, 3 points for 51-75%, and 4 points for over 75%. The staining strength was scored as follows: non-staining, 0 points; light yellow, 1 point; brown-yellow, 2 points; and brown, 3 points. The final score was the product of the two scores.

HUVEC migration assay

HUVECs suspended in serum-free medium were placed in the upper chamber of a 24-well Transwell system with polycarbonate filters (8-μm pores, Corning). Thereafter, 500 μL of the conditioned medium was added to the lower chamber. After 12 h of incubation, cells that had migrated to the bottom of the membranes were stained with 0.25% crystal violet for 20 min, followed by imaging and counting.

HUVEC tube formation assay

We pre-coated 48-well plates with 150 μL precooled Matrigel (Corning, #354234) per well and polymerized at 37 °C for 30 min. HUVECs (1×10^4 cells) suspended in 200 μL of the conditioned medium were seeded into each well and cultured for 3 h. Thereafter, fields of the tube structure were randomly chosen and photographed for quantification.

Tumor transendothelial migration assay

HUVEC monolayers on transwell inserts were cultured in DMEM medium containing 10% FBS for 4 h at 37 ℃. The inserts were placed in 24-well plates and coated with a thin layer of matrigel. GFP-labeled HCT116 cells were added to the top chamber with HUVECs and allowed to migrate through HUVECs. After 48 h of migration, the cells on the top chamber were removed, and the cells in the bottom chamber were fixed and counted. The results were quantified by counting the number of HCT116 cells passing through the endothelium in the same field (20×) and were expressed as standardized values for at least three independent experiments. The assay was quantified in at least three independent experiments, with each transwell counting five fields.

Flow cytometry

To perform surface staining, we mixed the appropriate antibodies with the cells at room temperature for 15 min and washed them with DPBS. For intracellular cytokine

detection, we used the Fixation Permeabilization Kit (BD, #554714) to fix and permeabilize the cells, followed by staining with the appropriate antibodies in the Permeabilization buffer for 30 min at 4 °C. The following antibodies were used: anti-mouse CD45-PerCP (BioLegend, #103129), anti-mouse CD3-PE (BioLegend, #100205), anti-human CD3-APC (BioLegend, #317317), anti-human/mouse Granzyme B-PE (BioLegend, #372207), anti-human Perforin-PE (BioLegend, #308105), anti-human INF-γ-PE (BioLegend, #383303). The cells were further analyzed using flow cytometry (BD LSRFortessa) and FlowJo software (BD Biosciences).

Nuclear/cytoplasmic fractionation

The cell pellets were resuspended in 1 ml fractionation buffer (0.1% NP-40, complete Protease Inhibitor, PhosSTOP, and PMSF in PBS), gently pipetted 15 times, and immediately centrifuged at 13,500 × g for 30 s. The supernatants were labelled as cytoplasmic fractions. The pellets were washed twice with fractionation buffer and dissolved in 160 μL of fractionation buffer as the nuclear fraction. Each fraction was sonicated for 10 s at 60% output.

Ubiquitination assay

The ubiquitination assay was performed according to established protocols. Briefly, the indicated cells were treated with the proteasome inhibitor MG132 (50 mM, Selleck, #S2619) for 6 h before harvesting. Cell extracts were subjected to

immunoprecipitation and western blotting with antibodies against ubiquitin.

Untargeted metabolomics analysis

Metabolite extraction

For cultured cells, approximately 10^7 cells were harvested, and 800 μL of cold methanol/acetonitrile (1:1, v/v) was added to remove proteins and extract metabolites. The mixture was transferred to a new centrifuge tube and centrifuged at 14,000×g for 5 min at 4°C. The supernatant was collected, and the remaining solution was dried using a vacuum centrifuge at 4°C. For liquid chromatography-MS (LC-MS) analysis, the dried samples were re-dissolved in 100 μL of acetonitrile/water (1:1, v/v) solvent and transferred to LC vials.

LC-MS analysis

To assess polar metabolites in untargeted metabolomics, extracts were analyzed using a Sciex TripleTOF 6600 quadrupole time-of-flight mass spectrometer. The mass spectrometer was coupled with hydrophilic interaction chromatography via electrospray ionisztion (ESI). LC separation was performed on an ACQUIY UPLC BEH Amide column (2.1 mm × 100 mm, 1.7 μm particle size, Waters, Ireland) using a gradient of solvent A (25 mM ammonium acetate and 25 mM ammonium hydroxide in water) and solvent B (acetonitrile). The mass spectrometer was operated in the negative and positive ionization modes. The product ion scan was obtained using information-dependent acquisition in high-sensitivity mode. The following parameters were used: fixed collision energy, 35 ± 15 eV; declustering potential, 60 V (+) and -60

V (-); exclusion of isotopes within 4 Da; and monitoring of 10 candidate ions per cycle.

Quantitative measurement of S1P

The homogenate was sonicated on ice for 30 min, and the mixture was centrifuged for 10 min at 14,000 × g, 4°C. Subsequently, 500 μL of the supernatant was used to extract metabolites using a hydrophilic-lipophilic balance elution system. The system was pre-activated with 200 μL of methanol and equilibrated with 200 μL of water. The loaded system was washed successively with 200 μL of water and 200 μL of 10% methanol aqueous solution and eluted with 50 μL of acetonitrile. Analyses were performed using a UHPLC system (I-Class LC, Waters) coupled to a QTRAP mass spectrometer (AB Sciex 5500). The mobile phase comprised solvents A (0.1% formic acid in water) and B (0.1% formic acid in acetonitrile). The samples were kept in the automatic sampler at 4°C, with a column temperature of 45°C. The gradient was run at a flow rate of 400 μL/min, and a 4 μL aliquot of each sample was injected. The gradient started at 30% B from 0 to 1 min, linearly increased to 80% B from 1 to 7 min, further increased to 90% B from 7 to 9 min, and was sustained at 90% B from 9 to 11 min. Quality control samples were used to test and evaluate the system's stability and repeatability. In the ESI negative mode, the following conditions were set: source temperature at 450°C, Ion Source Gas1 at 55, Ion Source Gas2 at 60, Curtain gas at 30, and Ion Spray Voltage Floating at 4,500 V. The multiple reaction monitoring (MRM) mode was used to detect ion pairs. The Multiquant software was

used to extract the chromatographic peak areas and retention times. The relative quantitative analysis of each metabolite was based on the peak area.

Single-cell transcriptome sequencing

Sample collection, library preparation and sequencing of scRNA

Five patients diagnosed with primary CRC were recruited for this study. The scRNA-sequencing was conducted on the Illumina sequencing platform by Genedenovo Biotechnology Co., Ltd. (Guangzhou, China). Single cells were isolated and sorted from freshly dissected tumors using standard protocols. In brief, tumors were cut into 1 mm^3 pieces and enzymatically digested using the MACS Tumor Dissociation Kit (Miltenyi, #130-095-929) according to the manufacturer's instructions. The dissociated cells were then passed through a 70-μm cell-strainer and centrifuged. After removing the supernatant and lysing the red blood cells, single-cell transcriptome amplification was performed using the SMART-seq2 protocol. Libraries were prepared using the chromium controller and10× Chromium Next GEM Single Cell 3' v3.1 protocol. The cell suspension was mixed with the master mix and loaded onto a chromium Next GEM chip G along with Single Cell 3' v3.1 Gel Beads and Partitioning Oil. Within the droplets, RNA transcripts from single cells were uniquely barcoded and reverse-transcribed. The resulting cDNA molecules were pooled and subjected to end repair, addition of a single 'A' base, and ligation of adapters. Subsequently, the products were purified and enriched using PCR to generate the final cDNA library. Finally, the libraries were sequenced on the

Illumina HiSeq platform, following the read length specifications provided in the user guide.

scRNA-seq data processing

Raw sequencing data from scRNA-seq were processed using Cell Ranger 3.0.2 (10× Genomics). Filtering was applied to scRNA-seq data, and a gene expression matrix was generated using DNBelab C Series scRNA analysis software (MGI). The reference genome (ensemble assembly: Sscrofal1.1) was downloaded for analysis. Cells were retained based on criteria including the detection of more than 200 and less than 5000 genes and a mitochondrial gene detection percentage (MT%) of less than 30%. Following the generation of UMI count profiles, Seurat 3.0 was utilized for quality control and subsequent analysis. The gene expression measurements for each cell were normalized using the 'LogNormalize' method, which scales the total expression by a default scale factor (10,000) and applies a logarithmic transformation to each dataset. Data alignment involved the selection of 1,000 highly variable genes from each data matrix, followed by the implementation of the 'FindIntegrationAnchors' and 'IntegrateData' functions in Seurat 3.0. Subsequently, clustering was performed using the 'FindClusters' function in Seurat to identify subcell-type clusters. UMAP visualization was used to represent the clusters of each dataset if they were derived from both donors. To identify differentially expressed marker genes in each cluster, the 'FindAllMarkers' function in Seurat, based on the Wilcoxon rank-sum test, was utilized. The top 10 differentially expressed genes (DEGs) in each cluster were visualized using a heatmap generated by Seurat. Cell

cycle scoring was conducted using the 'CellCycleScoring' function in Seurat, with cell cycle phase marker genes.

RNA-Sequencing

RNA was isolated from HUVECs treated with PBS or human OX40L recombinant protein (Elabscience, Cat#: PKSH032843) using the TRIzol reagent. Each sample was purified using an RNeasy Mini Column (Qiagen, Limburg, Netherlands), treated with DNase, and assessed for quality using an Agilent 2100 Bioanalyzer. The samples were subjected to paired-end sequencing (2×100 bp) using the Illumina HiSeq 2000 platform. Read mapping to the human genome (hg19) was performed using TopHat v2.0.11 (http://tophat.cbcb.umd.edu) with default options and a TopHat transcript index generated from Ensembl_GRCh37. RNA-sequencing was performed by Shanghai G&C Biotechnology Co., Ltd. (Shanghai, China). To identify differentially expressed genes (DEGs) between the two samples, the expression level of each transcript was calculated as fragments per kilobase of exon per million mapped reads (FPKM). Fold change ≥ 2.5 or ≤ -2.5, and adjusted p-value < 0.01 were the criteria to obtaining DEGs.

Trace-level quantitation of S1P binding with YAP

Protein immunocomplex preparation

To test the direct binding of S1P to YAP, we expressed the recombinant YAP WT construct in *Escherichia coli* cells and purified both proteins to homogeneity. For *in*

vitro S1P-YAP binding detection, 1.0 μg of purified 6× His-YAP WT protein was incubated with S1P in 1 mL adjusted immunoprecipitation lysis buffer at 37 °C for 6 h. The immunoprecipitation assay was performed using standard protocols, and the protein-bound beads were washed three times. Methanol was used to extract the metabolites.

Trace-level quantitation of metabolites

The immunocomplexes were directly dissolved into 100 μL of 100% acetonitrile. This resulted in a final concentration of approximately 80% acetonitrile due to flow-PBS contamination. The samples were vacuum-concentrated using an EZ2 elite system (Genevac) and stored at -80 °C until further processing. Targeted quantification of these metabolites via LC-MS was conducted using Agilent 1290 Infinity II UHPLC coupled with a ProLab Zirconium Ultra microLC pump, with an Agilent 6495 QQQ-MS operating in the MRM mode. Electrospray ionization coupling was achieved using a prototype microLC ESI source (ProLab). The MRM settings were optimized for S1P using pure standards, and the optimized settings were applied to detect their respective isomers. LC separation was performed on a 100×0.3 mm column with a 1.8 μm Zorbax Eclipse Plus C18 (Agilent). The solvent gradient ranged from 100% buffer A (10 mM ammonium formate in 90:10 water/methanol) to 100% buffer B (10 mM ammonium formate in 90:10 propanol/acetonitrile). The flow rate was set to 5 μL/min. The autosampler temperature was maintained at 5 °C, and the injection volume was 5 μL. Data processing was performed using Agilent MassHunter Software. For each experiment, at least two negative controls were used to assess

background metabolite levels. Metabolites were included only if they were detected above background levels and had a retention time similar to that of the standard qualifier peak. The area under the curve was calculated to evaluate the metabolic differences.

Molecular docking analysis

S1P and YAP

The structure of YAP1 was not fully analysed using nuclear magnetic resonance spectroscopy and X-ray crystallography; therefore, we selected the full-length YAP1 structure predicted using AlphaFold for the next docking step. The 3D structure of S1P was downloaded from PubChem (CID 5283560). Subsequently, the YAP and S1P structures were uploaded to Webina, an AutoDock-based web server (https://durrantlab.pitt.edu/webina/). Among the top 10 docking complex models generated, S1P bound within the central cavity formed at the interface of TEAD-binding, WW structural, SH3 domain-binding, and PDZ domain-binding domains of YAP. The model with the highest binding affinity was chosen for further analysis.

S1P and Spns2

Molecular docking was performed using S1P and Spns2 protein. In this docking model, S1P and Spns2 formed hydrogen bonding interactions at S232, Y235, and G374, and hydrophobic interactions at L332, F366, and W440, with a docking score of -7.3 kcal/mol. The OX40/Spns2 complex was first established, followed by the

participation of S1P in docking with the Spns2 of the OX40/Spns2 complex. In this docking model, S1P and Spns2 formed two hydrogen bonding interactions at S232, and hydrophobic interactions at I238, P239, V378, F437, and W440, with a docking score of -7.2 kcal/mol. The OX40L/OX40/Spns2 complex was first established, followed by the participation of S1P in docking with the Spns2 of theOX40L/OX40/Spns2 complex. In this docking model, S1P and Spns2 formed hydrophobic interactions at S326, S464, S467, and H468, with a docking score of -0.57 kcal/mol.

MST binding assay

MST measurements were conducted using the Monolith NT.115 system (NanoTemper). The full-length YAP WT protein was expressed and purified in *E. coli* cells. The purified YAP WT protein was labelled with Atto 488 fluorescent dye following the manufacturer's instructions. The labelling efficiency was determined to be 1:2 (protein:dye) by measuring the absorbance at 280 and 488 nm. A solution of S1P or S1P-acetate in 0.01 M HEPES (pH=7.4), 0.15 M NaCl, and 0.005% v/v Surfactant P20 was serially diluted, ranging from 1,000 μM to 30 nM, in the presence of 200 nM labelled YAP. After incubation at room temperature for 15 min, samples were loaded into silica capillaries (Polymicro Technologies). Measurements were taken at 22°C with 20% LED and 40% IR-laser power. Additional measurements were performed at IR laser powers of 20% and 60% for comparison. Data analysis was performed using the Nano Temper Analysis software, utilizing the Kd curve fitting

function.

Enzyme-linked immunosorbent assay (ELISA)

Serum samples from CRC patients and healthy controls or cell culture media were collected and stored at -80 ℃ until testing. EGF levels in the serum or cell culture media were measured using a protein-specific ELISA kit (Solarbio, #SEKH-0052), following the manufacturer's instructions. All measurements were performed in duplicate, and the average values were calculated using standard curve analysis.

Luciferase reporter assay

Three kb promoter of OX40 was amplified and cloned into pGL3 basic luciferase reporter vectors. To test whether STAT3 regulates OX40 transcription, the pGL3-OX40 promoter and internal control *Renilla* LUC were co-transfected into 293T cells. Forty-eight hours post-transfection, a luciferase reporter assay was conducted using a Dual-Luciferase Reporter Assay System (Promega, #E1910) following the manufacturer's instructions. Luminescence was measured using a Gen5 microplate reader (BIOTEK, USA).

Reverse-ChIP

An Reverse-ChIP kit (BersinBio, #Bes5005) was used for this experiment. Cells (3×10^8) were cross-linked with 3% formaldehyde for 10 min at room temperature, and 0.125 M glycine was added to terminate the reaction. The cells were scraped and

harvested, and chromatin DNA was sonicated to obtain approximately 500-bp fragments. Biotin-labelled probes for OX40/TNFRSF4 were provided by Bersin Bio, and the probe sequences are listed in Table S6. The mixed probes at a final concentration of 1 nM per probe (pre-denatured at 85 °C for 3 min) were added to the chromatin supernatant followed by hybridization (85 °C for 10 min, 37 °C for 30 min, 70 °C for 5 min, 37 °C for 30 min, 55 °C for 2.5 min, and 37 °C for 60 min). The supernatant was incubated with streptavidin magnetic beads for 2 h at 37 °C. After rinsing five times, elution buffer was added to resuspend the beads, and the proteins were eluted by heating with shaking. Next, the protein samples were subjected to polyacrylamide gel electrophoresis, and binding between the OX40/TNFRSF4 promoter and TFs was verified by western blotting.

ChIP

ChIP assays were performed in HUVECs using a ChIP kit (Beyotime, #P2078) following the manufacturer's instructions. Rabbit IgG was used as a control, and an anti-STAT3 antibody was used to pull down the promoter regions of OX40 genes with the STAT3 regulatory element. The DNA fragments were purified and used for real-time PCR with primers targeting the OX40 promoter. The results are presented as fold-changes and were calculated by dividing the ChIP signals obtained with the anti-STAT3 antibody by those obtained with the IgG control. Primers used for this analysis are listed in Table S6.

Mining the TCGA datasets

The dataset files for COAD were downloaded from the TCGA website. The RNA-seq files provided normalized FPKM values, survival status, and time for each patient. For gene expression analysis, the FPKM values of the indicated genes from tumor samples and corresponding normal tissue samples, if available, were plotted. Statistically significant differences between tumors and NTs were calculated using Student's *t*-test. Gene expression correlations between OX40/TNFRSF4 and CD31, CD34, and VWF levels were assessed using Spearman correlation tests.
