# Supplementary material for: Endothelial OX40 activation facilitates tumor cell escape from T cell surveillance through S1P/YAP-mediated angiogenesis
Source: J Clin Invest. 2025 Mar 3;135(5):e186291. doi: 10.1172/JCI186291 (PMC11870743; doi:10.1172/JCI186291)

Figure 6A

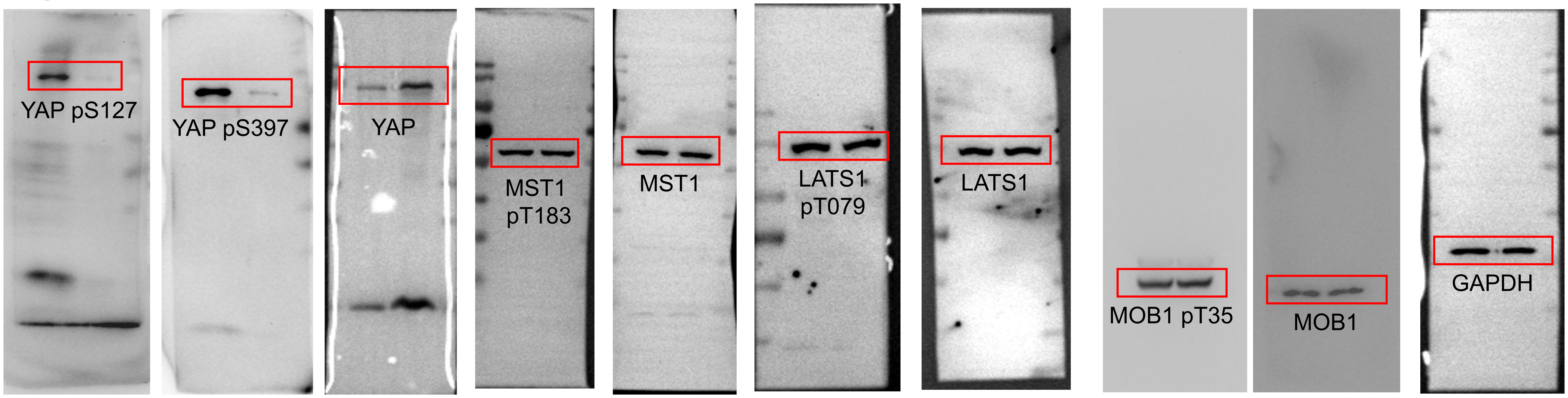

Figure 6B

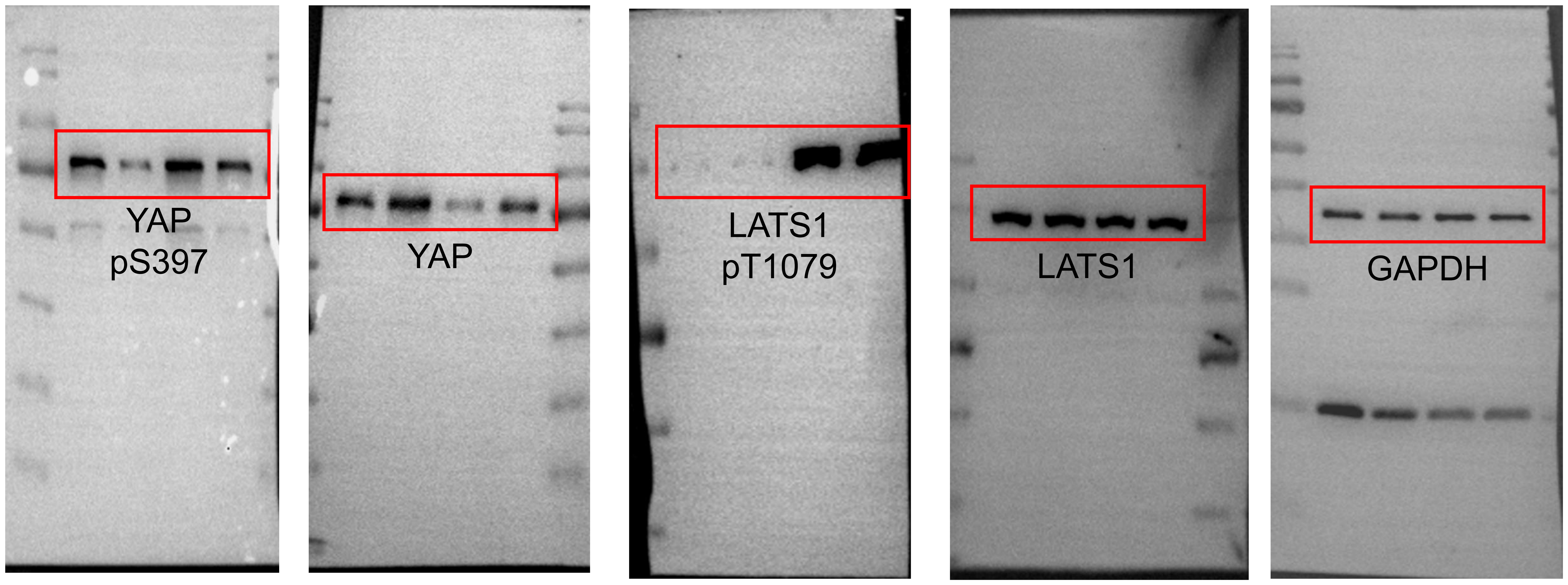

Figure 6C

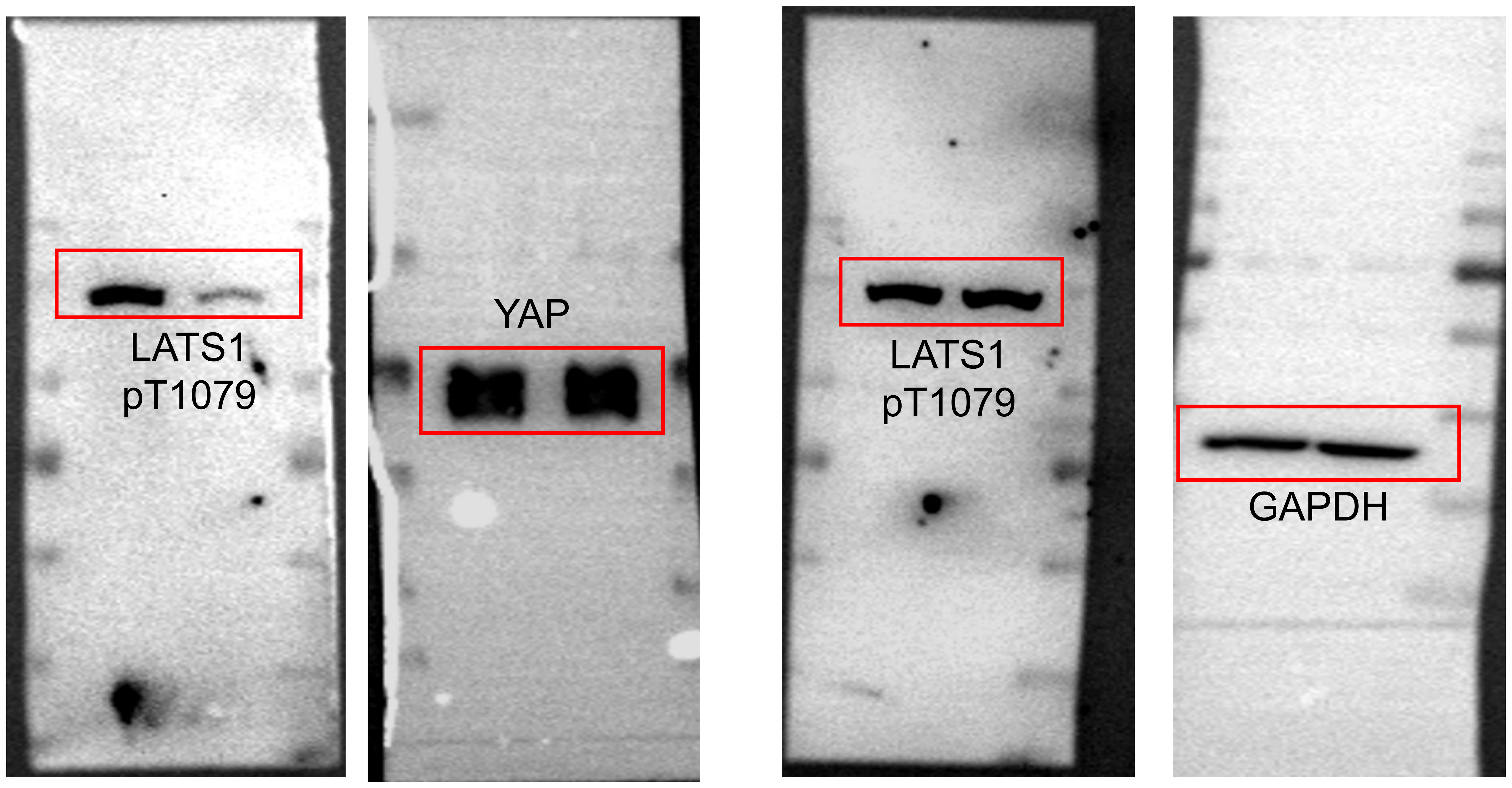

Figure 6G

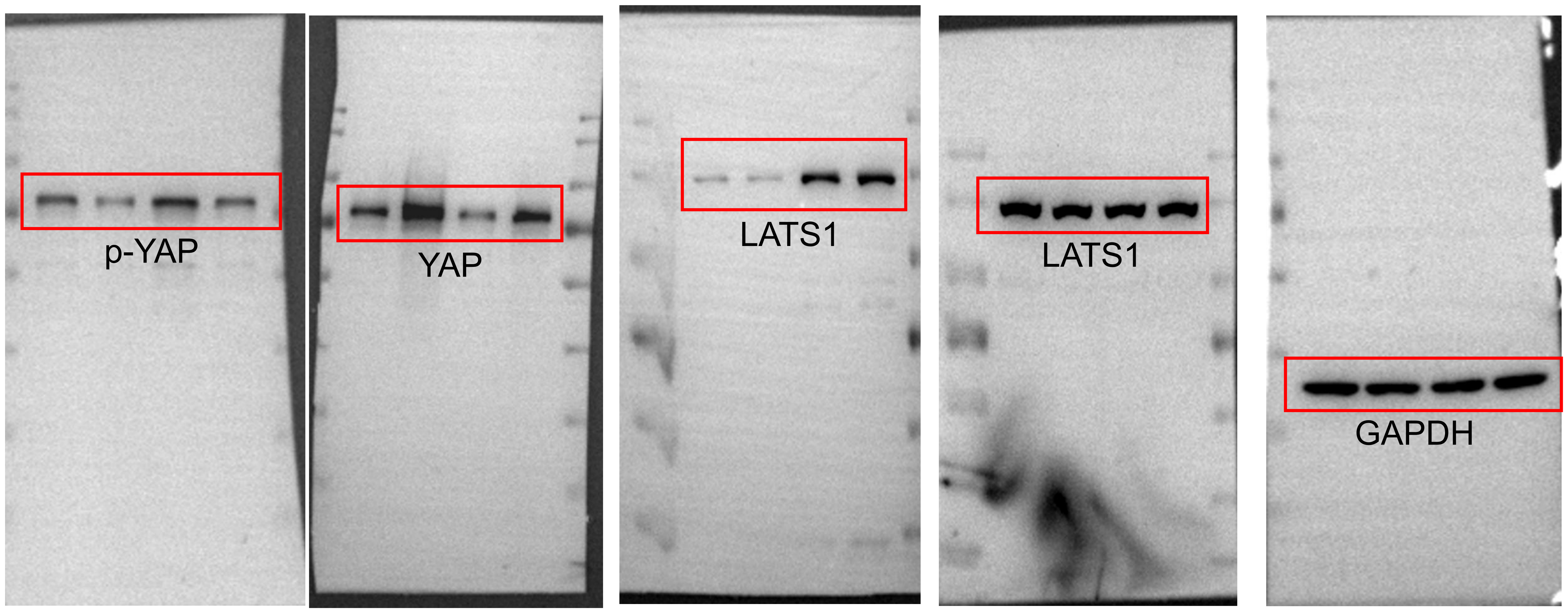

Figure 7D

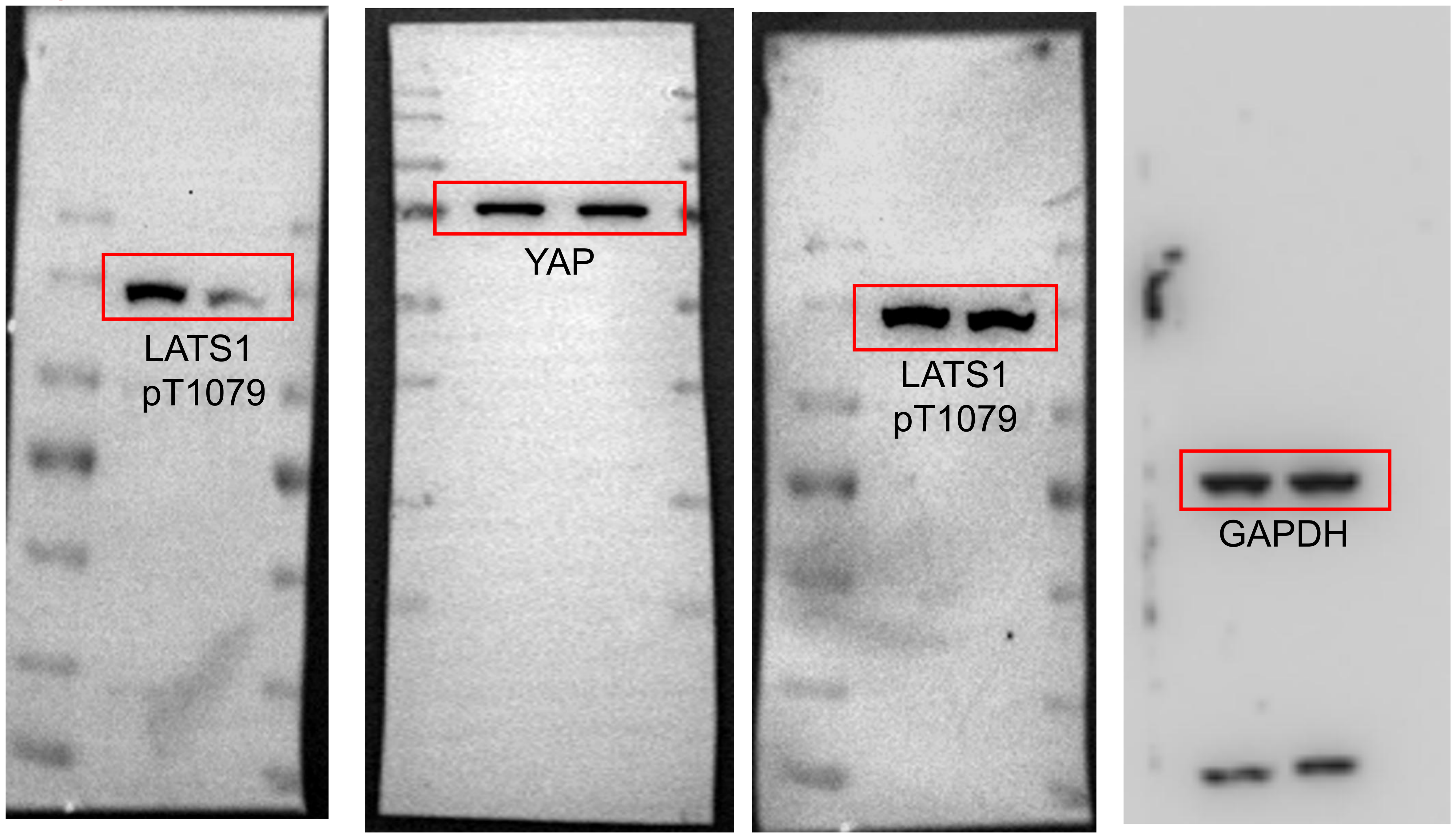

Figure 7F

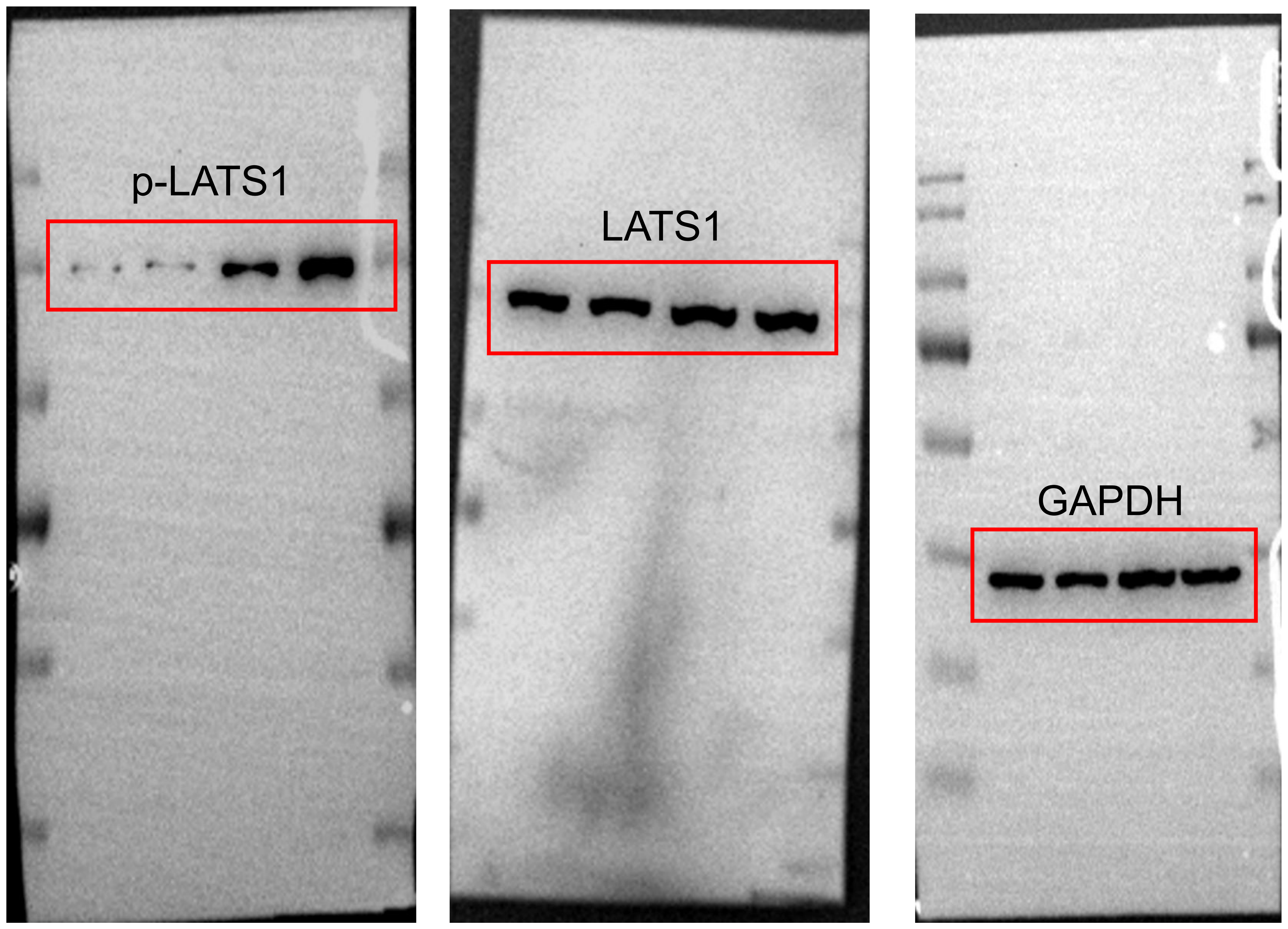

Figure 7G

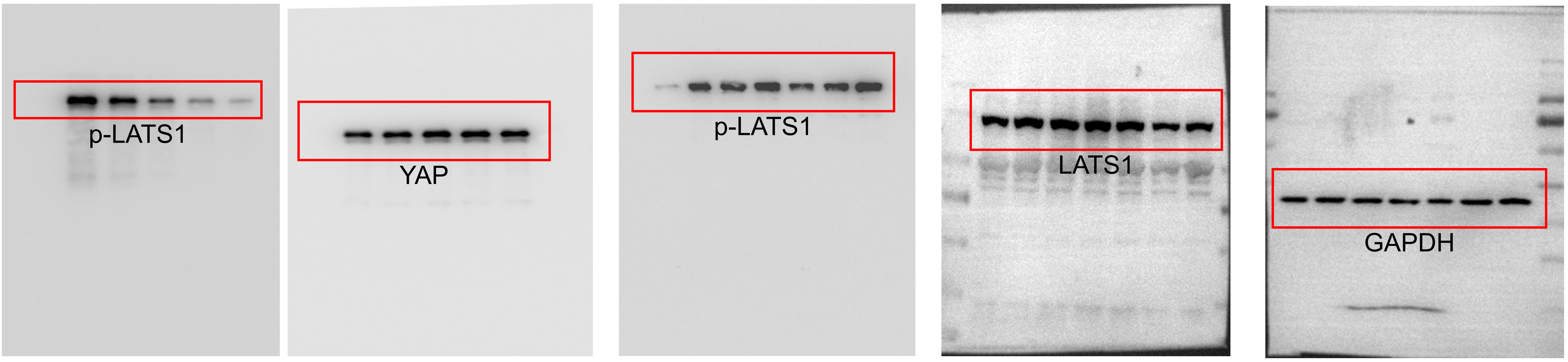

Figure 7H

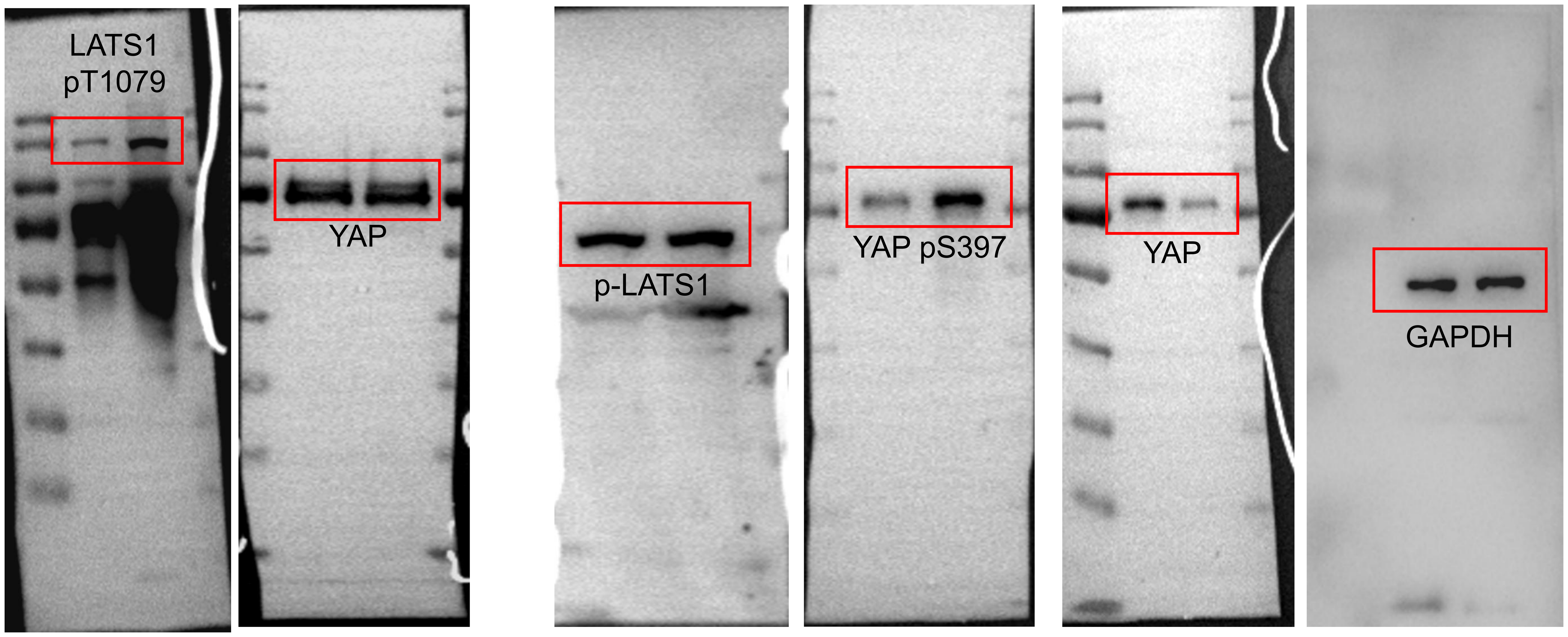

Figure 8D

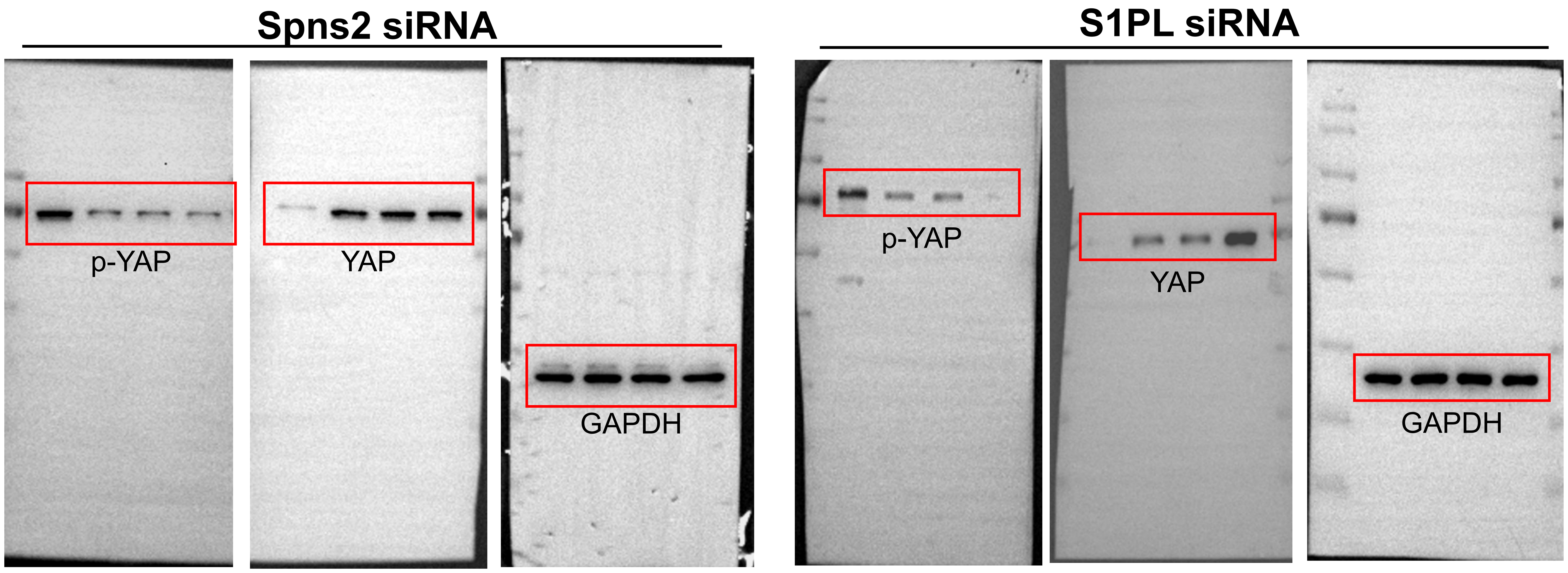

Figure 8D

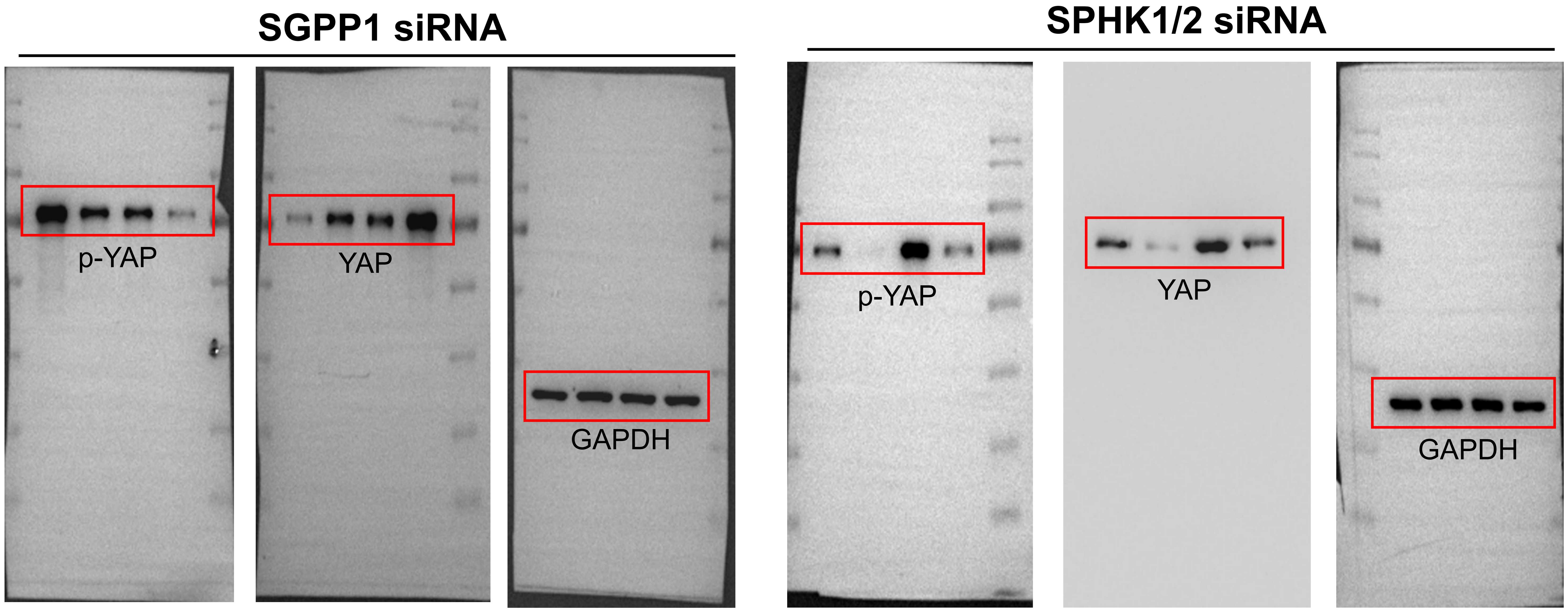

Figure 8F

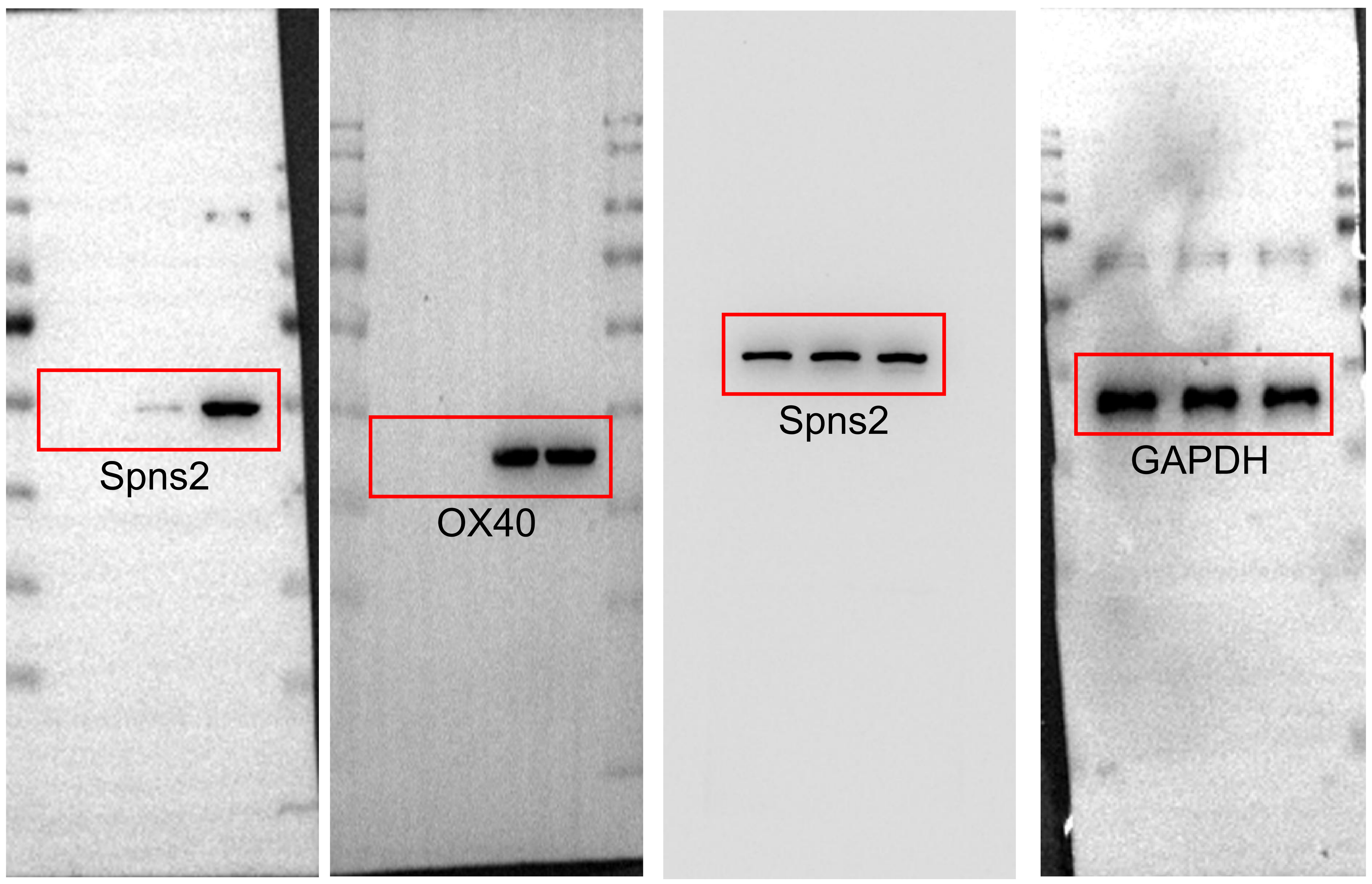

Figure S5A

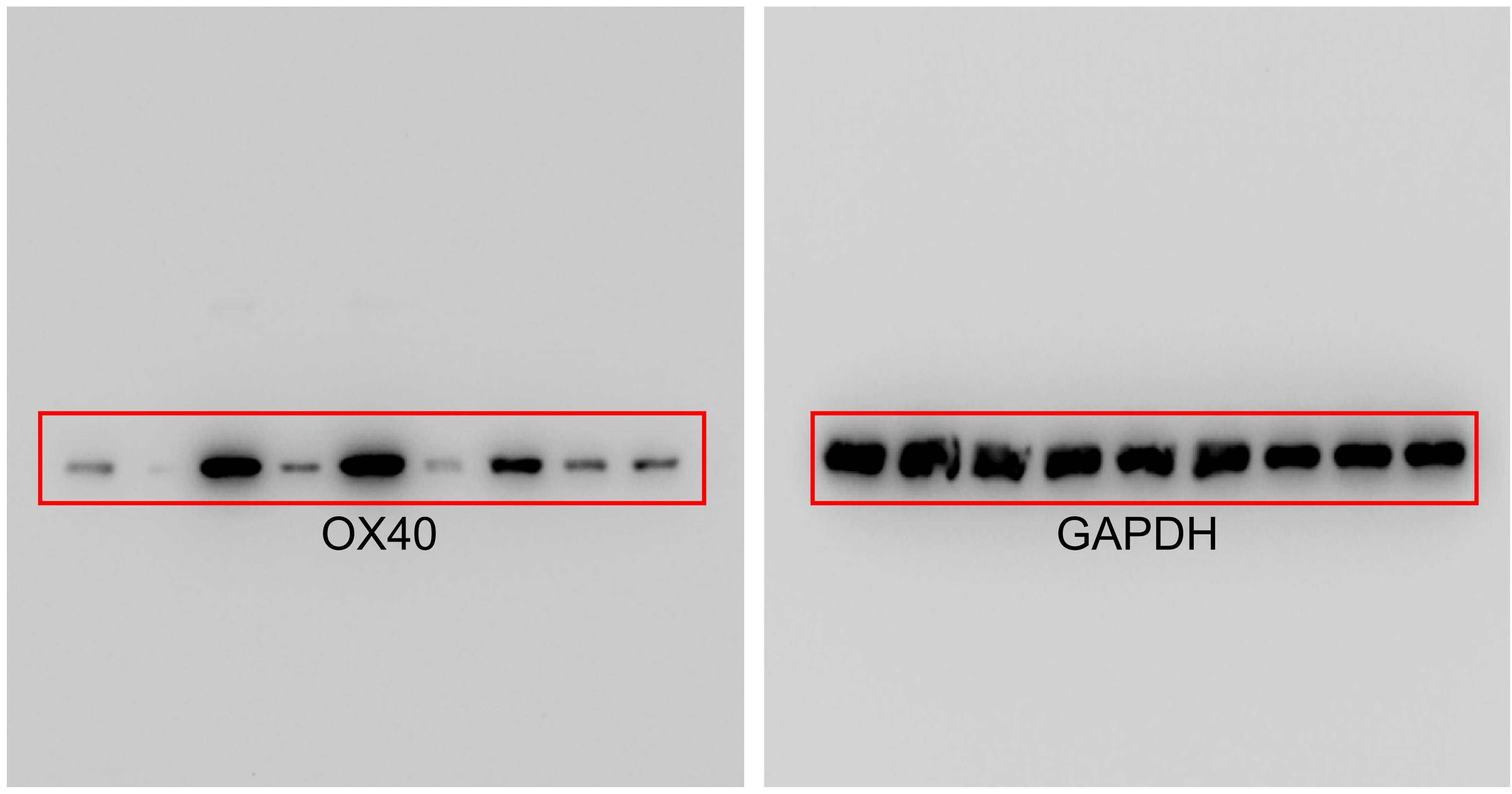

Figure S5B

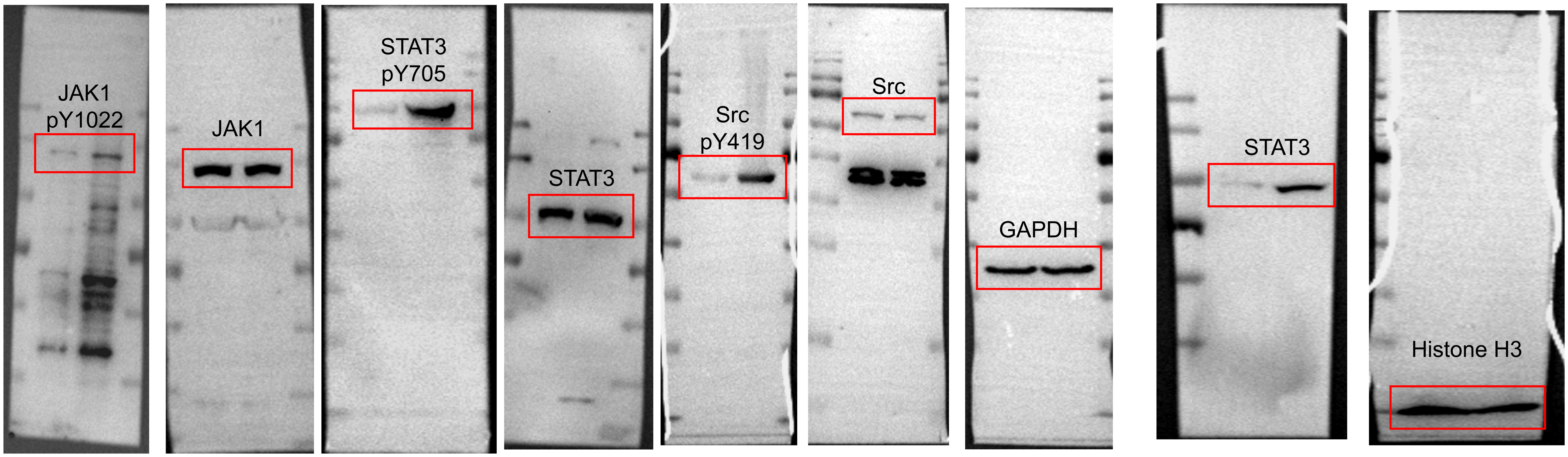

Figure S7A

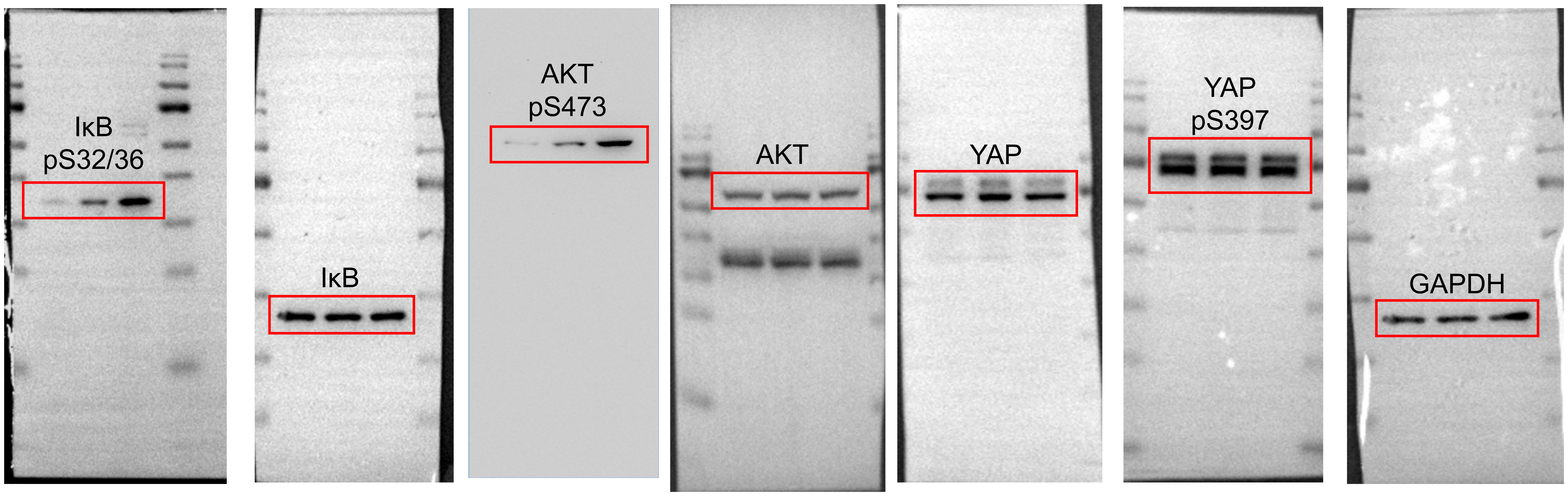

Figure S7B

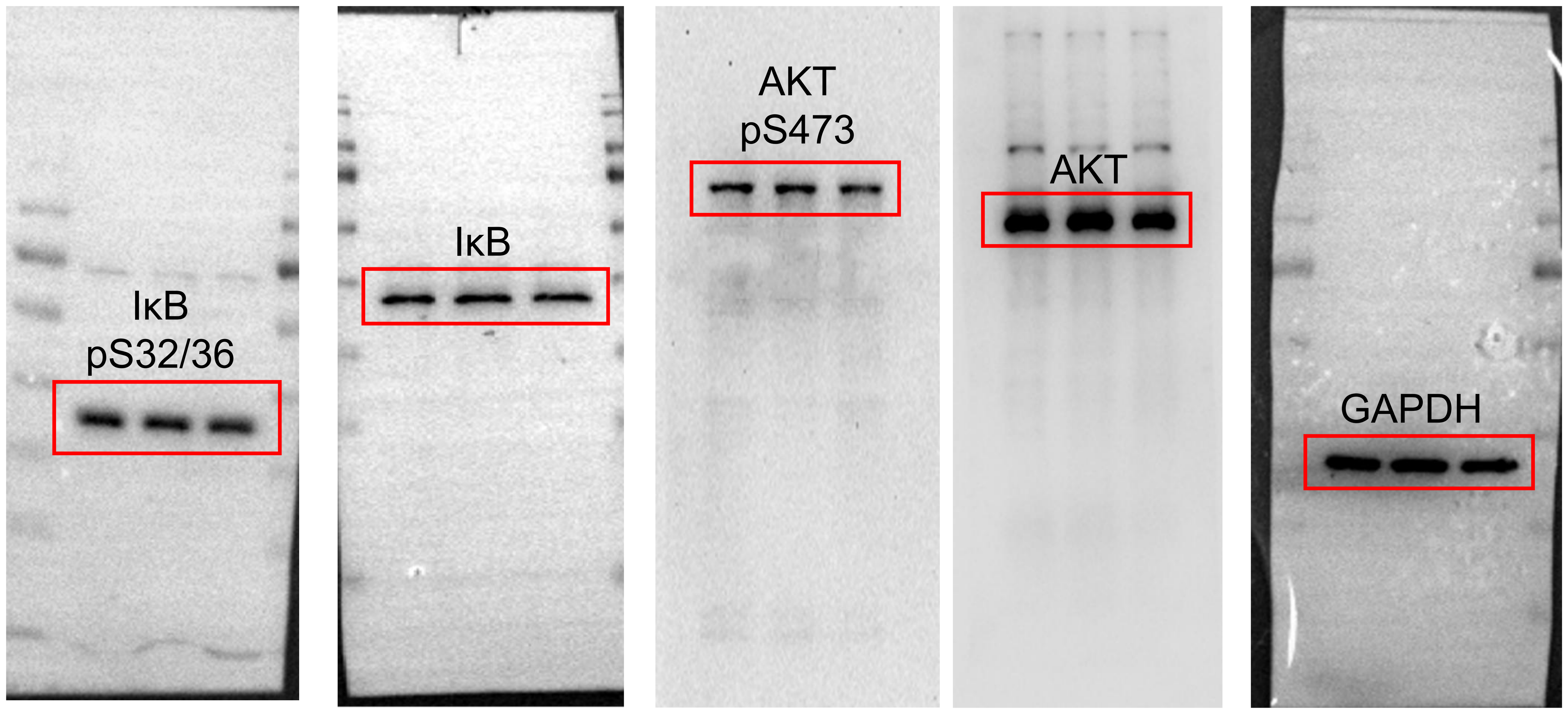

Figure S8B

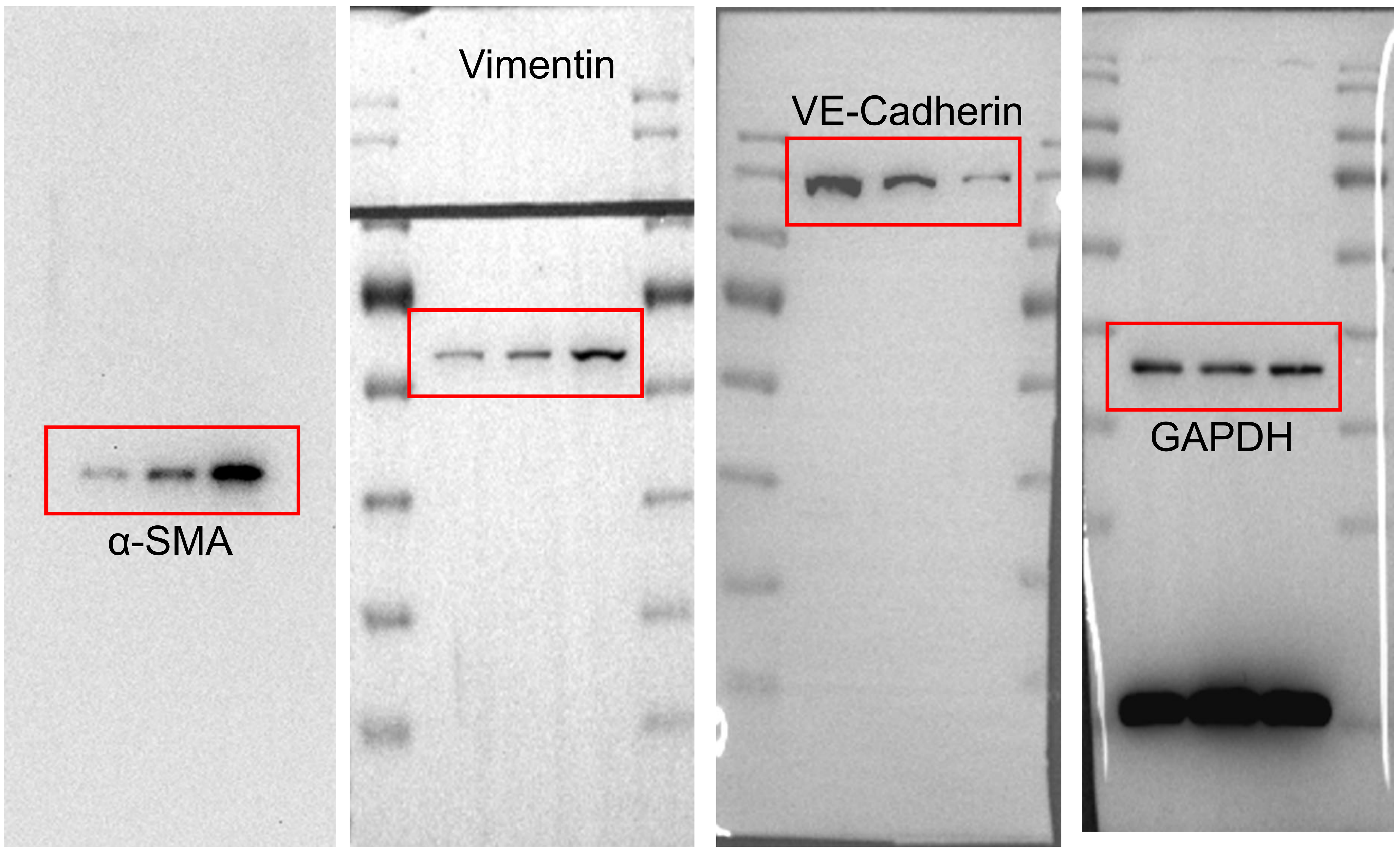

Figure S9A

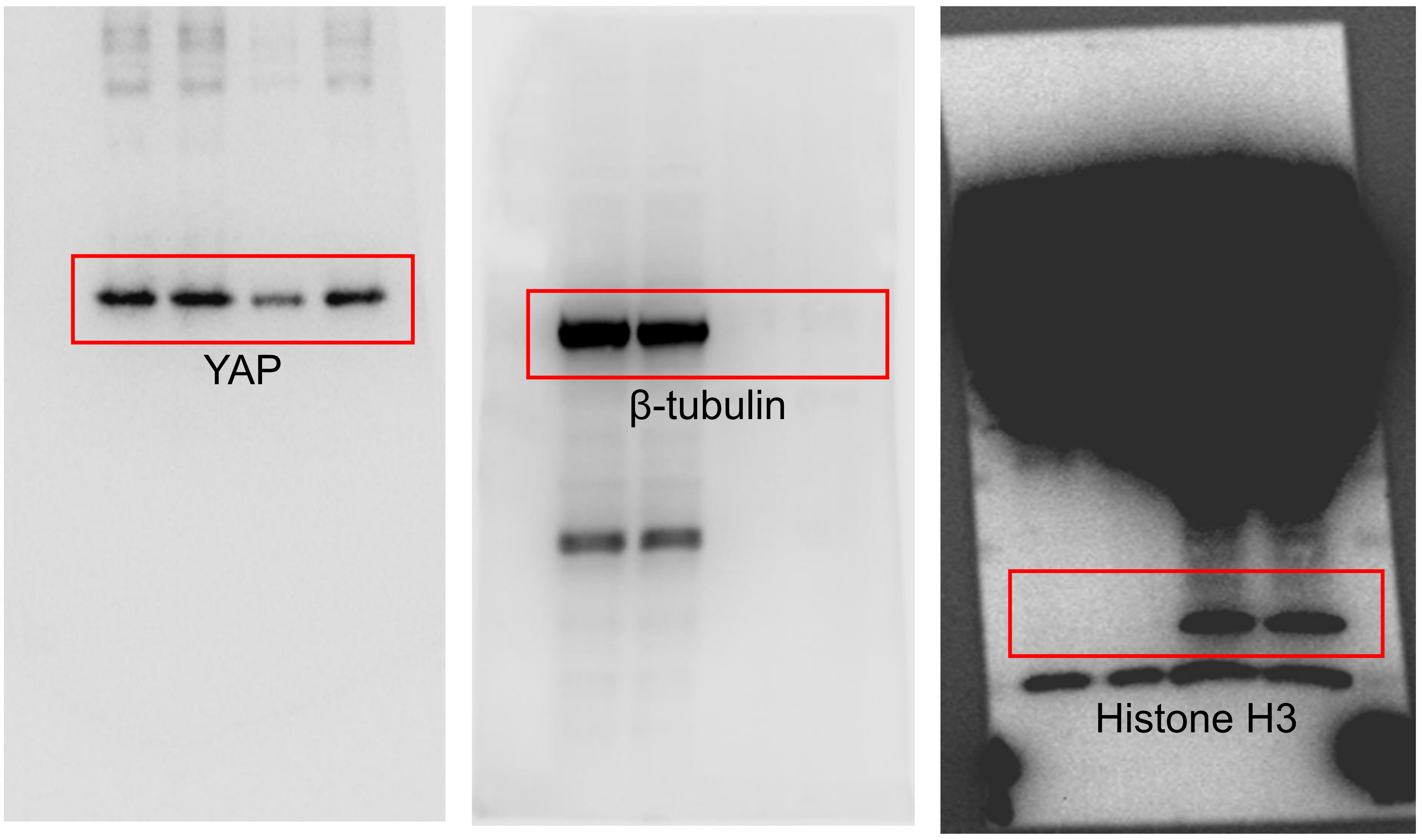

Figure S10B

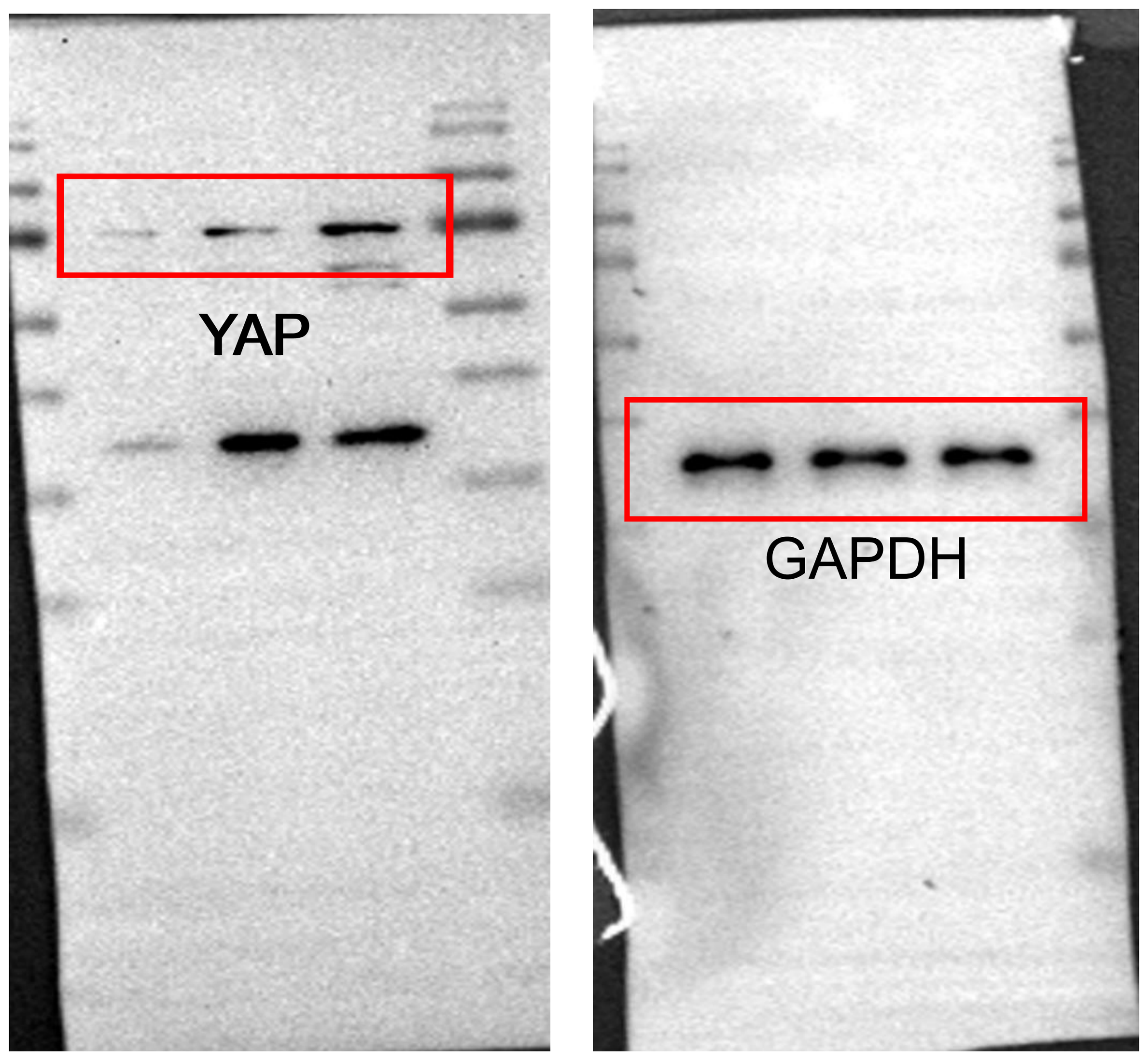

Figure S10C

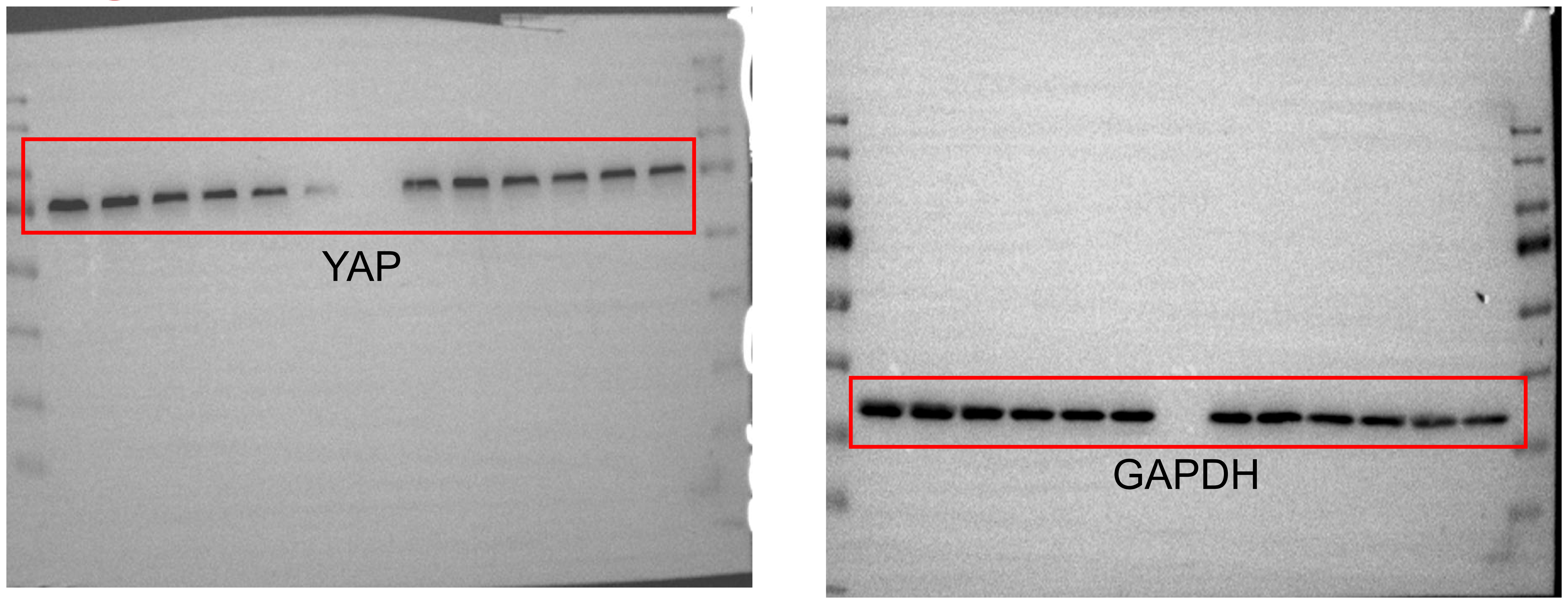

Figure S10D

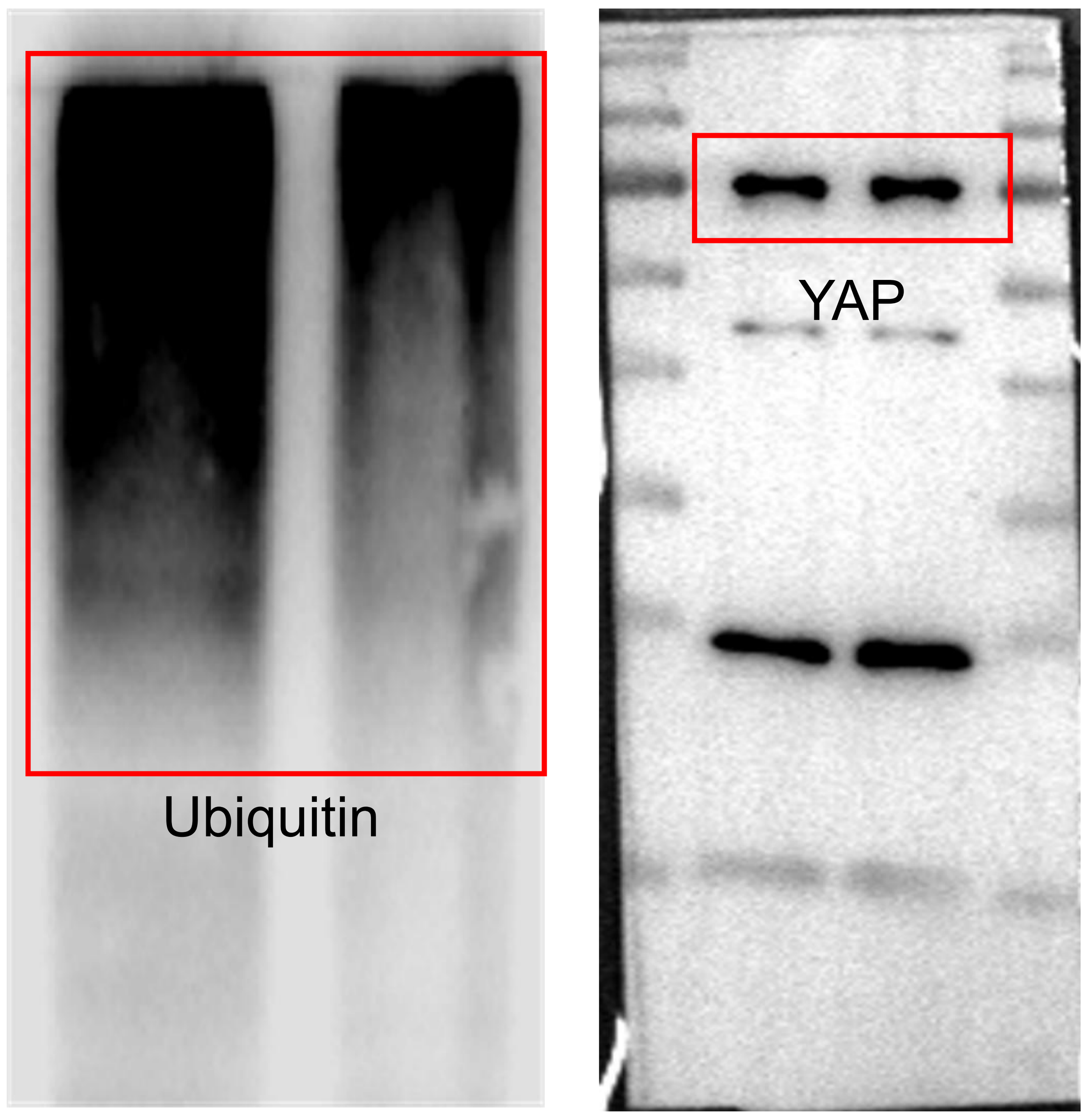

Figure S10E

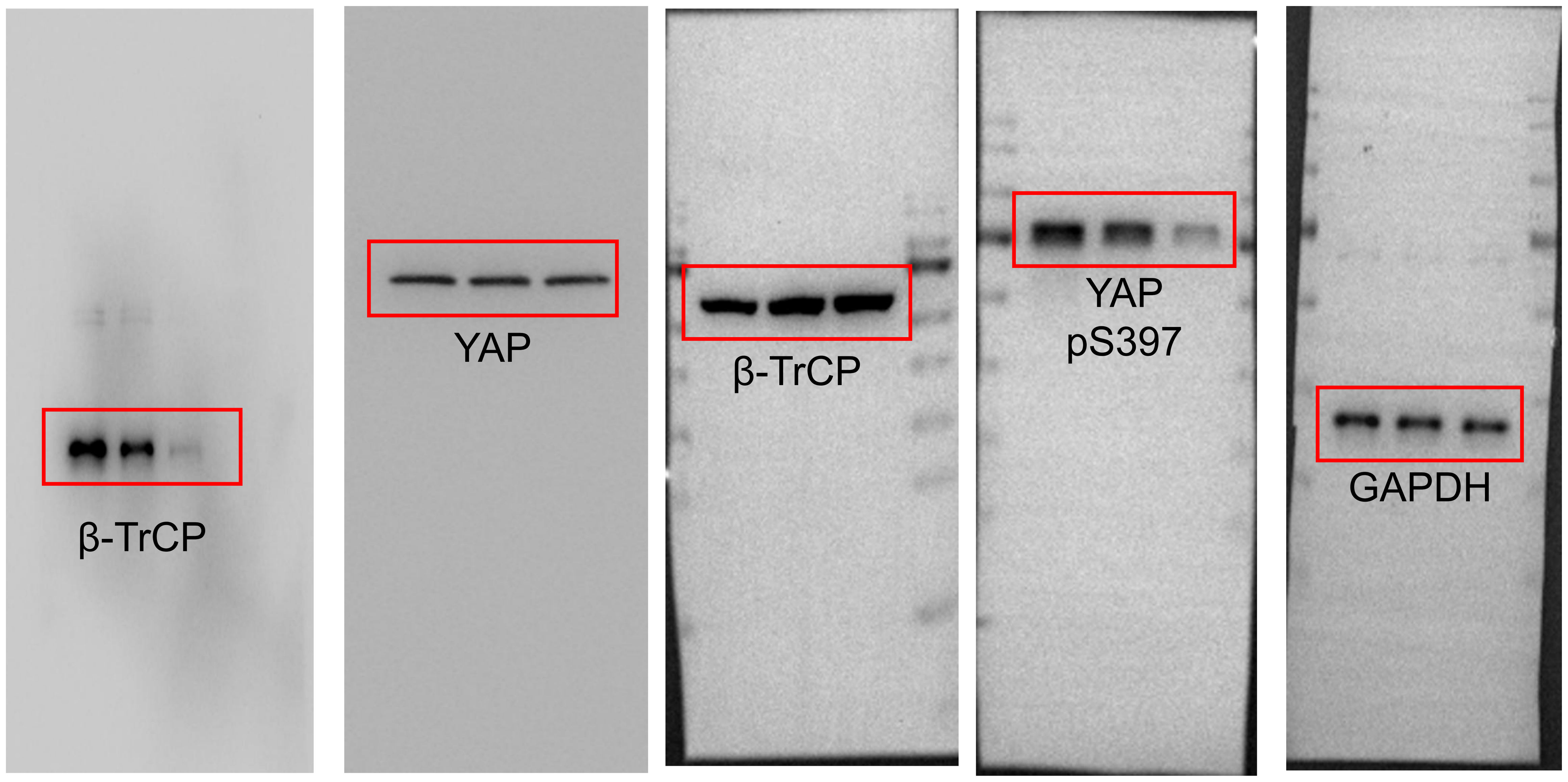

Figure S11B

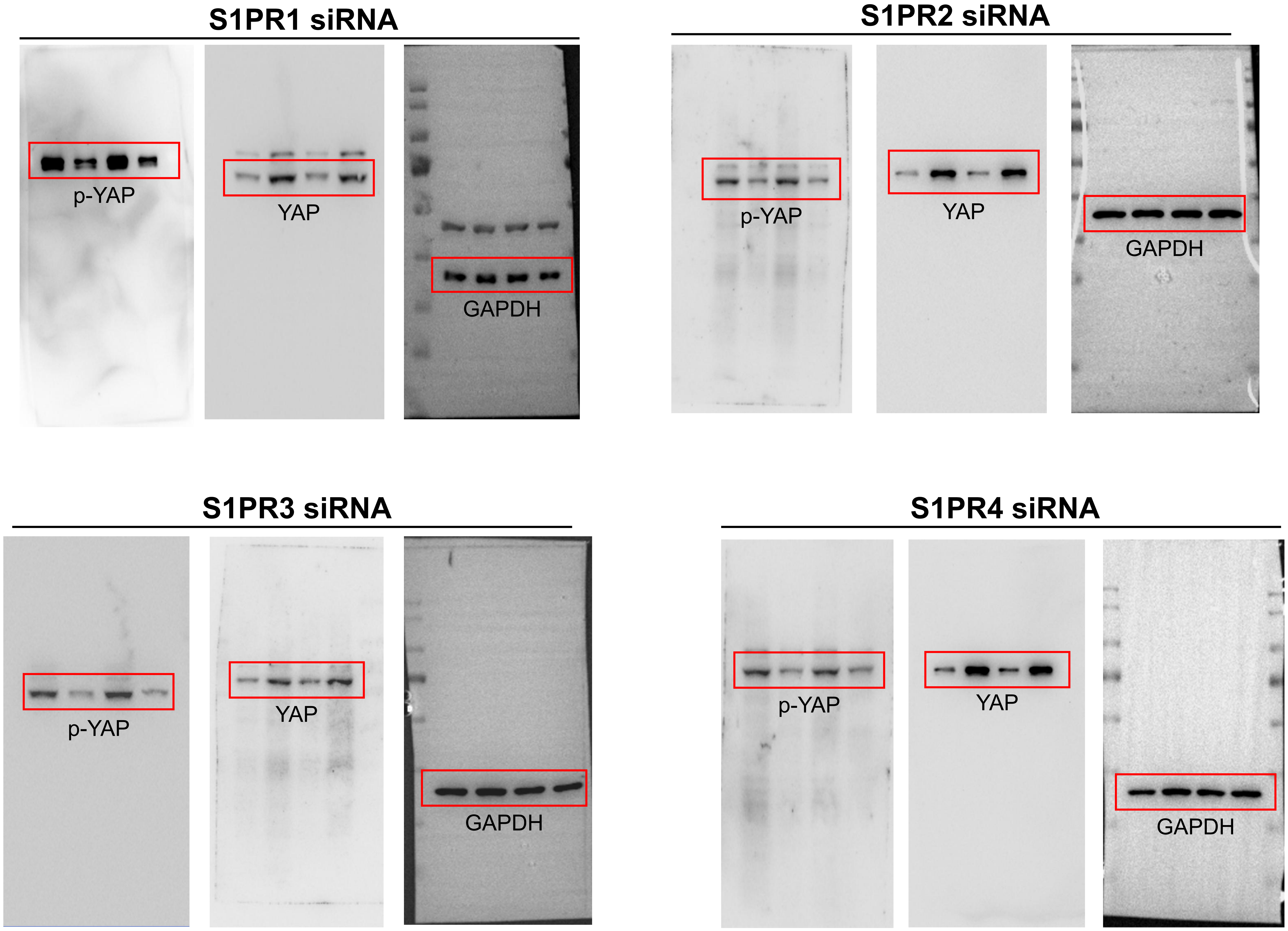

Figure S11B

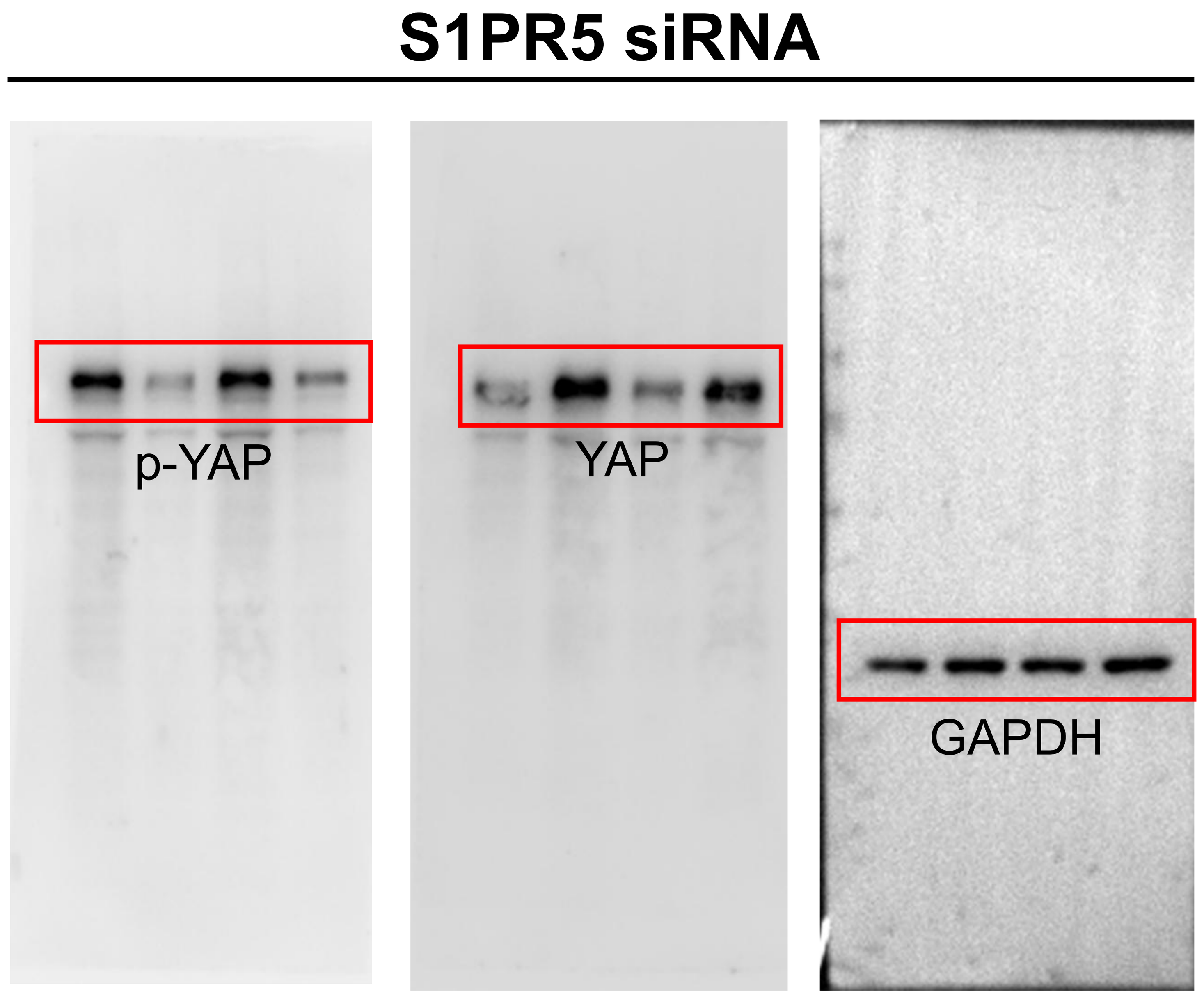

Figure S12A

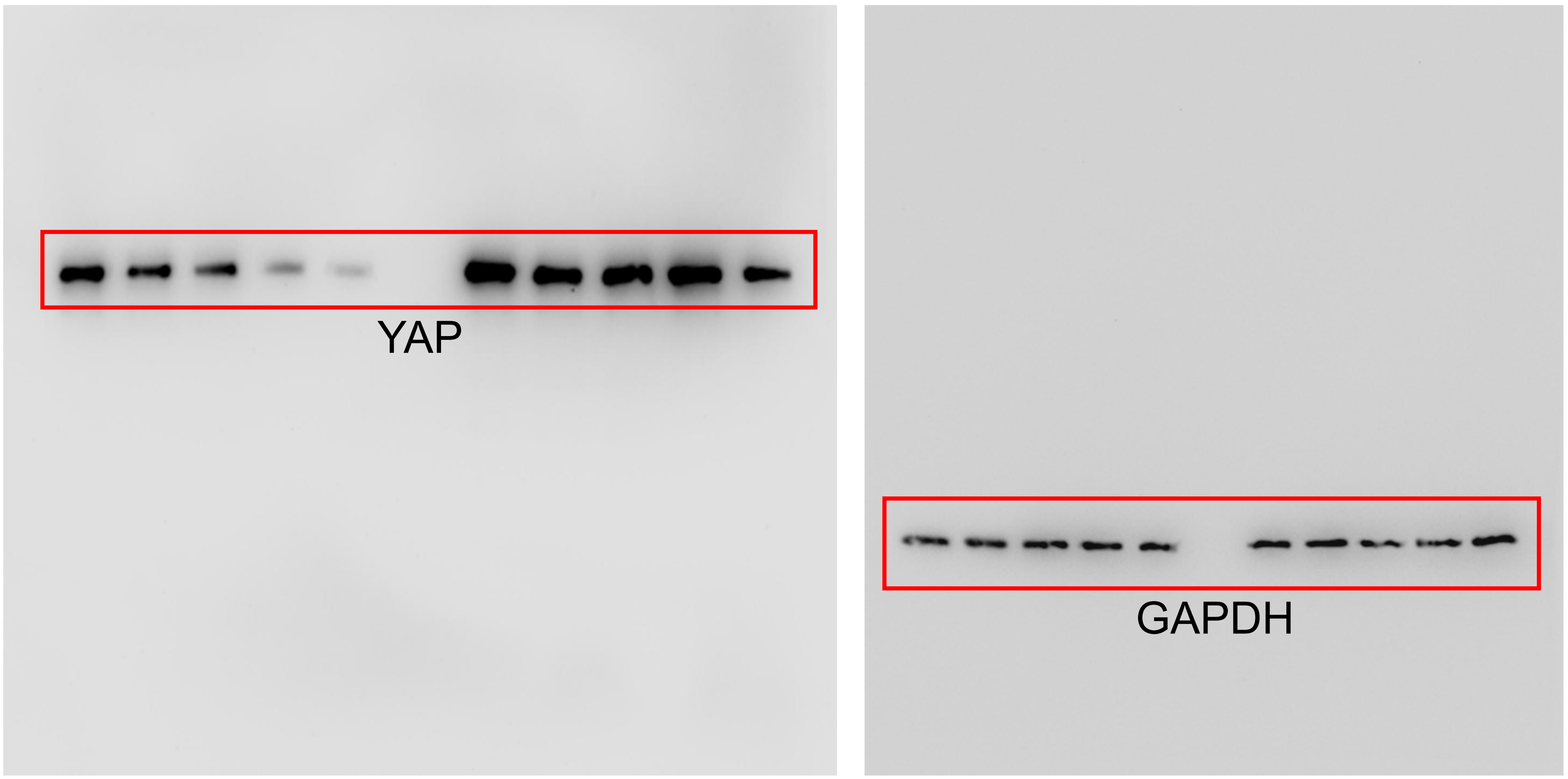

Figure S12B

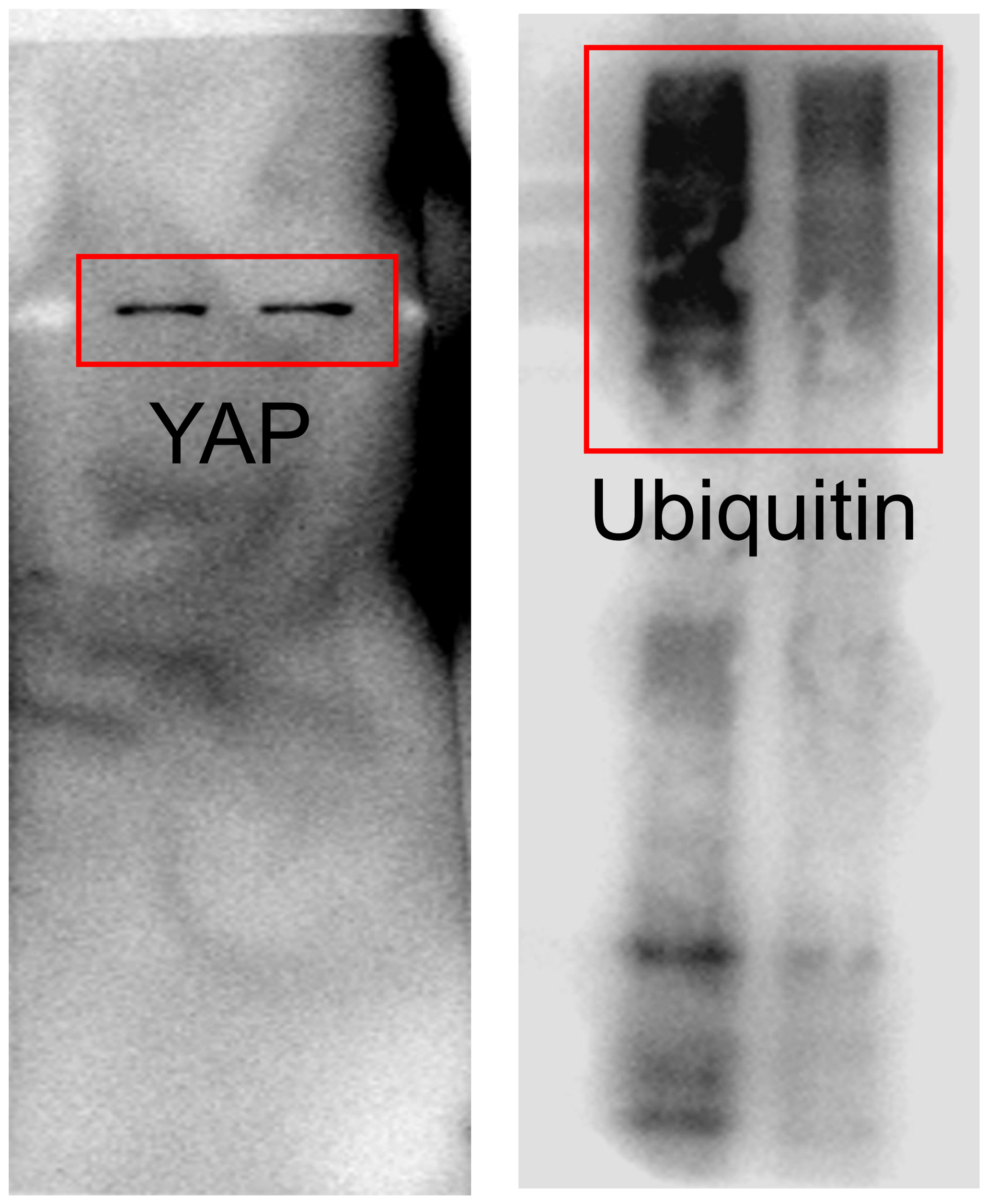

Figure S13C

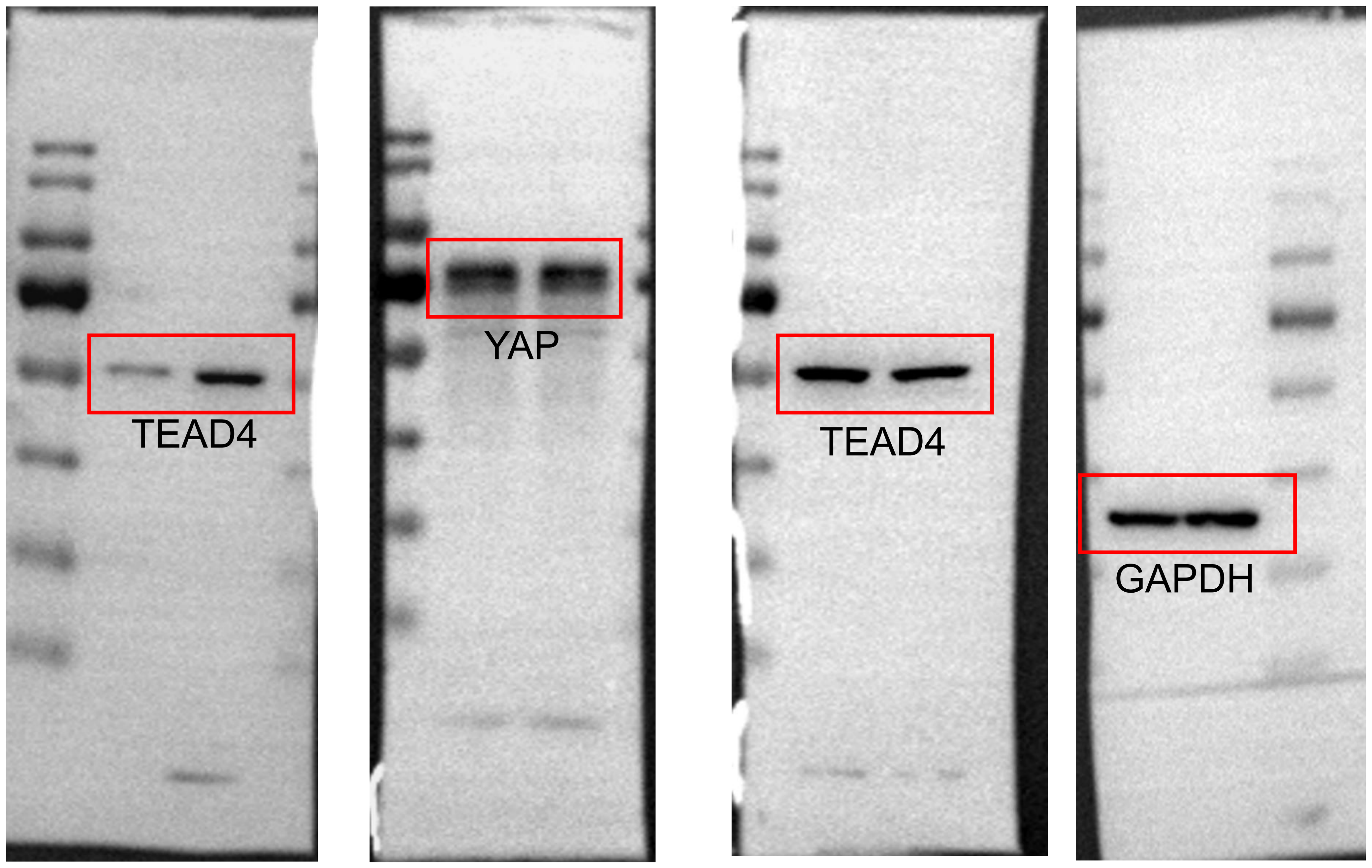

Figure S13D

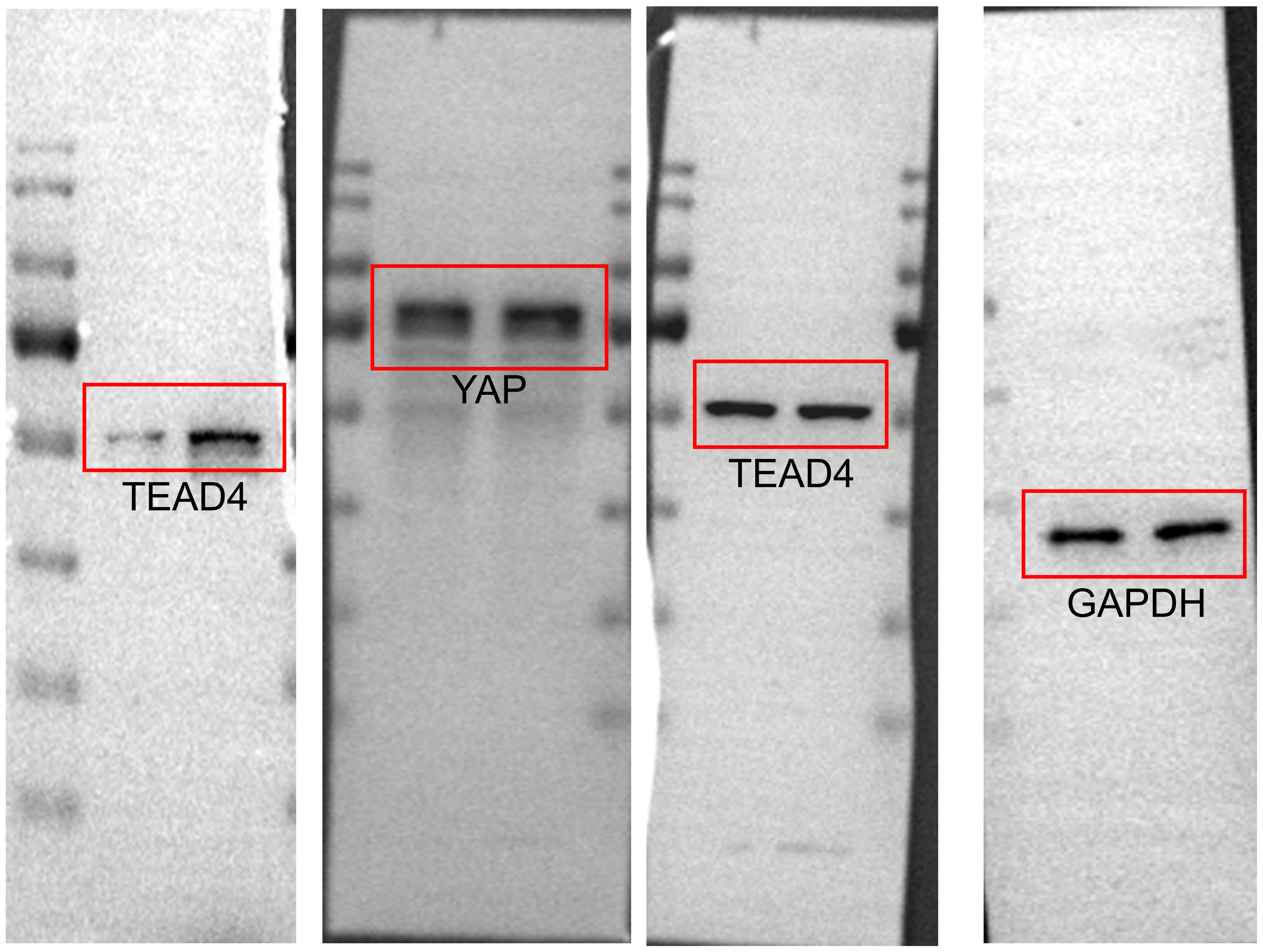

Figure S17A

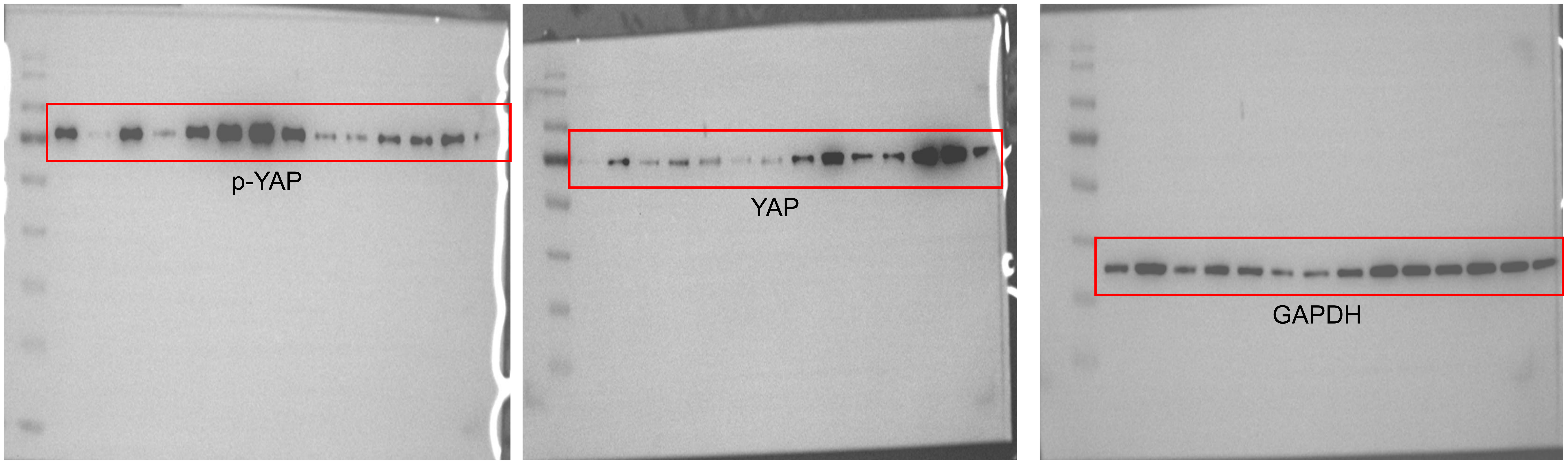

Supplement: Unedited blot and gel images [file jci-135-186291-s062.pdf]
