# Supplementary material for: Endothelial OX40 activation facilitates tumor cell escape from T cell surveillance through S1P/YAP-mediated angiogenesis
Source: J Clin Invest. 2025 Mar 3;135(5):e186291. doi: 10.1172/JCI186291 (PMC11870743; doi:10.1172/JCI186291)
Supplement: Supplemental table 6 [file jci-135-186291-s068.doc]

**Supplemental Table S6. Primers and oligonucleotides used in this study**

| **Primer name** | **Primer sequences（5’ to 3’）** | |
| --- | --- | --- |
| **Primers for qRT-PCR** | | |
| OX40/TNFRSF4 | Forward | GCAATAGCTCGGACGCAATCT |
|  | Reverse | GAGGGTCCCTGTGAGGTTCT |
| CTGF | Forward | CAGCATGGACGTTCGTCTG |
|  | Reverse | AACCACGGTTTGGTCCTTGG |
| CYR61 | Forward | CTCGCCTTAGTCGTCACCC |
|  | Reverse | CGCCGAAGTTGCATTCCAG |
| ANKRD1 | Forward | AGTAGAGGAACTGGTCACTGG |
|  | Reverse | TGTTTCTCGCTTTTCCACTGTT |
| YAP | Forward | TAGCCCTGCGTAGCCAGTTA |
|  | Reverse | TCATGCTTAGTCCACTGTCTGT |
| GAPDH | Forward | GGAGCGAGATCCCTCCAAAAT |
|  | Reverse | GGCTGTTGTCATACTTCTCATGG |
| **Primers for ChIP-PCR** | | |
| OX40-promoter  P1: -663 to -674 | Forward | CCCAGATGGGGCAGAGA |
|  | Reverse | CGCCCTCTCGGCCCCA |
| OX40-promoter  P2: -663 to -674 | Forward | GCCACTTAGGCCCCAG |
|  | Reverse | AGCATCCCAGACAGTGTGTG |
| OX40-promoter  P3: -663 to -674 | Forward | AGGGCGGGTGGCCTCT |
|  | Reverse | ACCAGCCTGTGATTATCCAA |
| **Targeting sequences of siRNAs** | | |
| S1PR1 siRNA | Forward | UGGAAUUUCUUGGUUUUCCAAdTdT |
|  | Reverse | GGAAAACCAAGAAAUUCCACCdTdT |
| S1PR1 siRNA | Forward | GCUAUUCAUUAGAUAGUAAUUdTdT |
|  | Reverse | UUACUAUCUAAUGAAUAGCUGGdTdT |
| S1PR2 siRNA | Forward | UGAAAUUUAUUGUUUUUCCAGdTdT |
|  | Reverse | GGAAAAACAAUAAAUUUCACAdTdG |
| S1PR2 siRNA | Forward | GGCACUGACUAGUCACAUAGCdTdT |
|  | Reverse | UAUGUGACUAGUCAGUGCCUUdTdG |
| S1PR3 siRNA | Forward | GAGUCUUUCAGAUGUACUAAGdTdT |
|  | Reverse | UAGUACAUCUGAAAGACUCUGdTdT |
| S1PR3 siRNA-2 | Forward | CGAUAAUGAUAGUAUUAAUCCdTdT |
|  | Reverse | AUUAAUACUAUCAUUAUCGUUdTdT |
| S1PR4 siRNA-1 | Forward | GGCUCAUUGUUCUGCACUACAdTdT |
|  | Reverse | UAGUGCAGAACAAUGAGCCGGdTdT |
| S1PR4 siRNA-2 | Forward | GCCUGGUGGUGCUGGAGAACUdTdT |
|  | Reverse | UUCUCCAGCACCACCAGGCAGdCdA |
| S1PR5 siRNA-1 | Forward | GGACCUUGUGGGUGAUAUAGAdTdT |
|  | Reverse | UAUAUCACCCACAAGGUCCUUdTdT |
| S1PR5 siRNA-2 | Forward | GAAGACUCCUGCUAAGCAACCdTdT |
|  | Reverse | UUGCUUAGCAGGAGUCUUCAGdTdT |
| Spns2 siRNA-1 | Forward | GAGGCUGUGUCCUCAGUUACCdTdT |
|  | Reverse | UAACUGAGGACACAGCCUCUUdTdT |
| Spns2 siRNA-2 | Forward | GCACUUCUGCUGCAAUCAAGGdTdT |
|  | Reverse | UUGAUUGCAGCAGAAGUGCAGdTdT |
| S1PL siRNA-1 | Forward | GCAGGACAGGACUAUUCUAGCdTdT |
|  | Reverse | UAGAAUAGUCCUGUCCUGCAGdTdT |
| S1PL siRNA-2 | Forward | GAUUCAAAUAUGUCACCAAAGdTdT |
|  | Reverse | UUGGUGACAUAUUUGAAUCUGdTdT |
| SGPP1 siRNA-1 | Forward | GAAUGCAUAUGCUACUAUACAdTdT |
|  | Reverse | UAUAGUAGCAUAUGCAUUCUGdTdT |
| SGPP1 siRNA-2 | Forward | GCAUGUGGAUCUCAUGUUACUdTdT |
|  | Reverse | UAACAUGAGAUCCACAUGCAAdTdT |
| SPHK1 siRNA-1 | Forward | CGACGAGGACUUUGUGCUAGUdTdT |
|  | Reverse | UAGCACAAAGUCCUCGUCGGGdTdT |
| SPHK1 siRNA-2 | Forward | CGAACCAAAUCCAAAUAAAGUdTdT |
|  | Reverse | UUUAUUUGGAUUUGGUUCGUGdTdT |
| SPHK2 siRNA-1 | Forward | GGUUGCUUCUAUUGGUCAAUCdTdT |
|  | Reverse | UUGACCAAUAGAAGCAACCGGdTdT |
| SPHK2 siRNA-2 | Forward | GGCGCUAGGAUUUGCACUAAUdTdT |
|  | Reverse | UAGUGCAAAUCCUAGCGCCGGdTdT |
| EGF siRNA-1 | Forward | GUUACAAGAUUGUAAGUAAAUdTdT |
|  | Reverse | UUACUUACAAUCUUGUAACUGdTdT |
| EGF siRNA-2 | Forward | CGACUAAUCACCUACUCAAUGdTdT |
|  | Reverse | UUGAGUAGGUGAUUAGUCGUAdTdT |
| EGFR siRNA-1 | Forward | AAAUGAUCUUCAAAAGUGCCCdTdT |
|  | Reverse | GCACUUUUGAAGAUCAUUUUCdTdT |
| EGFR siRNA-2 | Forward | AAAAUGAUCUUCAAAAGUGCCdTdT |
|  | Reverse | CACUUUUGAAGAUCAUUUUCUdTdT |
| STAT3 siRNA-1 | Forward | UGAUUCUUCGUAGAUUGUGCUdTdT |
|  | Reverse | CACAAUCUACGAAGAAUCAAGdTdT |
| STAT3 siRNA-2 | Forward | UGAAGAAACUGCUUGAUUCUUdTdT |
|  | Reverse | GAAUCAAGCAGUUUCUUCAGAdTdT |
| Control siRNA | Forward | CCUACGCCACCAAUUUCGUdTdT |
|  | Reverse | ACGAAAUUGGUGGCGUAGGdTdT |
| **Reverse-ChIP probe sequences** | | |
| OX40/TNFRSF4  Promoter (304-328) | TTAAGGTCACAGGTCCAAGAAAGCC | |
| OX40/TNFRSF4  Promoter (527-551 ) | AATTGGTTCCTTGGAGGTTCAGGTG | |
| OX40/TNFRSF4  Promoter (739-763 ) | GACTGAGGGTCCCTAAGTTGGTCAC | |
| OX40/TNFRSF4  Promoter (960-986 ) | ATAACTGTGACCAGCCTGTGATTATCC | |
| OX40/TNFRSF4  Promoter (1214-1238 ) | GCTGAACTGTGCCCCTTAAATTCCT | |
| OX40/TNFRSF4  Promoter (1616-16640 ) | CAAGACTAGCTCTGAGCAGCAGTGA | |
| OX40/TNFRSF4  Promoter (1821-1846 ) | TTATTCTGCCTTCTGACCTCTTCTGC | |
| **Mice genotyping primers and probs** | | |
| ***OX40^fl/fl^;Cd31^cre/-^* mice** | | |
| 5'-arm | T012796-F1 | AAGCCATCTCTCCTCCCTATGCA |
|  | T012796-R1 | AGAGAGAGAGAGGGAAATGCCCACT |
| 3'-arm | T012796-F2 | GGGGAATAGTAACCAAAGAAGTGCC |
|  | T012796-R2 | CATATAGGAACTTCTGGTGGTTGGC |
| ***OX40^ki/ki^;Cd31^cre/-^* mice** | | |
| Loxp-1 | Forward | CCCACCTTGGTGACTCCTGAG |
|  | Reverse | CTTTATTAGCCAGAAGTCAGATGC |
| Loxp-2 | Forward | AGATCTGCAAGCTAATTCCTGC |
|  | Reverse | TTTAACACAGTTGAGCCGCCTT |
| WT | Forward | CACTTGCTCTCCCAAAGTCGCTC |
|  | Reverse | ATACTCCGAGGCGGATCACAA |
| Cre | Forward | GGCAACGTGCTGGTTATTGTG |
|  | Reverse | TTTAACACAGTTGAGCCGCCTT |
